# SCTR–Expressing Astrocyte–Serotonergic Neuron Signaling Drives Sexual Dimorphism in Depression Through KAT7/H4ac–Foxc2 Epigenetic Axis in an AMPK‐Dependent Manner

**DOI:** 10.1002/advs.77985

**Published:** 2026-09-27

**Authors:** Fantao Meng, Jing Liu, Juanjuan Dai, Min Wu, Wentao Wang, Mengdi Zhang, Shujun Jiang, Yifan Cao, Lihong Xu, Lin Du, Dan Wang, Di Zhao, Gaofeng Qin, Dong Wang, Wei Li, Chen Li

**Affiliations:** ^1^ Department of Rehabilitation Medicine Binzhou Medical University Hospital Binzhou Shandong China; ^2^ Medical Research Center Binzhou Medical University Hospital Binzhou Shandong China; ^3^ Department of Psychology Binzhou Medical University Hospital Binzhou Shandong China; ^4^ Neurosurgery Binzhou Medical University Hospital Binzhou Shandong China; ^5^ Department of Physiology Binzhou Medical University Shandong China

**Keywords:** AMPKα1/2, astrocyte–serotonergic neuron communication, depression, Foxc2, neuronal excitability, SCTR signaling, serotonin neurotransmission

## Abstract

Peripheral hormonal dysfunction is associated with multiple neurological disorders, yet how astrocytes sense circulating stress‐related hormones to influence depression pathogenesis remains incompletely understood. Here, we showed that disruption of secretin receptor (SCTR) signaling in dorsal raphe nucleus (DRN) astrocytes promotes depression‐like behaviors. We identified AMPKα1 as a key downstream effector of astrocytic SCTR signaling and demonstrate its role in regulating astrocytic secretion of metallothionein‐1 (Mt1) and downstream megalin‐mediated AMPKα2 signaling in serotonergic neurons. We further identified the transcription factor Foxc2 as a downstream target of neuronal AMPKα2 that modulates depressive‐like behaviors through suppression of serotonergic activity and reduced neuronal excitability. Mechanistically, AMPKα2 regulates phosphorylation of the acetyltransferase KAT7, thereby modulating histone H4 acetylation and Foxc2 transcription. Together, these findings delineate an astrocyte–neuron signaling axis linking SCTR–AMPKα1 signaling in astrocytes to epigenetic regulation of Foxc2 by AMPKα2–KAT7/H4ac signaling in serotonergic neurons, providing fundamental insight into circuit‐level mechanisms underlying depression.

## Graphical abstract

Astrocytic SCTR modulates depressive‐related phenotypes via AMPKα1, whereas lowered Mt1 blunts Megalin‐dependent AMPKα2 signaling in serotonergic neurons. Diminished AMPKα2 hinders KAT7 nuclear translocation, curtails KAT7 occupancy and H4K8ac at the Foxc2 promoter, and represses Foxc2 transcription, revealing cell‐type‐specific AMPK‐linked epigenetic mechanisms underlying depression.

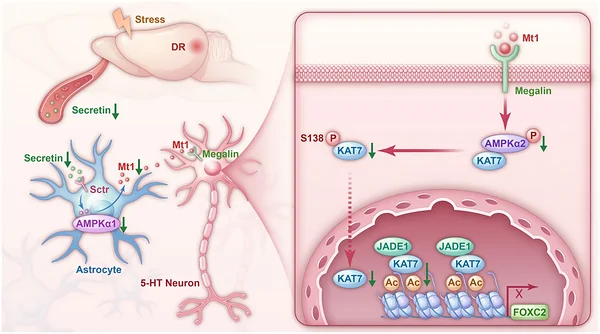

## 1 Introduction

Depressive disorder is a common mental illness associated with substantial functional disability and elevated suicide risk. Its clinical features include persistent sadness, anhedonia, and impairments in cognitive and emotional processing. Globally, more than 300 million individuals are affected by depression, underscoring its significant public health and economic burden [1, 2]. Although a comprehensive biopsychosocial approach is recommended, moderate to severe depression is primarily treated with antidepressant medications [3, 4]. However, despite their widespread use, approximately one‐third to one‐half of patients fail to respond adequately to multiple antidepressant trials [5]. Thus, effective clinical management of depression remains a major challenge. Over recent decades, extensive research has sought to elucidate the etiology of depression [6, 7]. Genetic factors account for an estimated 30%–40% of disease risk [8, 9], and depression is widely considered to arise from a complex interaction between genetic vulnerability and environmental stressors [10]. Nevertheless, the underlying pathophysiological mechanisms remain incompletely understood.

Circulating hormones play a critical role in mental disorders, highlighting bidirectional communication between peripheral tissues and the central nervous system (CNS) [11]. Accumulating evidence implicates peripheral organs, including adipose tissue [12, 13], liver [14], and intestine [15], in the pathogenesis of depression. Secretin (SCT), a classic gastrointestinal hormone [16, 17, 18], has recently been recognized to act through its receptor (SCTR), which is expressed in multiple brain regions [19, 20], suggesting a role in CNS signaling [21]. Disruption of SCTR signaling has been linked to social and behavioral deficits, potentially through alterations in synaptic plasticity [22, 23]. However, whether SCTR signaling contributes to depression and the mechanisms involved remain poorly defined.

The dorsal raphe nucleus (DRN), a principal source of serotonergic innervation to the forebrain and a highly stress‐sensitive brain region [24, 25, 26], is critically involved in the pathophysiology of depression [27, 28, 29]. AMP‐activated protein kinase (AMPK) is a master regulator of cellular energy homeostasis and exerts neuroprotective effects in the brain [30]. Emerging evidence indicates that chronic unpredictable stress (CUS), a commonly used rodent model of depression, reduces phosphorylated (p‐AMPKα) levels in the brain, an effect that can be reversed by multiple classes of antidepressants [29, 31, 32]. However, the precise mechanistic role of AMPK dysregulation in depression remains unclear.

Here, we showed that disruption of SCTR signaling in DRN astrocytes promotes depression‐like behaviors. We identified AMPKα1 as a key downstream effector of astrocytic SCTR signaling and demonstrated its role in regulating astrocytic secretion of metallothionein‐1 (Mt1) and downstream megalin‐mediated AMPKα2 signaling in serotonergic neurons. We further identified the transcription factor Foxc2 as a downstream target of neuronal AMPKα2 that modulates depressive‐like behaviors through suppression of serotonergic signaling and reduced neuronal excitability. Mechanistically, AMPKα2 regulates phosphorylation of the acetyltransferase KAT7, leading to altered histone H4 acetylation and transcriptional regulation of Foxc2.

## 2 Results

### 2.1 Impaired Secretin/SCTR Signaling Promotes Depression‐Like Behaviors

**FIGURE 1:**
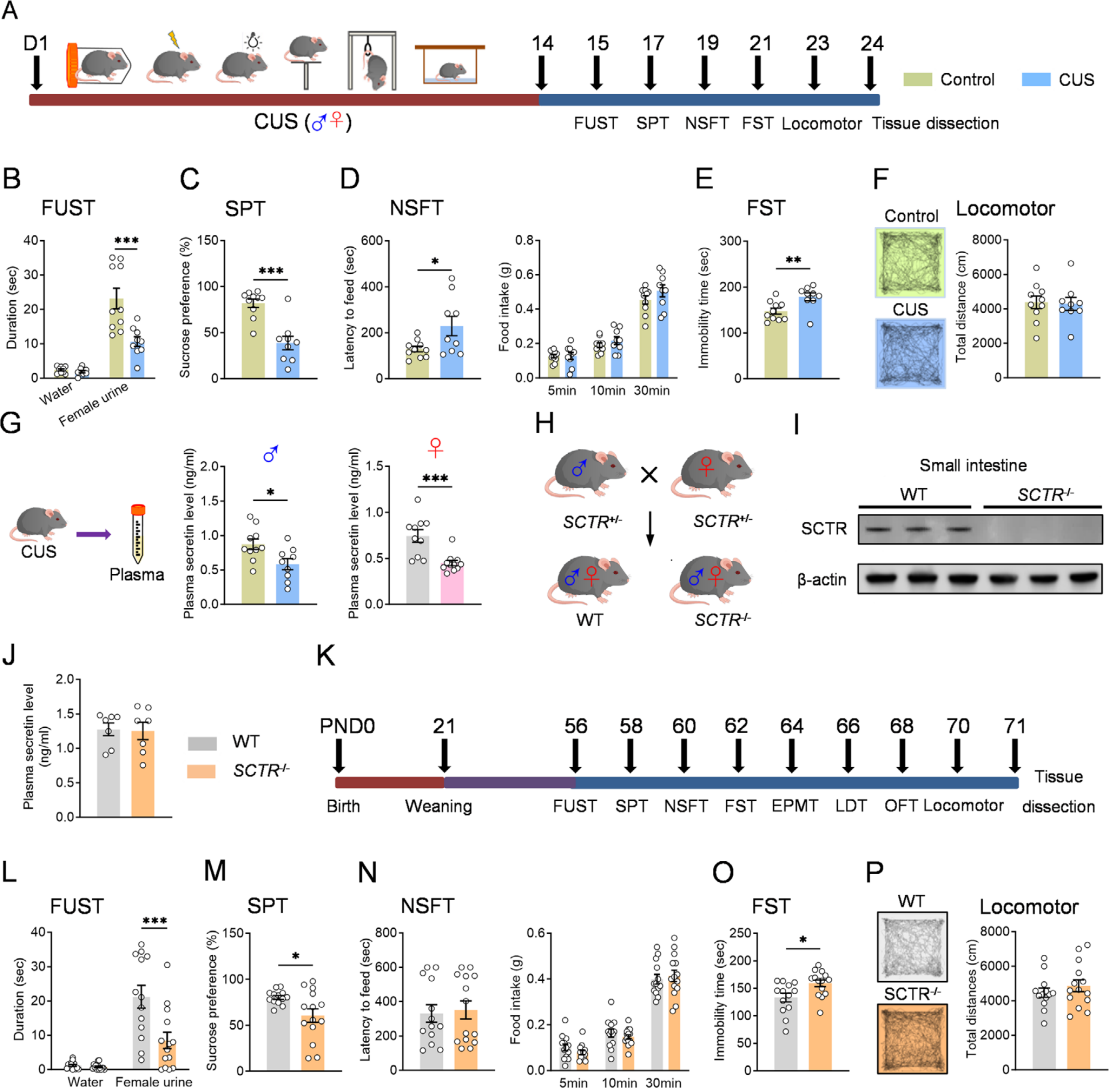
Downregulation of secretin in depression models and behavioral impairment following SCTR deletion. (A) Experimental design of mice subjected to CUS. D, day. (B) Statistical analysis of the sniffing time for female urine in FUST. (C) Statistical analysis of sucrose preference in the SPT. (D) Statistical analysis of latency to feed and food intake in the NSFT. (E) Statistical analysis of immobility time in the FST. (F) Representative activity tracking and total distance travelled during locomotor activity. (G) Schematic illustration of plasma collection from CUS mice and statistical analysis of plasma secretin levels. Male: n = 10 in the control group and n = 9 in the CUS group; female: n = 10 in the control group and n = 11 in the CUS group. (B, D right: two‐way ANOVA followed by Holm‐sidak's post‐hoc test; C, E, G right: Mann‐Whitney U test; D, left: two‐tailed unpaired t test with Welch's correction; F, G left: two‐tailed unpaired Student's t test). (H) Generation of global SCTR‐knockout mice. (I) Western blot analysis confirming the efficiency of gene knockout. (J) Statistical analysis of plasma secretin levels in SCTR^−/−^ and WT mice. n = 7 per group, Mann‐Whitney U test. (K) Experimental design of SCTR‐knockout mice. (L to P) Same as in (B to F), except that the data were from SCTR‐knockout mice. Male: n = 13 in the WT group and n = 14 in the *SCTR* ^−/−^ group. (L, N right: two‐way ANOVA followed by Holm‐sidak's post‐hoc test; M, two‐tailed unpaired t test with Welch's correction; N left: Mann‐Whitney U test; O, P: two‐tailed unpaired Student's t test). Data are presented as the means ± S.E.M. *p < 0.05, **p < 0.01, and ***p < 0.001.

To assess the potential role of secretin in depression, we measured plasma secretin levels in male and female mice subjected to CUS (Figure 1A). CUS‐exposed mice displayed robust depression‐like phenotypes, including reduced sniffing time for female urine in the female urine sniffing test (FUST; males), decreased sucrose preference in the sucrose preference test (SPT), increased latency to feed in the novelty‐suppressed feeding test (NSFT), and increased immobility time in the forced swim test (FST), without alterations in locomotor activity (Figures 1B–F; Figure S1A–D). Plasma secretin levels were significantly reduced in both male and female CUS‐exposed mice (Figure 1G).

We next examined whether disruption of secretin/SCTR signaling contributes to depression‐like behaviors. Global SCTR knockout (*SCTR* ^−/−^) mice were generated (Figure 1H; Figure S2A), and loss of SCTR protein expression was confirmed in the small intestine (Figure 1I), a tissue with high endogenous SCTR expression [33]. While, plasma secretin levels showed no significant differences between *SCTR* ^−/−^ and WT mice (Figure 1J). Behavioral analyses revealed that male *SCTR* ^−/−^ mice exhibited reduced sniffing time for female urine and decreased sucrose preference, whereas female mice showed no significant changes in these measures. Both male and female *SCTR* ^−/−^ mice displayed increased immobility time in FST, with no differences in NSFT and locomotor activity (Figure 1K–P; Figure S2B–E). Assessment of anxiety‐related behaviors, and interestingly, we found that there were no significant differences between *SCTR* ^−/−^ and control mice in the elevated plus maze test (EPMT), light‐dark box test (LDT), or open field test (OFT), with the exception of increased latency to enter the light compartment in male *SCTR* ^−/−^ mice (Figure S3A–F). Together, these results indicate that global loss of SCTR selectively induces depression‐like behaviors without broadly affecting anxiety‐related phenotypes.

### 2.2 Astrocyte‐Specific Deletion of SCTR in the DRN Induces Depression‐like Phenotypes

**FIGURE 2:**
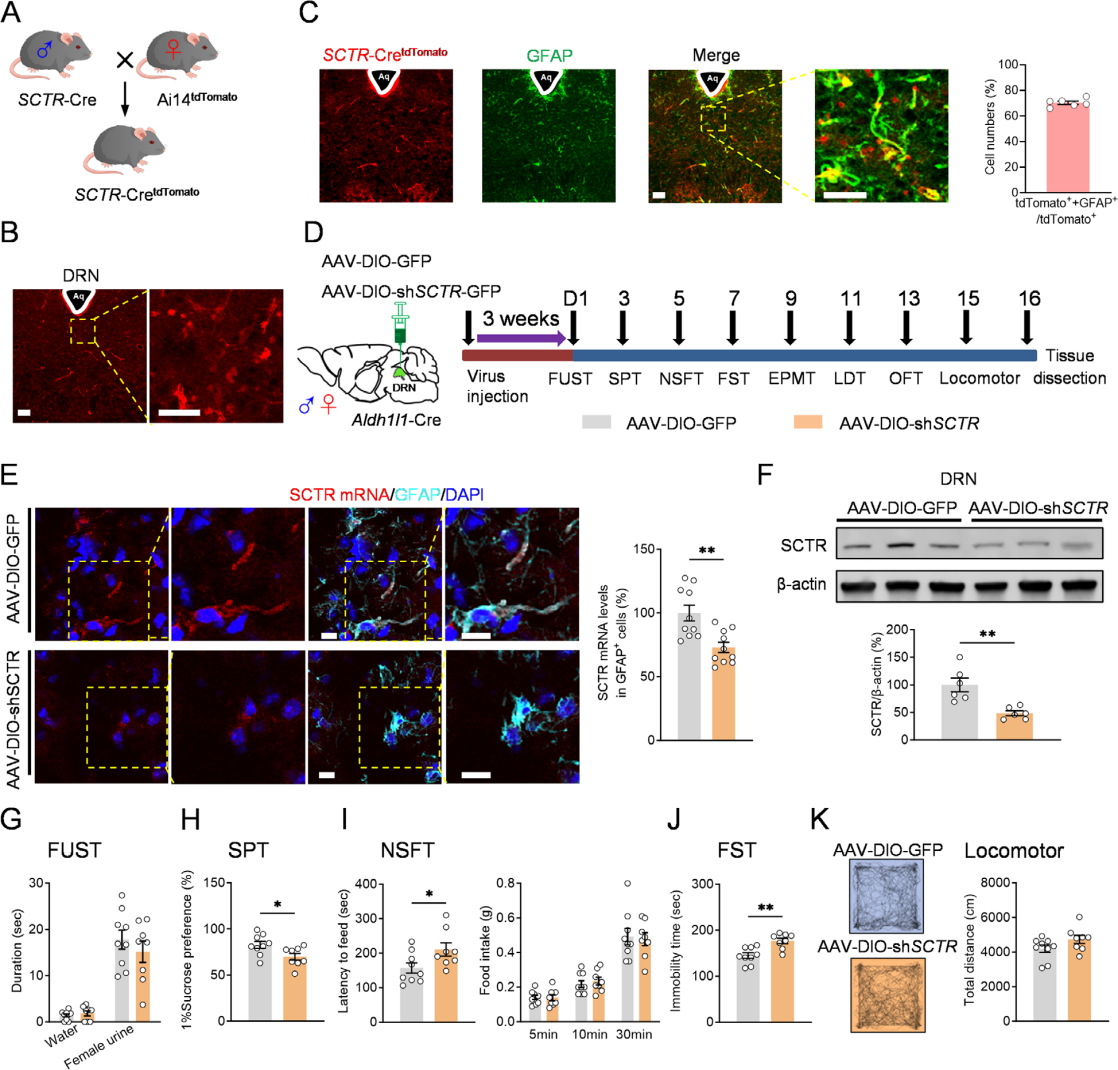
Astrocyte‐specific deletion of SCTR in the DRN induces depressive‐like behaviors in mice. (A) Generation of *SCTR*‐Cre^tdTomato^ reporter mice. (B) Representative coronal sections showing widespread tdTomato expression in the dorsal raphe nucleus. Scale bars = 100 µm (left), 50 µm (right). (C) Representative images showing colocalization of tdTomato (red) and GFAP (green) in the DRN. Scale bars = 100 µm (left), 50 µm (right). (n = 6 replicates from three mice). (D) Schematic representation of the experimental procedure for *Aldh1l1*‐Cre mice with injections of AAV‐DIO‐sh*SCTR*‐GFP or AAV‐DIO‐GFP. (E) Representative images of SCTR mRNA (red, in situ hybridization) and GFAP (cyan) co‐staining in the DRN of *Aldh1l1*‐Cre mice injected with AAV‐DIO‐sh*SCTR*‐GFP or AAV‐DIO‐GFP, quantification of SCTR mRNA expression in GFAP^+^ cells. Scale bars = 10 µm. (n = 10 replicates from five mice). (F) Western blotting images and quantification of SCTR in the DRN of *Aldh1l1*‐Cre mice injected with AAV‐DIO‐sh*SCTR*‐GFP or AAV‐DIO‐GFP. (n = 6 per group). (G) Statistical analysis of the sniffing time for female urine in the FUST. (H) Statistical analysis of sucrose preference in the SPT. (I) Statistical analysis of latency to feed and food intake in the NSFT. (J) Statistical analysis of immobility time in the FST. (K) Representative activity tracking and total distance travelled during locomotor activity. Male: AAV‐DIO‐GFP group, n = 9 mice; AAV‐DIO‐sh*SCTR*‐GFP group, n = 8 mice. (E, H, I left, J, K: two‐tailed unpaired Student's t test; F: two‐tailed unpaired t test with Welch's correction; G, I right: two‐way ANOVA followed by Holm‐sidak's post‐hoc test). Data are presented as the means ± S.E.M. *p < 0.05, **p < 0.01.

To examine the cellular distribution of SCTR in the brain, we generated *SCTR*‐Cre mice (Figure S4A) and crossed them with Ai14^tdTomato^ reporter mice to label SCTR‐expressing cells (Figure 2A). DRN, located beneath the midbrain aqueduct and projecting broadly to forebrain regions involved in emotional regulation [34, 35], contained abundant SCTR‐positive cells (Figure 2B). Immunohistochemical analyses revealed that SCTR‐labeled cells predominantly co‐localized with astrocyte (GFAP^+^) (Figure 2C), and were rarely detected in microglia (Iba1^+^) or neurons (NeuN^+^) (Figure S4B,C). No significant differences in SCTR protein levels in the DRN were observed between male and female wild‐type mice (Figure S5A).

To assess the functional role of astrocytic SCTR in depressive‐like behaviors, we injected AAV‐DIO‐sh*SCTR*‐GFP or control AAV‐DIO‐GFP into DRN of *Aldh1l1*‐Cre mice to achieve astrocyte‐specific SCTR knockdown mice (Figure 2D). SCTR mRNA expression was significantly reduced in GFAP‐positive astrocytes, and knockdown efficiency was further confirmed by decreased SCTR protein levels in the DRN (Figure 2E,F). Behavioral analyses showed that male mice with astrocytic SCTR knockdown exhibited reduced sucrose preference, increased latency to feed, and increased immobility time, while sniffing time for female urine remained unchanged (Figure 2G–J). In contrast, female mice showed no significant changes in sucrose preference or latency to feed, although immobility time was increased (Figure S5B–D). These behavioral alterations were not attributable to changes in locomotor activity (Figure 2K; Figure S5E). Assessment of anxiety‐related behaviors revealed no significant differences between astrocyte‐specific SCTR knockdown mice and controls (Figure S6A–F). Collectively, these findings demonstrate that selective ablation of SCTR in DRN astrocytes is sufficient to induce depression‐like phenotypes, with a more pronounced effect in male mice.

### 2.3 AMPKα1 in SCTR‐Expressed Astrocyte Regulates Depression‐ lLike Performance

**FIGURE 3:**
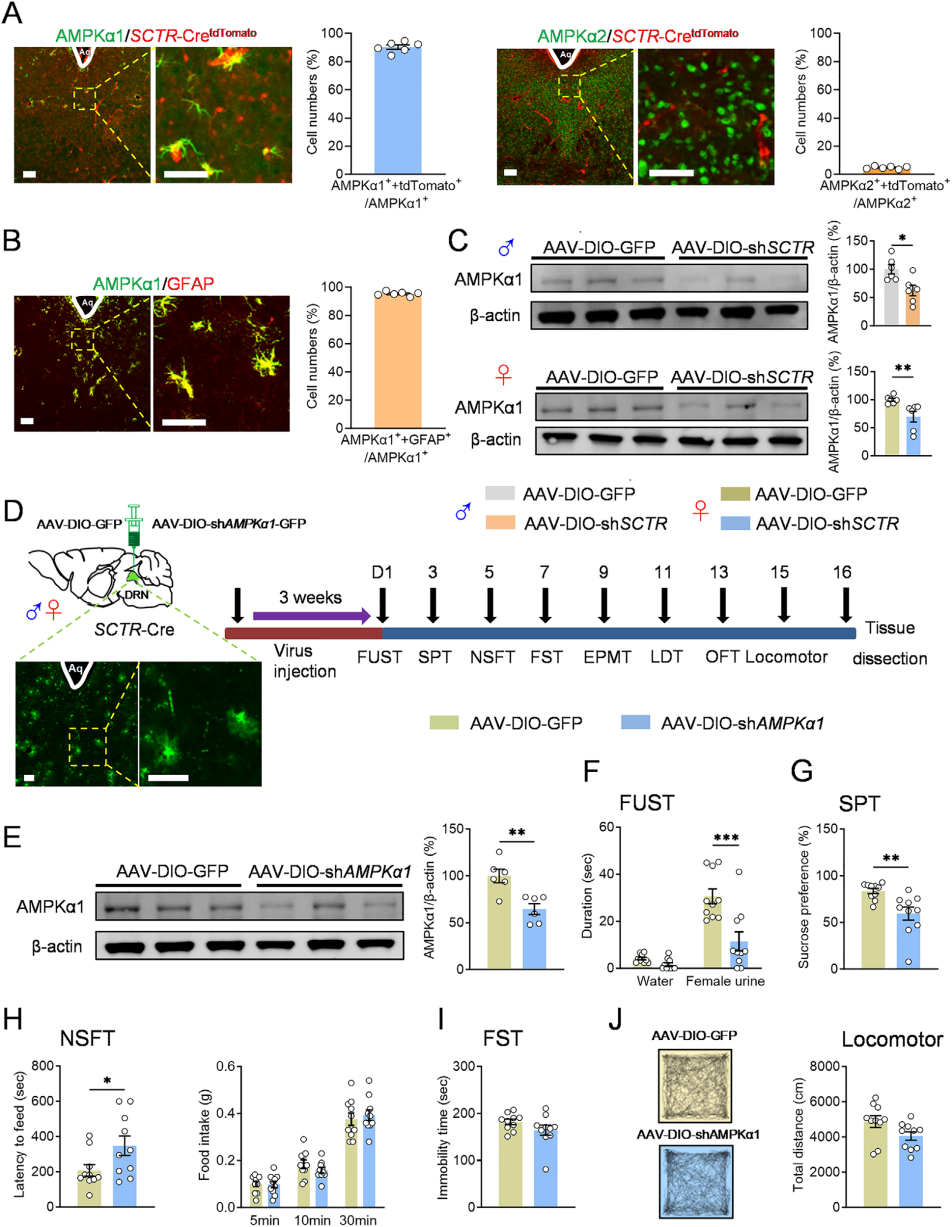
AMPKα1 in SCTR‐expressing astrocytes modulates depression‐like behaviors in mice. (A) Representative images showing colocalization of SCTR (red) with AMPKα1 (green) or AMPKα2 (green) in the DRN of *SCTR*‐Cre^tdTomato^ reporter mice. left section: AMPKα1; right section: AMPKα2. Scale bars = 100 µm (low magnification), 50 µm (high magnification). (n = 6 replicates from three mice). (B) Representative images showing colocalization of AMPKα1 (green) with GFAP (red) in the DRN. Scale bars = 100 µm (left), 50 µm (right). (n = 6 replicates from three mice). (C) Western blot images and quantification of AMPKα1 in the DRN of male and female *Aldh1l1*‐Cre mice injected with AAV‐DIO‐sh*SCTR*‐GFP or AAV‐DIO‐GFP. (n = 6 per group). (D) Upper panel, Schematic representation of the experimental procedure for *SCTR*‐Cre mice, with virus injection of AAV‐DIO‐sh*AMPKα1*‐GFP or AAV‐DIO‐GFP. Lower panel: Representative images of GFP expression in the DRN. Scale bars = 50 µm. (E) Western blot images (left) and quantification (right) of AMPKα1 in the DRN of *SCTR*‐Cre mice with virus injection of AAV‐DIO‐sh*AMPKα1*‐GFP or AAV‐DIO‐GFP. (n = 6 per group). (F) Statistical analysis of sniffing time for female urine in the FUST. (G) Statistical analysis of sucrose preference in the SPT. (H) Statistical analysis of the latency to feed and food intake in the NSFT. (I) Statistical analysis of immobility time in the FST. (J) Representative activity tracking and total distance travelled during locomotor activity. Male: n = 10 mice for the AAV‐DIO‐GFP group, n = 10 mice for the AAV‐DIO‐sh*AMPKα1*‐GFP group. (C upper, E, H left, I, J: two‐tailed unpaired Student's t test; C lower: Mann‐Whitney U test; F, H right: two‐way ANOVA followed by Holm‐sidak's post‐hoc test; G: two‐tailed unpaired t test with Welch's correction). Data are presented as the means ± S.E.M. *p < 0.05, **p < 0.01, and ***p < 0.001.

AMPKα signaling in the medial prefrontal cortex (mPFC) and hippocampus has been implicated in anxiety‐ and depression‐like behaviors [32, 36, 37]. We therefore examined whether AMPKα signaling in SCTR‐expressing astrocytes within the DRN contributes to depression‐related phenotypes. Immunostaining of DRN sections from *SCTR*‐Cre^tdTomato^ mice revealed that AMPKα1, but not AMPKα2, was highly co‐localized with tdTomato‐labeled SCTR‐expressing cells (Figure 3A). Further analysis showed that AMPKα1 expression was predominantly restricted to astrocyte (GFAP^+^) (Figure 3B) and was rarely detected in microglia (Iba1^+^), dopaminergic neurons (TH^+^), GABAergic neurons (GAD67^+^), or serotonergic neurons (5‐HT^+^) (Figure S7A–D). These observations prompted us to examine whether SCTR signaling regulates astrocytic AMPKα1 expression. Astrocyte‐specific knockdown of SCTR in the DRN resulted in a significant reduction in AMPKα1 protein levels in both sexes (Figure 3C). While, DRN AMPKα1 protein levels did not differ significantly by sex in wild‑type mice (Figure S8A).

To determine the functional relevance of AMPKα1 in SCTR‐expressing cells, we injected AAV‐DIO‐sh*AMPKα1*‐GFP or control AAV‐DIO‐GFP into DRN of *SCTR*‐Cre mice to selectively knockdown AMPKα1 (Figure 3D). Efficient viral‐mediated knockdown was confirmed by reduced AMPKα1 protein expression (Figure 3E). Behavioral analyses revealed that male mice with AMPKα1 knockdown in SCTR‐expressing cells exhibited reduced sniffing time for female urine in FUST, decreased sucrose preference in the SPT, increased latency to feed in NSFT, without changes in FST and locomotor activity (Figure 3F–J). Female AMPKα1 knockdown mice exhibited an increased latency to feed in the NSFT, no differences were observed in the SPT, FST, and locomotor activity (Figure S8B–E). Assessment of anxiety‐like behaviors showed that only male AMPKα1 knockdown mice displayed reduced open arm time and entries in the EPMT, whereas no differences were observed in the LDT, or OFT in either sex (Figure S9A–F). Together, these findings indicate that selective knockdown of AMPKα1 in SCTR‐expressing astrocytes within the DRN induces depressive‐like phenotypes, with more pronounced effects in male mice.

### 2.4 Astrocyte‐Derived Mt1 Regulates Serotonergic Neuronal AMPKα2 Activity

**FIGURE 4:**
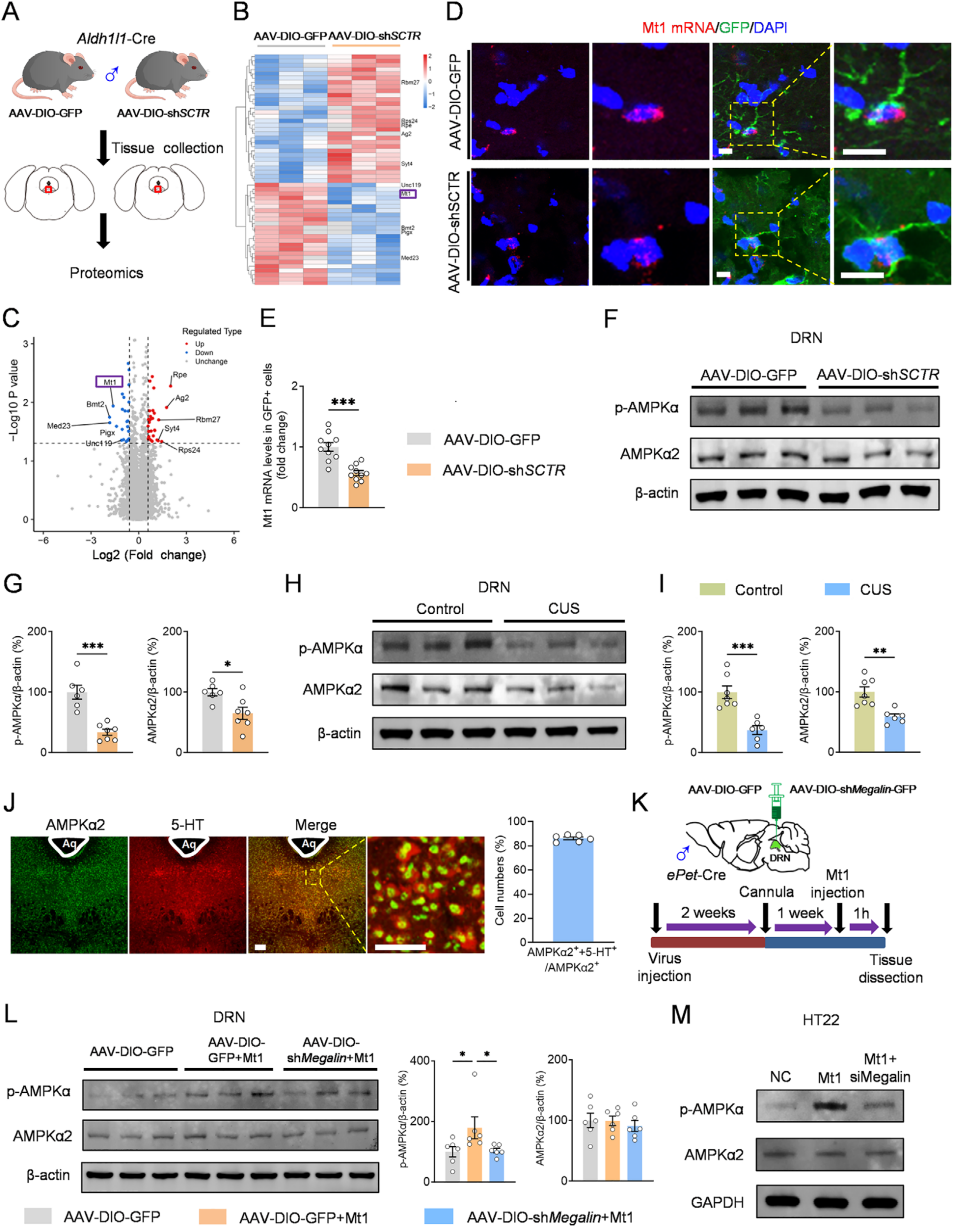
SCTR‐expressing astrocytes release Mt1 to modulate AMPKα2 activity in serotonergic neurons. (A) Schematic of proteomic analysis in the DRN of *Aldh1l1*‐Cre mice injected with AAV‐DIO‐sh*SCTR*‐GFP or AAV‐DIO‐GFP. (B and C) Heatmap (B) and volcano plots (C) of differentially expressed proteins in proteomic analysis. (D) Representative images showing GFP expression with Mt1 mRNA (red) in situ hybridization on DRN sections of *Aldh1l1*‐Cre mice injected with AAV‐DIO‐sh*SCTR*‐GFP or AAV‐DIO‐GFP. Scale bars = 10 µm. (E) Quantification of Mt1 mRNA expression in GFP + cells. (n = 10 replicates from five mice). (F and G) Western blot images (F) and quantification (G) of p‐AMPKα and AMPKα2 in the DRN of *Aldh1l1*‐Cre mice with virus injection of AAV‐DIO‐sh*SCTR*‐GFP or AAV‐DIO‐GFP. (AAV‐DIO‐GFP group, n = 6; AAV‐DIO‐sh*SCTR*‐GFP group, n = 7). (H and I) Western blot images (H) and quantification (I) of p‐AMPKα and AMPKα2 in the DRN of CUS mice and control mice. (n = 7 in the control group and n = 6 in the CUS group). (J) Representative images showing colocalization of AMPKα2 (green) with 5‐HT (red) in the DRN. Scale bars = 100 µm (left), 50 µm (right). (n = 6 replicates from three mice). (K) Schematic representation of the experimental procedure for *ePet*‐Cre mice, with virus injection of AAV‐DIO‐GFP or AAV‐DIO‐sh*Megalin*‐GFP, followed by Mt1 treatment. (L) Representative western blot images and quantitative analysis of p‐AMPK and AMPKα2 in the DRN of *ePet*‐Cre mice under Mt1 peptides treatment, or Mt1 treatment combined with megalin receptor knockdown. (n = 6 per group). (M) Western blot images of p‐AMPKα and AMPKα2 in HT22 cells treated with Mt1 peptides or Mt1 treatment combined with megalin receptor knockdown. (E, G, I: two‐tailed unpaired Student's t test; L left: Kruskal‐Wallis test followed by Dunn's post hoc test; L right: one‐way ANOVA followed by Sidak's multiple comparison test). Data are presented as the means ± S.E.M. *p < 0.05, **p < 0.01, and ***p < 0.001.

To investigate the downstream molecular mechanisms of SCTR signaling, we performed proteomic analysis of DRN tissue collected from male mice with astrocyte‐specific SCTR ablation and control mice (Figure 4A). Analysis focused on differentially expressed secreted proteins, among which metallothionein‐1 (Mt1) emerged as a prominent candidate (Figure 4B,C and Dataset S1). Mt1 is predominantly expressed in astrocytes and can be secreted into the extracellular space, where it acts on neuronal megalin receptor to promote axonal regeneration [38]. Consistent with this, further validation revealed a significant reduction in Mt1 mRNA expression, particularly within GFP‐labeled SCTR‐knockdown astrocytes (Figure 4D,E). To further investigate the effect of astrocytic SCTR on Mt1 secretion in vitro, we generated a stable SCTR‐knockdown C8‐D1A cell line using lentiviral transduction of SCTR shRNA (Figure S10A), and we found that SCTR knockdown markedly lowered Mt1 levels in cell culture supernatants (Figure S10B,C).

We next examined whether astrocyte‐specific SCTR disruption affects AMPKα2 signaling. Both p‐AMPKα and AMPKα2 protein levels were significantly reduced in the DRN of SCTR knockdown mice (Figure 4F,G). Similarly, mice subjected to CUS exhibited marked reductions in p‐AMPKα and AMPKα2 protein levels (Figure 4H,I). As SCTR and AMPKα2 were not co‐localized within the same cell populations, these findings suggested a non‐cell‐autonomous mechanism. Immunohistochemical mapping revealed that AMPKα2 was predominantly expressed in serotonergic neurons (5‐HT^+^), and was rarely detected in astrocytes (GFAP^+^), microglia (Iba1^+^), dopaminergic neurons (TH^+^), or GABAergic neurons (GAD67^+^) (Figure 4J; Figure S10D–G). To determine whether Mt1 modulates AMPKα2 signaling through megalin receptor on serotonergic neurons, AAV‐DIO‐GFP or AAV‐DIO‐sh*Megalin*‐GFP were injected into the DRN of ePet‐Cre mice. Following effective viral expression (Figure S10H), Mt1 peptides were microinfused into the same brain region (Figure 4K). Mt1 treatment significantly increased p‐AMPKα levels, an effect that was abolished by the selective knockdown of megalin receptor in serotonergic neurons (Figure 4L). Consistent with these findings, a similar effect was observed in HT22 cells (Figure 4M; Figure S10I). Collectively, these results indicate that SCTR‐expressing astrocytes release Mt1, which acts via neuronal megalin receptor to modulate AMPKα2 signaling in serotonergic neurons.

### 2.5 Serotonergic Neuron‐Specific AMPKα2 Knockout Induces Depression‐like Behaviors

**FIGURE 5:**
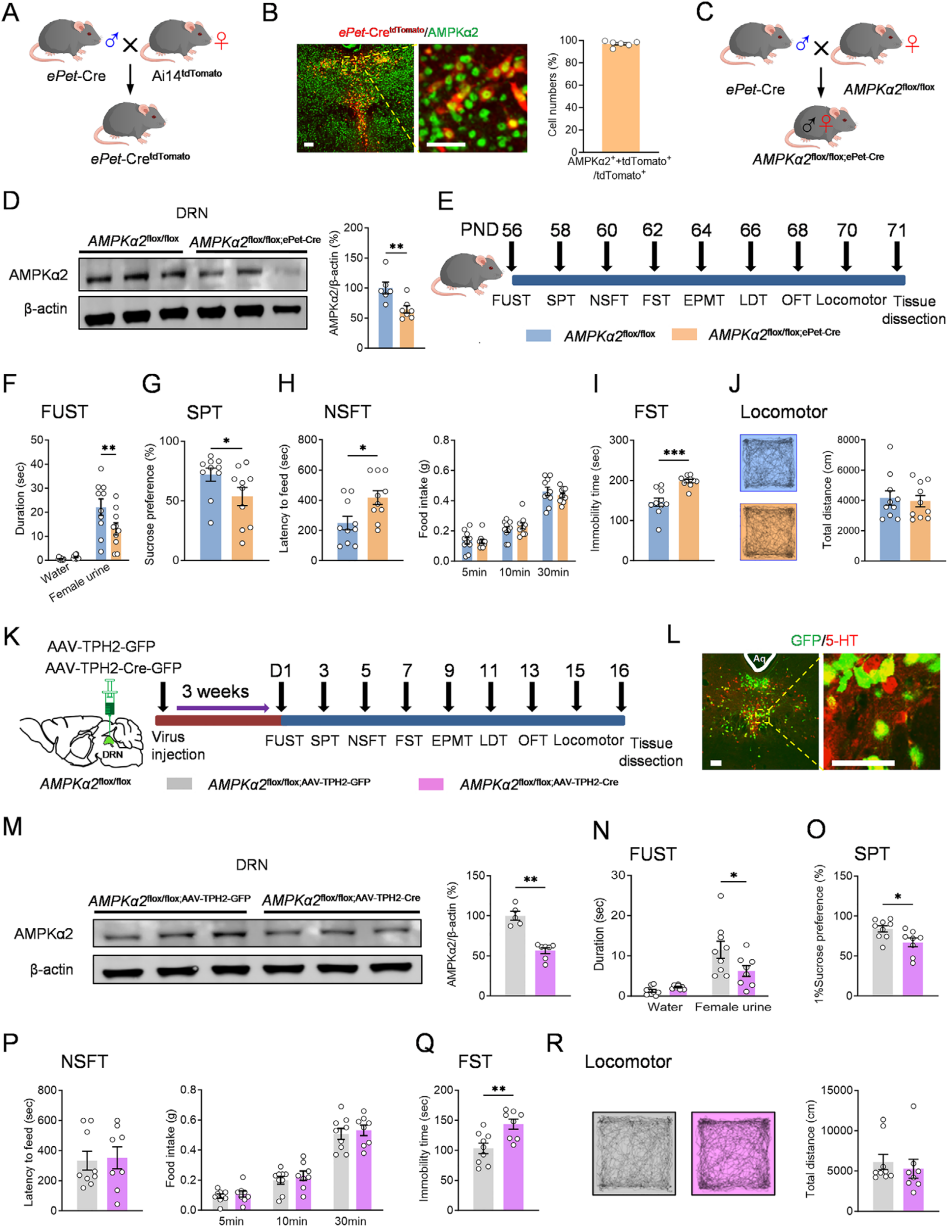
Specific deletion of AMPKα2 in serotonergic neurons induces depression‐like behaviors in mice. (A) Generation of *ePet*‐Cre^tdTomato^ reporter mice. (B) Representative images showing colocalization of tdTomato (red) and AMPKα2 (green) in the DRN. Scale bars = 100 µm (left), 50 µm (right). (n = 6 replicates from three mice). (C) Generation of *AMPKα2* ^flox/flox; ePet‐Cre^ mice. (D) Western blot images and quantification of AMPKα2 in the DRN of *AMPKα2* ^flox/flox^ mice and *AMPKα2* ^flox/flox; ePet‐Cre^ mice.(n = 6 for the *AMPKα2* ^flox/flox^ group; n = 7 for the *AMPKα2* ^flox/flox; ePet‐Cre^ group). (E) Experimental design for *AMPKα2* ^flox/flox; ePet‐Cre^ mice. (F) Statistical analysis of sniffing time for female urine in the FUST. (G) Statistical analysis of sucrose preference in SPT. (H) Statistical analysis of the latency to feed and food intake in the NSFT. (I) Statistical analysis of immobility time in the FST. (J) Representative activity tracking and total distance travelled during locomotor activity. male: n = 10 mice for the *AMPKα2* ^flox/flox^ group, n = 10 mice for the *AMPKα2* ^flox/flox; ePet‐Cre^ group. (D, H left, J: two‐tailed unpaired Student's t test; G: Mann‐Whitney U test; H right: two‐way ANOVA followed by Holm‐sidak's post‐hoc test; I: two‐tailed unpaired t test with Welch's correction). (K) Schematic representation of the experimental procedure for *AMPKα2* ^flox/flox^ mice, with virus injection of AAV‐TPH2‐Cre‐GFP or AAV‐TPH2‐GFP. (L) Representative images showing GFP expression and 5‐HT co‐staining (red) in the DRN. Scale bars = 100 µm (left), 50 µm (right). (M) Western blot images (left) and quantification (right) of AMPKα2 in the DRN of *AMPKα2* ^flox/flox^ mice with virus injection of AAV‐TPH2‐Cre‐GFP or AAV‐TPH2‐GFP. n = 5 for the AAV‐TPH2‐GFP group and n = 7 for the AAV‐TPH2‐Cre‐GFP group. (N to R) Same as in (F to J) except that the data were from *AMPKα2* ^flox/flox^ mice with virus injection of AAV‐TPH2‐Cre‐GFP or AAV‐TPH2‐GFP in DRN. male: n = 9 mice; AAV‐TPH2‐GFP group, n = 8 mice for the AAV‐TPH2‐Cre‐GFP group. (O, Q: two‐tailed unpaired Student's t test; M, P left, R: Mann‐Whitney U test; N, P right: two‐way ANOVA followed by Holm‐sidak's post‐hoc test). Data are presented as the means ± S.E.M. *p < 0.05, **p < 0.01, and ***p < 0.001.

To examine the effects of serotonergic neuron‐specific AMPKα2 loss on depression‐related behaviors, we generated *ePet*‐Cre^tdTomato^ reporter mice, in which serotonergic neurons are labeled with tdTomato fluorescence (Figure 5A) [13]. AMPKα2 was broadly co‐localized with tdTomato‐positive neurons in the DRN (Figure 5B). There were no significant sex differences in DRN AMPKα2 protein levels in wild‐type mice (Figure S11A). We then generated serotonergic neuron‐specific AMPKα2 conditional knockout (CKO) mice (*AMPKα2* ^flox/flox;ePet‐Cre^) by crossing the *AMPKα2* ^flox/flox^ mice with *ePet*‐Cre transgenic mice (Figure 5C). Efficient reduction of AMPKα2 protein expression was confirmed in these CKO mice (Figure 5D). Behavioral analyses revealed that male *AMPKα2* CKO mice exhibited robust depression‐like phenotypes (Figure 5E), including reduced sniffing time for female urine in FUST, decreased sucrose preference in the SPT, increased latency to feed in the NSFT, and increased immobility time in the FST, compared with wild‐type (WT) littermates (Figure 5F–I). In contrast, female CKO mice showed no significant differences in SPT or NSFT performance (Figure S11B,C), although immobility time in the FST was significantly increased (Figure S11D). Locomotor activity was unaffected in both sexes (Figure 5J; Figure S11E). Assessment of anxiety‐like behaviors revealed no differences between CKO and WT mice in EPMT, or OFT with the exception that female, but not male, CKO mice exhibited increased latency to enter the light compartment in the LDT (Figure S12A–F). These findings indicate that loss of AMPKα2 in serotonergic neurons induces depression‐like behaviors.

To assess the region‐specific contribution of DRN serotonergic neurons, we selectively knocked down AMPKα2 in DRN serotonergic neurons of *AMPKα2* ^flox/flox^ mice via stereotaxic injection of AAV‐TPH2‐Cre‐GFP or control AAV‐TPH2‐GFP (Figure 5K). Viral specificity was confirmed by co‐localization of GFP with 5‐HT immunoreactivity in DRN sections (Figure 5L), and efficient knockdown was verified by reduced AMPKα2 expression (Figure 5M). Male mice with DRN‐restricted AMPKα2 knockdown exhibited increased depression‐like behaviors in the FUST, SPT, and FST, without alterations in NSFT performance or locomotor activity (Figure 5N–R). In anxiety‐related assays, these mice showed reduced time in the light compartment in the LDT, with no changes in EPMT or OFT performance (Figure S13A–C). No significant behavioral differences were observed in female mice (Figure S14A–G).

To determine whether these effects were specific to serotonergic neurons, we next examined the consequences of AMPKα2 deletion in other neuronal populations within the DRN. Injection of AAV‐Vgat‐Cre‐GFP or control AAV‐Vgat‐GFP into the DRN of *AMPKα2* ^flox/flox^ mice was performed to achieve GABAergic neuron–specific deletion of AMPKα2 (Figure S15A). Specific expression of AAV‐Vgat‐Cre‐GFP and effective reduction of AMPKα2 protein levels were confirmed (Figure S15B–D). Male *AMPKα2* ^flox/flox;AAV‐Vgat‐Cre^ mice did not exhibit significant changes in depression‐like or anxiety‐like behaviors compared with control mice across all behavioral assays (Figure S15E–L). In contrast, female *AMPKα2* ^flox/flox;AAV‐Vgat‐Cre^ mice exhibited significantly reduced time spent in the open arms and fewer open arm entries in the EPMT, without alterations in behaviors assessed by the SPT, NSFT, FST, LDT, OFT, and locomotor activity (Figure S16A–G). We next injected AAV‐TH‐Cre‐GFP into the DRN of *AMPKα2* ^flox/flox^ mice to selectively delete AMPKα2 in dopaminergic neurons (Figure S17A). Efficient cell–type–specific AMPKα2 deletion was confirmed (Figure S17B–D). Neither male nor female *AMPKα2* ^flox/flox;AAV‐TH‐Cre^ mice showed significant differences in depression‐like or anxiety‐like behaviors compared with control mice (Figure S17E–L and S18A–G). Collectively, these results suggest that depression‐like phenotypes arising from AMPKα2 loss in the DRN are specific to serotonergic neurons.

### 2.6 Overexpression of AMPKα2 in DRN Serotonergic Neurons Produces Antidepressant‐like Activity

**FIGURE 6:**
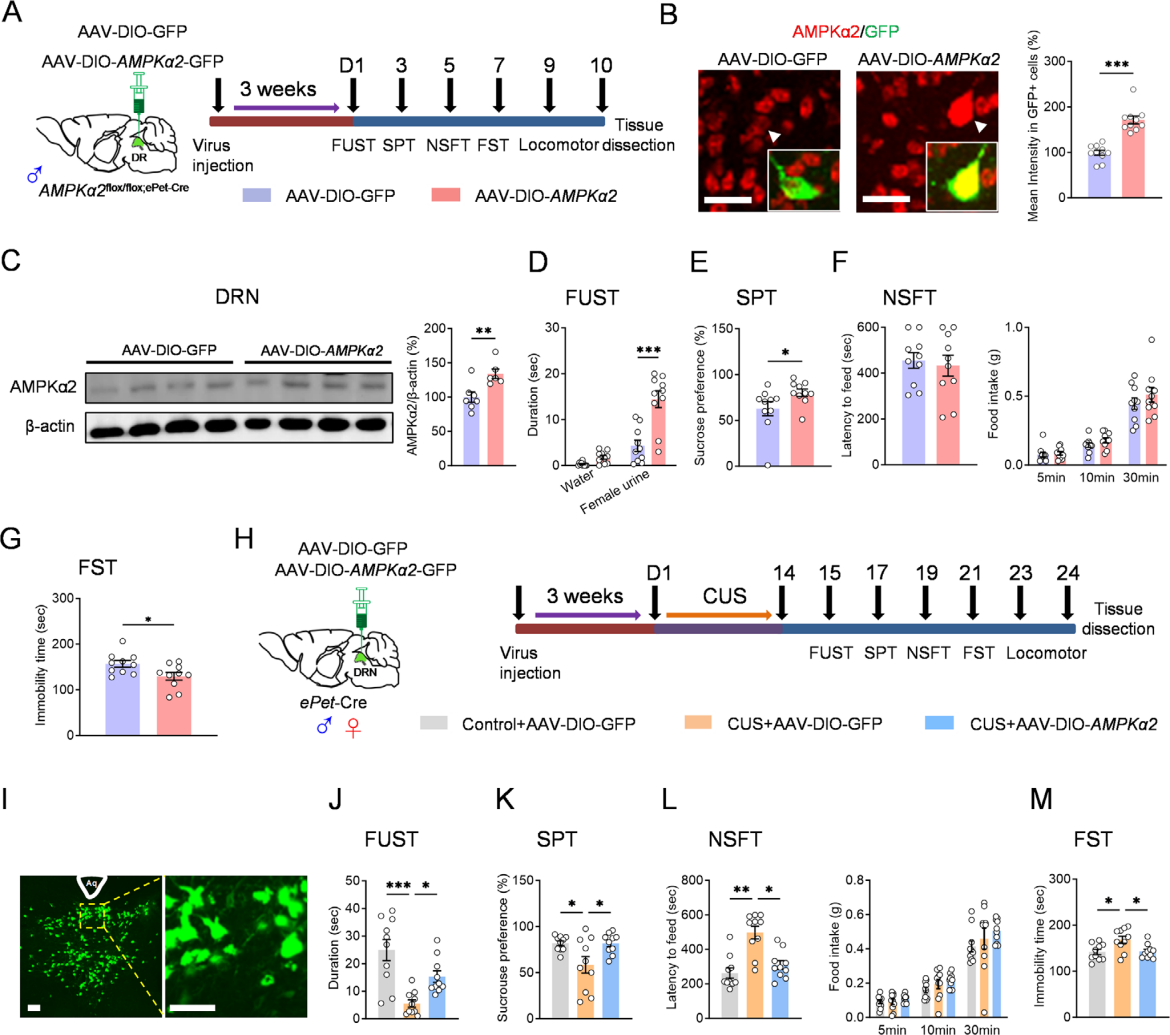
AMPKα2 overexpression in DRN 5‐HT neurons exerts antidepressant‐like effects. (A) Schematic representation of the experimental procedure for *AMPKα2* ^flox/flox; ePet‐Cre^ mice, with virus injection of AAV‐DIO‐*AMPKα2*‐GFP or AAV‐DIO‐GFP. (B) Representative images of GFP (green) and AMPKα2 (red) co‐staining in the DRN of *AMPKα2* ^flox/flox; ePet‐Cre^ mice injected with AAV‐DIO‐*AMPKα2*‐GFP or AAV‐DIO‐GFP. Quantification of AMPKα2 fluorescence intensity in GFP^+^ cells. Scale bars = 20 µm. (n = 10 replicates from five mice) (C) Western blot images and quantification of AMPKα2 in the DRN of *AMPKα2* ^flox/flox; ePet‐Cre^ mice with virus injection of AAV‐DIO‐*AMPKα2*‐GFP or AAV‐DIO‐GFP. n = 7 for the AAV‐DIO‐GFP group; n = 6 for the AAV‐DIO‐*AMPKα2*‐GFP group. (D) Statistical analysis of sniffing time for female urine in the FUST. (E) Statistical analysis of sucrose preference in the SPT. (F) Statistical analysis of latency to feed and food intake in the NSFT. (G) Statistical analysis of immobility time in the FST. Male: n = 10 mice for the AAV‐DIO‐GFP, n = 10 mice for the AAV‐DIO‐*AMPKα2*‐GFP group. (B, E: Mann‐Whitney U test; C, F left, G: two‐tailed unpaired Student's t‐test; D, F right: two‐way ANOVA followed by Holm‐sidak's post‐hoc test). (H) Schematic representation of the experimental procedure for *ePet*‐Cre mice, with virus injection of AAV‐DIO‐*AMPKα2*‐GFP or AAV‐DIO‐GFP. (I) Representative images of injection sites in the DRN. Scale bars = 100 µm (left), 50 µm (right). (J to M) Same as in (D to G), except that the data were from *ePet*‐Cre mice and represent the three experimental groups. Male: n = 10 mice for the Control+AAV‐DIO‐GFP, n = 10 mice for the CUS+AAV‐DIO‐GFP group, n = 10 mice for the CUS+AAV‐DIO‐*AMPKα2* group. (J, K, M: one‐way ANOVA followed by Sidak's multiple comparison test; L left: Kruskal‐Wallis test followed by Dunn's post hoc test; L right: two‐way ANOVA followed by Homl‐sidak's post‐hoc test). Data are presented as the means ± S.E.M. *p < 0.05, **p < 0.01, and ***p < 0.001.

To determine whether restoring AMPKα2 expression in serotonergic neurons is sufficient to reverse the depression‐like behaviors caused by AMPKα2 loss, we injected AAV‐DIO‐*AMPKα2*‐GFP or control virus into the DRN of male serotonergic neuron‐specific AMPKα2 CKO mice (Figure 6A). Immunostaining and western blot analyses confirmed a significant increase in AMPKα2 protein levels in the DRN of serotonergic neuron‐specific AMPKα2 CKO mice (Figure 6B,C). AMPKα2 overexpression significantly increased sniffing time for female urine in the FUST, enhanced sucrose preference in the SPT, and reduced immobility time in the FST, compared with control virus–injected mice. No significant differences were observed in the NSFT or locomotor activity (Figure 6D–G; Figure S19A).

We next examined whether viral‐mediated AMPKα2 overexpression in DRN serotonergic neurons could ameliorate depression‐like behaviors induced by CUS. AAV‐DIO‐*AMPKα2*‐GFP was injected into the DRN of *ePet*‐Cre mice to selectively overexpress AMPKα2 in serotonergic neurons before CUS exposure (Figure 6H,I). Behavioral analyses revealed that AMPKα2 overexpression normalized depression‐like behaviors in the FUST, SPT, NSFT, and FST following CUS, without affecting locomotor activity, compared with control virus–injected mice (Figure 6J–M, Figure S19B). In contrast, female mice did not exhibit significant rescue effects in any of the depression‐related behavioral assays, including the SPT, NSFT, FST, or locomotor activity (Figure S20A–D). Together, these findings demonstrate that AMPKα2 overexpression in DRN serotonergic neurons is sufficient to ameliorate depression‐like behaviors in male mice exposed to CUS.

### 2.7 Foxc2 Rescues Behavioral and Neural Activity Deficits in AMPKα2‐deficient Serotonergic Neurons

**FIGURE 7:**
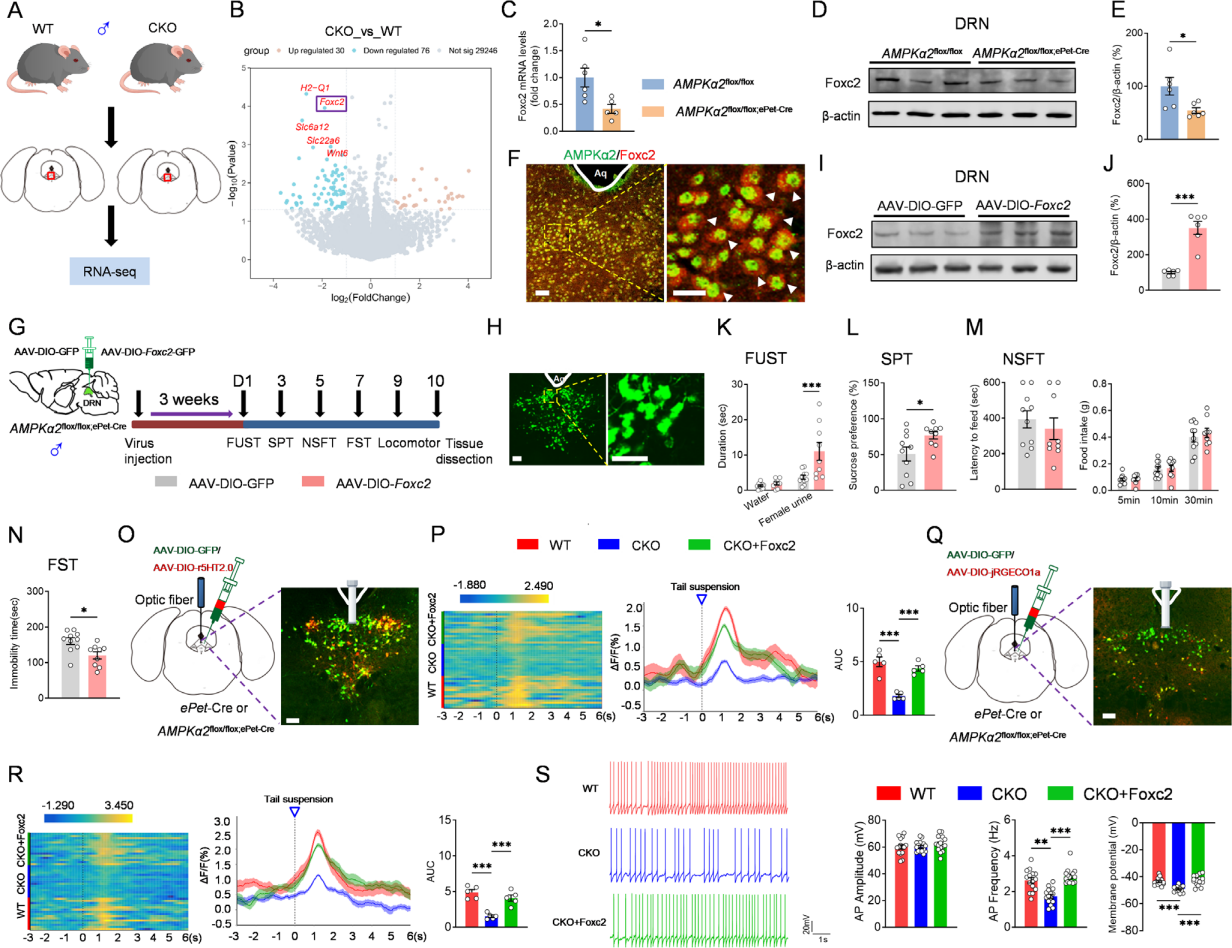
Foxc2 restores behavior and synaptic function in AMPKα2‐impaired serotonergic neurons. (A) Schematic of the RNA sequencing analysis in the DRN of *AMPKα2* CKO mice and WT mice. CKO mice: *AMPKα2* ^flox/flox; ePet‐Cre^ mice; WT mice: *AMPKα2* ^flox/flox^ mice. (B) Volcano plot of differentially expressed genes in RNA sequencing analysis. (C) qPCR analysis of Foxc2 mRNA levels in the DRN of *AMPKα2* ^flox/flox; ePet‐Cre^ mice and *AMPKα2* ^flox/flox^ mice. n = 6 for the *AMPKα2* ^flox/flox^ group; n = 5 for the *AMPKα2* ^flox/flox; ePet‐Cre^ group. (C: two‐tailed unpaired Student's t‐test). (D and E) Western blot images (D) and quantification (E) of Foxc2 in the DRN of *AMPKα2* ^flox/flox; ePet‐Cre^ mice and *AMPKα2* ^flox/flox^ mice. n = 6 for the *AMPKα2* ^flox/flox^ group; n = 6 for the *AMPKα2* ^flox/flox; ePet‐Cre^ group. (E: two‐tailed unpaired t test with Welch's correction). (F) Representative images showing colocalization of AMPKα2 (green) with Foxc2 (red) in the DRN (marked by white triangles). Scale bars = 50 µm (left), 20 µm (right). (G) Schematic representation of the experimental procedure for *AMPKα2* ^flox/flox; ePet‐Cre^ mice, with virus injection of AAV‐DIO‐*Foxc2*‐GFP or AAV‐DIO‐GFP. (H) Representative images showing GFP expression in the DRN. Scale bars = 100 µm (left), 50 µm (right). (I and J) Western blot images (I) and quantification (J) of Foxc2 in the DRN of *AMPKα2* ^flox/flox; ePet‐Cre^ mice with virus injection of AAV‐DIO‐*Foxc2*‐GFP or AAV‐DIO‐GFP. n = 6 for the AAV‐DIO‐GFP group; n = 6 for the AAV‐DIO‐*Foxc2*‐GFP group. (J: two‐tailed unpaired t test with Welch's correction). (K) Statistical analysis of sniffing time for female urine in FUST. (L) Statistical analysis of sucrose preference in SPT. (M) Statistical analysis of the latency to feed and food intake in the NSFT. (N) Statistical analysis of immobility time in the FST. n = 10 for the AAV‐DIO‐GFP group and n = 9 mice for the AAV‐DIO‐*Foxc2*‐GFP group. (K, M right: two‐way ANOVA followed by Holm‐sidak's post‐hoc test; L, N: two‐tailed unpaired Student's t‐test; M left: Mann‐Whitney U test). (O) Schematic of the viral strategy for monitoring serotonin release in the DRN, representative image showing virus expression, and the optical fiber implantation site. Scale bars = 100 µm. (P) Representative heatmaps, peri‐event plots of ΔF/F, and AUC showing serotonin transients. n = 15 trials with five mice per group. (P right: one‐way ANOVA followed by Sidak's multiple comparison test). (Q) Schematic illustration of the viral strategy for recording calcium signals in the DRN, representative image showing virus expression and optical fiber implantation site. Scale bars = 100 µm. (R) Representative heatmaps, peri‐event plots of ΔF/F, and AUC showing calcium dynamics. n = 15 trials with five mice per group. (R right: one‐way ANOVA followed by Sidak's multiple comparison test). (S) Representative traces of action potentials of serotonin neurons, and quantitative analysis of AP amplitude, AP frequency, and membrane potential. n = 15 neurons from 5 mice per group across three groups (group (WT): *ePet*‐cre+AAV‐DIO‐GFP, group (CKO): *AMPKα2* ^flox/flox; ePet‐Cre^+ AAV‐DIO‐GFP, group (CKO+Foxc2): *AMPKα2* ^flox/flox; ePet‐Cre^+ AAV‐DIO‐*Foxc2*‐GFP). (S left, right: one‐way ANOVA followed by Sidak's multiple comparison test; S middle: Kruskal‐Wallis test followed by Dunn's post hoc test). Data are presented as the means ± S.E.M. *p < 0.05, **p < 0.01, and ***p < 0.001.

To identify downstream genes mediating AMPKα2‐dependent regulation of depression‐like behaviors in DRN serotonergic neurons, we performed RNA sequencing on DRN tissue from serotonergic neuron‐specific *AMPKα2* CKO mice and WT littermates (Figure 7A). This analysis identified 76 downregulated and 30 upregulated genes exhibiting at least a 1.0‐fold change in expression (Figure 7B; Dataset S2). Among the top five significantly downregulated genes (H2‐Q1, Foxc2, Slc6a12, Wnt6, Slc22a6), we focused on the transcription factor Foxc2 as a potential downstream effector of AMPKα2. Reduced Foxc2 expression was confirmed in the DRN of serotonergic neuron‐specific *AMPKα2* CKO mice (Figure 7C–E), and AMPKα2 was predominantly co‐localized with Foxc2 in the DRN (Figure 7F). To determine whether Foxc2 functions downstream of AMPKα2 to regulate depression‐like behaviors, we injected AAV‐DIO‐*Foxc2*‐GFP or control AAV‐DIO‐GFP into the DRN of male serotonergic neuron‐specific *AMPKα2* CKO mice (Figure 7G–J). Foxc2 overexpression normalized depression‐like behaviors compared with control virus–treated CKO mice, without affecting performance in NSFT and locomotor activity (Figure 7K–N; Figure S21). These findings indicate that Foxc2 overexpression is sufficient to rescue depression‐like behaviors in male AMPKα2‐deficient mice.

We next examined whether Foxc2 overexpression restores serotonergic neuron functions. AAV‐DIO‐*Foxc2*‐GFP or control virus was co‐injected with the genetically encoded serotonin sensor AAV‐DIO‐r5HT2.0 into the DRN of *AMPKα2* ^flox/flox; ePet‐Cre^ mice (Figure 7O). Fiber photometry recordings revealed that Foxc2 overexpression rescued the reduced serotonin release observed in AMPKα2‐deficient serotonergic neurons (Figure 7P). To assess neuronal activity, we injected AAV‐DIO‐*Foxc2*‐GFP or control virus together with AAV‐DIO‐jRGECO1a into the DRN of *AMPKα2* ^flox/flox; ePet‐Cre^ mice (Figure 7Q). Fiber photometry showed that Foxc2 overexpression restored attenuated serotonergic neuronal activity (Figure 7R). Consistently, whole‐cell patch‐clamp recordings demonstrated that Foxc2 overexpression rescued reduced spontaneous firing and action potential frequency, and normalized hyperpolarized membrane potential in AMPKα2‐deficient serotonergic neurons (Figure 7S). Together, these data indicate that Foxc2 overexpression restores serotonergic neurotransmitter and neuronal activity deficits caused by AMPKα2 loss.

Finally, we assessed whether Foxc2 overexpression in DRN serotonergic neurons ameliorates depression‐like behaviors induced by CUS. AAV‐DIO‐*Foxc2*‐GFP or control virus was injected into the DRN of *ePet*‐Cre mice to drive Foxc2 expression in serotonergic neurons prior to CUS exposure (Figure S22A,B). Foxc2 overexpression rescued depression‐like behaviors following CUS in male mice (Figure S22C–E), without significant effects on FST performance or locomotor activity (Figure S22F,G). No significant rescue effects were observed in female mice (Figure S23A–D).

To further examine the behavioral consequences of Foxc2 loss in DRN serotonergic neurons, we stereotaxically injected AAV‐DIO‐sh*Foxc2*‐GFP or control AAV‐DIO‐GFP into the DRN of *ePet*‐Cre mice to generate serotonergic neuron‐specific Foxc2 knockdown mice (Figure S24A–C). Behavioral analyses revealed that male mice with serotonergic Foxc2 knockdown exhibited depression‐like behaviors, including reduced sniffing time in the FUST, decreased SPT, and increased immobility time in the FST, whereas performance in NSFT was unaffected (Figure S24D–G). Assessment of anxiety‐like behaviors showed no significant differences in the EPMT, LDT, OFT, and locomotor activity compared with control mice (Figure S24H–K). In female mice, serotonergic Foxc2 knockdown did not produce significant changes in depression‐like behaviors across the SPT, NSFT, and FST (Figure S25A–C). However, female knockdown mice showed fewer open arm entries in the EPMT, with no differences observed in the LDT or OFT (Figure S25D–F). Locomotor activity remained unchanged (Figure S25G). Together with the rescue experiments, these findings indicate that Foxc2 in DRN serotonergic neurons bidirectionally modulates depression‐like behaviors in a sex‐dependent manner.

To explore potential downstream pathways regulated by Foxc2, we performed RNA sequencing analysis on DRN tissue from serotonergic neuron‐specific Foxc2 knockdown mice. This analysis identified 469 downregulated and 2756 upregulated genes exhibiting at least a 1.0‐fold change in expression (Figure S6A and Dataset S3). Gene Ontology (GO) enrichment analysis revealed significant associations with neurotransmitter transport, secretory processes, and receptor activity (Figure S26B), while Kyoto Encyclopedia of Genes and Genomes (KEGG) pathway analysis showed significant enrichment of serotonergic synapse‐related pathways (Figure S26C). These data suggest that Foxc2 in DRN serotonergic neurons contribute to regulation of serotonergic system function.

### 2.8 AMPKα2 Suppresses Foxc2 Transcription via KAT7 Mediated Histone H4 Acetylation

**FIGURE 8:**
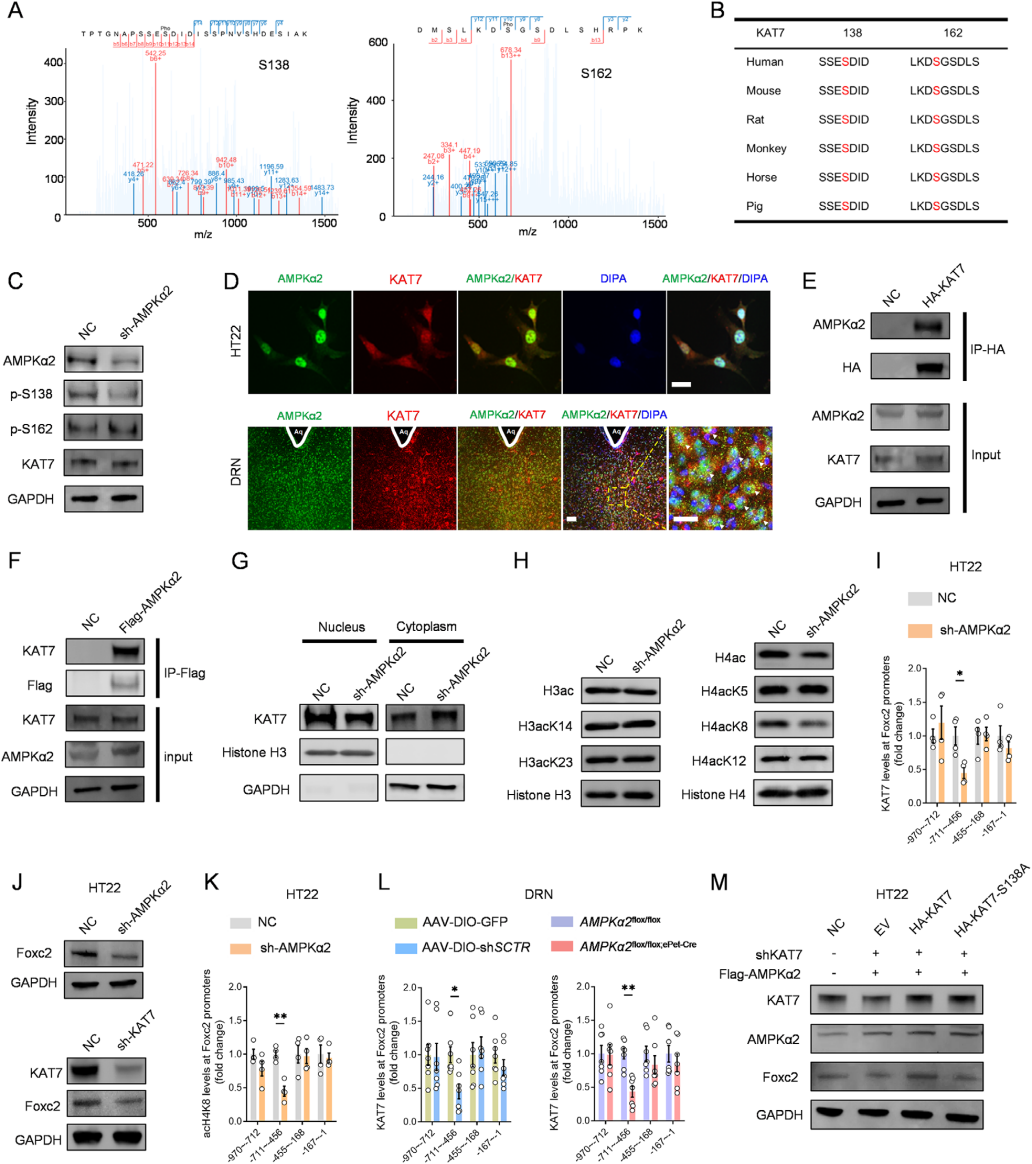
AMPKα2 deficiency inhibits KAT7 nucleus translocation and Foxc2 transcription via hypoacetylation. (A) Ser138 and Ser162 phosphorylation modifications of KAT7 peptides identified by LC‐MS. (B) Sequence conservation analysis of the relevant amino acids in KAT7. (C) Western blot analysis of cell lysates, as indicated. (D) Upper panel: Immunofluorescence assay of the co‐localization of endogenous AMPKα2 (green) and KAT7 (red) in HT22 cells. Scale bars = 20 µm. Lower panel: Representative images showing colocalization of AMPKα2 (green) with KAT7 (red) in the DRN. Scale bars = 50 µm (left), 20 µm (right). (E and F) Immunoblot analysis of whole‐cell lysates (input) and anti‐HA(E), anti‐Flag (F) immunoprecipitates derived from HT22 cells transfected with HA‐KAT7 or Flag‐AMPKα2. (G) Representative western blot of KAT7 expression in the nucleus and cytoplasm of HT22 cells after AMPKα2 knockdown. (H) Western blot of H3ac(K14,K23) or H4ac(K5,K8,K12) in HT22 cells following AMPKα2 knockdown. (I) Binding levels of KAT7 at Foxc2 promoters in HT22 cells following AMPKα2 knockdown. n = 4 per group. (J) Representative western blot analysis of cell lysates following AMPKα2 or KAT7 knockdown. (K) Binding levels of acH4K8 at Foxc2 promoters in HT22 cells following AMPKα2 knockdown. n = 4 per group. (L) The binding levels of KAT7 at Foxc2 promoters in DRN with either astrocyte‐specific SCTR ablation or serotonergic neuron‐specific AMPKα2 knockout. n = 7 per group. (M) KAT7 knockdown HT22 cells were co‐transfected with indicated Flag‐AMPKα2 and either HA‐KAT7 or HA‐KAT7‐S138A. Foxc2 expression was confirmed by western blotting. (I, K, L: two‐way ANOVA followed by Holm‐sidak's post‐hoc test). Data are presented as the means ± S.E.M. *p < 0.05, **p < 0.01.

To investigate how AMPKα2 regulates Foxc2 expression, we generated a stable AMPKα2 knockdown HT22 cell line using lentiviral delivery of AMPKα2 shRNA. Phosphoproteomic analysis identified the acetyltransferase KAT7 as a candidate downstream effector, exhibiting reduced phosphorylation at Ser138 and Ser162 following AMPKα2 knockdown (Figure 8A; Dataset S4). Both phosphorylation sites were predicted as high‐confidence AMPKα2 targets using GPS 6.0 software [39, 40] (Figure S27A) and were highly conserved across species (Figure 8B). Consistent with these predictions, AMPKα2 knockdown significantly reduced phosphorylation of KAT7 at Ser138, whereas phosphorylation at Ser162 and total KAT7 protein levels were unchanged (Figure 8C). Immunofluorescence analysis revealed robust co‐localization of AMPKα2 and KAT7 in both the DRN and HT22 cells (Figure 8D).

Co‐immunoprecipitation assays further demonstrated interaction between endogenous AMPKα2 and exogenously expressed HA‐KAT7 (Figure 8E), as well as between endogenous KAT7 and FLAG‐tagged AMPKα2 (Figure 8F). AMPKα2 knockdown reduced KAT7 nucleus localization of KAT7, accompanied by increased cytoplasmic accumulation (Figure 8G).

KAT7 possesses acetyltransferase activity toward histone H3 (K14, K23) and histone H4 (K5, K8, K12) and plays roles in various biological processes [41, 42, 43]. We therefore investigated the effects of AMPKα2 knockdown on histone acetylation and found that it significantly reduced global histone H4 acetylation, with a marked decrease in H4K8 acetylation, whereas histone H3 acetylation levels remained unchanged (Figure 8H). The histone acetyltransferase activity of KAT7 is dependent on its association with scaffold proteins, such as Jade or BRPF, through which it forms functional KAT7‐containing complexes [44, 45]. Accordingly, we transfected Flag‐KAT7 plasmids into HT22 cells and observed that endogenous Jade1 co‐immunoprecipitated with the exogenously expressed KAT7 (Figure S27B).

To determine the impact of AMPKα2 depletion on KAT7‐mediated epigenetic regulation, we analyzed KAT7 recruitment to the promoter regions of the top five significantly downregulated genes following AMPKα2 knockdown. Chromatin immunoprecipitation revealed markedly decreased occupancy of KAT7 at the Foxc2 promoter region (−711 to −456), but not at the H2‐Q1, Slc6a12, Wnt6, Slc22a6 promoters (Figure 8I; Figure S27C–F). AMPKα2 knockdown significantly decreased Foxc2 expression; Lentiviral‐mediated KAT7 knockdown similarly reduced Foxc2 expression, indicating that KAT7 positively regulates Foxc2 transcription (Figure 8J). ChIP assays also demonstrated a significant decrease in H4K8ac enrichment at the Foxc2 promoter (−711 to −456) following AMPKα2 knockdown (Figure 8K). We next examined KAT7 recruitment at the same Foxc2 promoter region in the DRN of male mice with either astrocyte‐specific SCTR ablation or serotonergic neuron‐specific AMPKα2 knockout and found that KAT7 occupancy was significantly reduced (Figure 8L).

To identify the specific serine residue of KAT7 regulated by AMPKα2, we performed site‐directed mutagenesis in which serine 138 of KAT7 (S138) was substituted with alanine (A) to mimic the non‐phosphorylated state. KAT7‐depleted HT22 cells were co‐transfected with Flag‐AMPKα2 and either HA‐KAT7 or HA‐KAT7‐S138A. These results showed that KAT7 depletion reduced Foxc2 expression even in the presence of AMPKα2 co‐overexpression. While overexpression of WT KAT7 restored Foxc2 expression, the KAT7 S138A mutant failed to rescue the suppressed Foxc2 levels (Figure 8M). These data indicate that the S138 residue of KAT7 serves as a key phosphorylation target of AMPKα2. Collectively, these results demonstrate that downregulation of AMPKα2 disrupts KAT7 nucleus translocation and leads to reduced histone acetylation, ultimately suppressing Foxc2 transcription.

## 3 Discussion

The present study reveals a pivotal role for SCTR signaling in the DRN in regulating the onset of depression. We identify AMPKα1 as a key downstream effector of SCTR signaling and demonstrate its involvement in suppressing Mt1 exocytosis from astrocytes. Reduced Mt1 impairs megalin‐mediated downstream signaling of AMPKα2 in serotonergic neurons. Moreover, gain or loss of AMPKα2 in DRN serotonergic neurons bidirectionally regulates depression‐like behaviors and leads to the identification of the transcription factor Foxc2 as a key downstream target. Mechanistically, downregulation of AMPKα2 disrupts KAT7 nucleus translocation and decreases histone acetylation, ultimately suppressing Foxc2 transcription. This downregulation is associated with impaired serotonin release and reduced neuronal excitability in DRN serotonergic neurons.

Secretin/SCTR signaling has been implicated in mood‐related disorders within the CNS [46]. Previous studies showed that SCTR‐deficient mice exhibit impaired social interaction [22], intracerebroventricular administration of secretin produces anxiolytic and fear‐reducing effects [23]. However, the involvement of SCTR signaling in the pathogenesis of depression has remained unclear. Astrocytes, the most abundant glial cell type in the brain, play a crucial role in maintaining CNS homeostasis [47, 48]. Emerging evidence suggests that astrocytes may sense circulating stress‐related hormonal fluctuations before neurons [49, 50]. In the present study, we found that SCTR is predominantly expressed in DRN astrocytes, and selective knockdown of SCTR in these astrocytes induces depressive‐like behaviors. Given the complexity of depressive disorders [51, 52, 53], regulation of SCTR signaling in DRN astrocytes warrants further investigation to elucidate disease etiology.

AMPK serves as a central cellular energy sensor and has been increasingly implicated in neuroprotection within the CNS [30]. Reduced p‐AMPKα levels have been reported in the hippocampus of murine depression models [29, 54], and several antidepressant interventions normalize these alterations [31, 32, 55]. However, the role of AMPKα signaling within the DRN has remained unclear. Our results show that AMPKα1 is predominantly expressed in astrocytes, whereas AMPKα2 is primarily localized in serotonergic neurons. Cell‐type‐specific knockdown of AMPKα1 in SCTR‐expressing astrocytes induces depressive‐like behaviors, suggesting that AMPKα1 acts as a downstream effector of SCTR signaling. Furthermore, p‐AMPKα and AMPKα2 levels were significantly reduced following astrocyte‐specific SCTR knockdown. Because AMPKα2 and SCTR are expressed in different cell populations, their functional interaction likely occurs through non‐cell‐autonomous mechanisms. Proteomic analysis and in situ hybridization revealed that Mt1 expression was reduced in astrocytes following cell‐specific SCTR knockdown. Previous study showed that Mt1 is predominantly expressed in astrocytes and can be secreted into the extracellular milieu, enabling interaction with neuronal megalin receptor to promote axonal regeneration [38]. Megalin is highly expressed in neurons but minimally in astrocytes, and its overexpression alleviates depressive‐like behaviors caused by Mettl3 depletion in the hippocampus [56]. Several studies have reported activation of ERK, CREB, and AKT signaling in neurons following Mt1 peptide stimulation [57, 58]. Consistently, Mt1 peptide treatment increased p‐AMPKα levels in HT22 cells, an effect abolished by megalin knockdown. These findings support a role for astrocyte‐derived Mt1 in modulating neuronal signaling through megalin. Accumulating evidence indicates that astrocyte–neuron play critical roles in depression pathophysiology [59], particularly in regions such as the orbitofrontal cortex (OFC) [60], and lateral habenula (LHb) [61]. Our findings indicate that SCTR‐expressing astrocytes secrete Mt1, which signals through neuronal megalin to regulate downstream AMPKα2 signaling in serotonergic neurons. Previous work showed that gain or loss of neuronal AMPKα in the mPFC bidirectionally regulates anxiogenic symptoms, and selective knockout of AMPKα in GABAergic neurons induces anxiety‐like phenotypes [36]. In contrast, our data demonstrate that AMPKα2 in DRN serotonergic neurons specifically bidirectionally regulates depressive‐like behaviors, indicating that AMPKα1 in astrocytes and AMPKα2 in serotonergic neurons jointly contribute to depression‐related processes.

Foxc2, a member of the forkhead box (FOX) family of transcription factors [62], has been associated with neurodevelopmental disorders [63]. Here, we demonstrate that Foxc2 in DRN serotonergic neurons regulates depression‐like behaviors. Overexpression of Foxc2 rescues depression‐related behaviors in serotonergic AMPKα2‐deficient mice, identifying Foxc2 as a downstream target of AMPKα2 in this neuronal population. Recent studies have demonstrated that KAT7, a histone acetyltransferase [64], is involved in neuronal damage [65]. Our in vitro data experiments further show that AMPKα2 knockdown reduces KAT7 phosphorylation at S138 residue and suppresses Foxc2 transcription by reducing histone acetylation. Reduced KAT7 phosphorylation does not alter its expression, but disrupts nucleus translocation, decreases KAT7 occupancy and H4K8 acetylation at the Foxc2 promoter, and ultimately suppresses Foxc2 transcription. Further in vivo investigation will be required to fully elucidate these regulatory processes.

The DRN is a heterogeneous structure composed of multiple neuronal subtypes. Serotonergic neurons constitute approximately two‐thirds of DRN neurons and provide approximately 70% of forebrain serotonin [66], with a substantial proportion expressing VGlut3 [67, 68]. In this study, AMPKα2 was selectively deleted in serotonergic, dopaminergic, and GABAergic neurons within the DRN. Among these models, only serotonergic neuron‐specific AMPKα2 deletion produced depressive‐like behaviors. Genetic deletion of AMPKα2 specifically in serotonergic neurons leads to reduced activity of DRN serotonergic neurons, as demonstrated by in vitro electrophysiological assays. Overexpression of Foxc2 was sufficient to rescue the reduced serotonergic neuronal activity and excitability following AMPKα2 deletion. Previous studies have primarily focused on the behavioral effects of modulating serotonin levels in the DRN in the context of depression [69, 70]. Our results demonstrate that Foxc2 overexpression is sufficient to rescue reduced serotonin release in mice with serotonergic neuron‐specific AMPKα2 knockout, consistent with the observed depressive‐like behavioral changes. In the present study, we observed that male mice exhibited more pronounced depression‐like behaviors compared to females, whose behavioral responses may be influenced by fluctuations in hormonal levels. Consistent with previous reports, sex hormones exert profound modulatory effects on DRN serotonergic circuits and astrocyte function [71, 72]. Although direct evidence linking sex‐steroid hormones to SCTR‐AMPK‐KAT7‐Foxc2 cascade remains limited, existing studies support a sex‐dependent control over AMPK signaling and epigenetics [73, 74]. Therefore, this possible differential regulation may contribute to sexual dimorphism in depression‐related behaviors. Thus, the precise modulation of this signaling axis by sex differences warrants further in‐depth study.

In summary, our findings reveal that SCTR signaling within the DRN is involved in regulating the onset of depression. SCTR regulates depression‐like behaviors through AMPKα1 in astrocytes, while reduced Mt1 impairs megalin‐mediated downstream signaling of AMPKα2 in serotonergic neurons. Foxc2 is identified as a key downstream target in these neurons. Characterization of SCTR signaling provides novel insights into the pathophysiological mechanisms underlying depression.

## 4 Experimental Section

### 4.1 Animals

C57BL/6J mice, SCTR knockout mice (*SCTR* ^−^/^−^), *SCTR*‐Cre mice, Ai14^tdTomato^ mice, *ePet*‐Cre mice, *Aldh1l1*‐Cre mice, and *AMPKα2* ^flox/flox^ mice [75] were used in this study. Ai14^tdTomato^ mice (JAX: 007908), *ePet*‐Cre mice(JAX: 012712), and *AMPKα2* ^flox/flox^ mice (JAX: 014142) were procured from the Jackson Laboratory. *SCTR* ^−/−^ mice, and *SCTR‐Cre* mice were purchased from Shanghai Model Organisms Center (Shanghai, China). *Aldh1l1*‐Cre (I001112) mice were purchased from Cyagen (Suzhou, China). C57BL/6J mice were purchased from GemPharmatech (Nanjing, China). For Cre­lox experiments, Ai14^tdTomato^ mice were crossed with *SCTR*‐Cre mice or *ePet*‐Cre mice, and *AMPKα2* ^flox/flox^ mice were crossed with *ePet*‐Cre mice. Male and female mice aged 8–12 weeks were used in the study. Male and female mice were equally represented across all behavioral experiments; however, female estrous cycles were not monitored. Male mice were used for fiber photometry and electrophysiological recordings. All mice were housed in standard cages under controlled environmental conditions (12 h light/12 h dark cycle, lights on at 7:00 a.m.; ambient temperature 22–24°C) with food and water provided ad libitum. All animal care and experimental procedures were approved by the Institutional Animal Care and Use Committee of Binzhou Medical University Hospital (Approval No. 20220622–15).

### 4.2 Stereotaxic Surgery and Adeno‐Associated Virus Infusion

Stereotaxic surgery was performed as previously described [28]. Under anesthesia, 0.30 µL AAV (single virus or 1:1 mixed virus, viral titer >1 × 10^12^ vg/mL) was injected bilaterally into the DRN (coordinates: AP = −4.48 mm, ML = ± 0.4 mm, DV = −3.0 mm from the bregma, with a 15° angle) at a rate of 0.1 µL/min. AAV2/9‐CMV‐DIO‐sh*SCTR*‐GFP, AAV2/9‐CMV‐DIO‐sh*AMPKα1*‐GFP, AAV2/9‐CMV‐DIO‐sh*Foxc2*‐GFP, AAV2/9‐CMV‐DIO‐*AMPKα2*‐GFP, and AAV2/9‐CMV‐DIO‐*Foxc2*‐GFP were purchased from Hanbio Biotechnology (Shanghai, China). The rAAV‐TPH2‐Cre‐P2A‐GFP‐WPRE(PT‐3758), rAAV‐VGAT‐Cre‐GFP‐WPREs(PT‐2713),rAAV‐TH‐Cre‐P2A‐GFP‐WPREs(PT‐5410),rAAV‐EF1α‐DIO‐r5HT2.0‐WPREs(PT‐5288), and rAAV‐EF1α‐DIO‐jRGECO1a‐WPREs(PT‐5497) were purchased from brainVTA (Wuhan, China).

### 4.3 Immunofluorescence Histochemistry

Anesthetized mice were transcardially perfused with pre‐cooled 4% paraformaldehyde. Brains were post‐fixed overnight in 4% paraformaldehyde and dehydrated in 30% sucrose solution. Coronal brain sections (40 µm thick) containing the DRN were cut using a cryostat microtome (Leica Microsystems, Germany). Sections were incubated overnight at 4°C with primary antibodies against anti­NeuN (1:400; ab104224; Abcam), anti­GFAP (1:1, 000; ab7260; Abcam), anti­GABA (1:400; A2052; Sigma–Aldrich), anti­IbaI (1:400; ab5076; Abcam), anti­AMPKα1 (1:400; NB100‐239; Novus Biologicals), anti­AMPKα2 (1:800; ab3760; Abcam), anti­TH (1:400; 22941; ImmunoStar), anti­GAD67 (1:400; MAB5406; Sigma–Aldrich), anti­5‐HT (1:400; ab66047; Abcam), anti­KAT7 (1:800; ab190908; Abcam), and anti­Foxc2 (1:400; AF6989; R&D Systems). Sections were then incubated for 4 h at room temperature with secondary antibodies, including donkey anti‐rabbit Alexa Fluor 488 (1:400; A21206; Invitrogen), goat anti‐mouse Alexa Fluor 647 (1:400; A21236; Invitrogen), rabbit anti‐goat Alexa Fluor 488 (1:400; A11078; Invitrogen), donkey anti‐goat Alexa Fluor 647 (1:400; A32849; Invitrogen), donkey anti‐rabbit Alexa Fluor 546 (1:400; A10040; Invitrogen). Images were acquired using an Olympus FV1000 laser scanning confocal microscope (Olympus, Japan) and processed using ImageJ, with fluorescence signals normalized to 100% as previously described [13].

### 4.4 Cell Cultures and Transfections

Mouse hippocampal neuronal HT22 cells were purchased from the Conservation Genetics CAS Cell Bank (Shanghai, China). HT22 cells were cultured in DMEM supplemented with 10% fetal bovine serum (FBS). Mt1 peptides were synthesized by GenScript (Nanjing, China), and reference peptide sequences were obtained from previously published studies [57, 58]. Mt1 peptide sequences are listed in Table S1. The megalin siRNA were synthesized by Yixiang Biotechnology (Tianjin, China). Megalin siRNA sequences are listed in Table S2. HT22 cells were transiently transfected with the siRNA using Lipofectamine 3000 reagents (Invitrogen, USA). HT22 cells were stimulated with 3.5 mM Mt1 peptides for 1 h for analysis of p‐AMPKα or AMPKα2.

### 4.5 Lentiviral Infection

The lentiviral vector pHBLV‐U6‐MCS‐CMV‐GFP‐PGK‐PURO (Hanbio, China) was used to package shRNAs targeting AMPKα2 and KAT7. The shRNAs were designed and synthesized by Hanbio (Shanghai, China). Forty‐eight hours after transfection, HT22 cells were treated with puromycin (2 µg/mL) to select for positive cells. Limiting dilution was then performed to isolate single‐cell clones [76]. Clones exhibiting the lowest AMPKα2 and KAT7 expression were selected for subsequent experiments. Target sequences of the shRNAs are listed in Table S3.

### 4.6 Co‐Immunoprecipitation

The co‐immunoprecipitation Kit was purchased from Bersin Bio (Guangzhou, China). For co‐immunoprecipitation assay, HT22 cells were transiently transfected with the indicated plasmids using Lipofectamine 3000 reagents (Invitrogen, USA). The pcDNA3.1‐3 × HA‐KAT7‐T2A‐RFP, pcDNA3.1‐3 × Flag‐AMPKα2‐T2A‐EGFP, pcDNA3.1‐3 × HA‐KAT7S138A‐T2A‐RFP, pcDNA3.1‐3×Flag‐KAT7‐T2A‐EGFP, pcDNA3.1‐3 × HA‐T2A‐RFP, and pcDNA3.1‐3 × Flag‐T2A‐EGFP plasmids were purchased from Yixiang Biotechnology (Tianjin, China).

### 4.7 Western Blotting

Western blotting was performed as previously described [13]. DRN tissue or HT22 cells were homogenized in lysis buffer (Beyotime Biotechnology, China) containing 1% PMSF (Solarbio, China) and 1× PhosSTOP (Roche, Germany). Proteins (∼25 µg) were separated by sodium dodecyl sulfate‐polyacrylamide gel electrophoresis (SDS‐PAGE) and transferred to PVDF membranes (Millipore, USA). Membranes were blocked with 5% nonfat milk for 1 h at room temperature and incubated overnight at 4°C with primary antibodies against SCTR (1:400; PA5‐76698; Invitrogen), AMPKα1 (1:400; NB100‐239; Novus Biologicals), AMPKα2 (1:400; ab3760; Abcam), p‐AMPKα(1:400; 2535; Cell Signaling), Foxc2 (1:400; AF6989; R&D Systems), KAT7 (1:400; ab190908; Abcam), GAPDH (1:2000; 2118; Cell Signaling), H4ac (1:400; ab177790; Abcam), β‐actin (1:400; 3700; Cell Signaling), H3ac (1:400; ab300641; Abcam), p‐S138‐KAT7 (1:100; Abmart), p‐S162‐KAT7 (1:100; Abmart), H3acK14(1:400; ab52946; Abcam), H3acK23 (1:400; ab177275; Abcam), H4acK5 (1:400; ab51997; Abcam), H4acK8 (1:400; ab45166; Abcam), H4acK12 (1:400; ab177793; Abcam), histone H4 (1:400; ab177840; Abcam), Jade1 (1:400; 15032‐1‐AP; proteintech), histone H3 (1:400; ab61251; Abcam). Membranes were then incubated for 1 h at room temperature with IRDye 680RD donkey anti‐mouse (1:5000; 926–68072; LI‐COR), IRDye 800CW donkey anti‐goat (1:5000; 926–32214; LI‐COR), IRDye 800CW goat anti‐rabbit (1:5000; 926–32211; LI‐COR) secondary antibodies. Signals were detected and analyzed using an Odyssey Infrared Imaging System (Li‐COR, USA).

### 4.8 Enzyme‐Linked Immunosorbent Assay Kit (ELISA)

Plasma secretin levels were measured using a secretin ELISA (Phoenix Pharmaceuticals, Inc., USA). All samples were measured in duplicate according to the manufacturer's instructions.

### 4.9 Quantitative Real‐Time PCR

As previously described [13], total RNA from DRN tissue or HT22 cells was extracted using TRIzol reagent (Invitrogen, USA). Reverse transcription was performed using HiScript II QRT SuperMix (Vazyme, China), and the resulting cDNA was used for q‐PCR analysis on a StepOnePlus real‐time PCR system (Applied Biosystems, USA). β‐tubulin was used as the reference gene, relative expression levels were calculated using the 2^−ΔΔCT^ method. Primer sequences are listed in Table S4.

### 4.10 Chromatin Immunoprecipitation

DRN tissue or HT22 cells were washed with ice‐cold PBS and crosslinked with 1% formaldehyde at room temperature for 15 min. Crosslinking was quenched with 125 mM glycine for 5 min, followed by two washes with ice‐cold PBS. Tissues or cells were homogenized in lysis buffer containing 1% PMSF, sonicated, and centrifuged. The resulting chromatin was incubated with 4 µL of anti‐H4acK8, anti‐KAT7, or anti‐IgG, and DNA‐protein complexes were captured using protein A/G magnetic beads. Complexes were washed with TE buffer, treated with 0.2 M NaCl, and incubated at 65°C overnight to reverse crosslinks. After proteinase K digestion, DNA was purified using a DNA purification kit, and q‐PCR was performed using primers targeting the Foxc2 promoter. Primer sequences are listed in Table S4.

### 4.11 RNA Sequencing Analysis

DRN tissue was collected from *AMPKα2* ^flox/flox^ mice and *AMPKα2* ^flox/flox; ePet‐Cre^ mice and sequenced by Genechem Technology (Shanghai, China). Total RNA was extracted, and cDNA libraries were prepared using the TruSeq RNA Sample Preparation Kit (Illumina, USA). Sequencing was performed on an Illumina sequencing platform at Genechem Technology. Differentially expressed genes were identified using thresholds of *p* < 0.05 and |log2FC| > 1.

### 4.12 Proteomics and Phosphoproteomics

Proteomics and phosphoproteomics experiments were performed by PTM Biolabs (Hangzhou, China). Briefly, proteins extracted from DRN tissue or HT22 cells were digested with trypsin and labeled with TMT tags. Samples were desalted using Strata X C18 SPE columns (Phenomenex, USA), and the peptides were analyzed by liquid chromatography–mass spectrometry (LC‐MS). For phosphoproteomics, samples were enriched using immobilized metal ion affinity chromatography (IMAC) prior to LC‐MS analysis. Peptides were separated using an EASY‐nLC 1200 UPLC system (Thermo Fisher Scientific, USA) and analyzed on an Exploris480 mass spectrometer (Thermo Fisher Scientific, USA). Bioinformatics analyses were performed as part of the workflow.

### 4.13 Whole‐Cell Patch‐Clamp Recordings

Briefly, mice were anesthetized with isoflurane and decapitated, and the brains were rapidly dissected and transferred to ice‐cold artificial cerebrospinal fluid (aCSF). Coronal brain slices (300 µm thick) containing DRN were prepared using a Leica VT1200 vibratome (Leica, Germany) and transferred to a water bath incubator in oxygenated aCSF (95% O2 and 5% CO2) before recording. Slices were then transferred to a recording chamber and recorded at room temperature. The chamber was continuously perfused with the extracellular bath aCSF during recordings. Recording pipettes with tip resistances of 3–5 MΩ were pulled from thin‐walled borosilicate glass capillaries (Sutter Instrument, USA) using a Sutter P‐97 (Sutter Instrument, USA). Pipettes were filled with potassium gluconate‐based internal solution (pH 7.4; osmolarity 295–300 mOsm). Spontaneous neuronal firing was recorded in zero‐current mode (I  =  0), and the resting membrane potential was measured immediately after achieving whole‐cell access.

### 4.14 RNA Fluorescence in Situ Hybridization (FISH)

RNA fluorescence in situ hybridization (RNA FISH) was performed as previously described [28]. RNA FISH probe kit (GenePharma, China) was used according to the manufacturer's instructions. Brain slices were fixed with 4% paraformaldehyde at room temperature for 1 h, washed twice with PBS for 5 min, and incubated with proteinase K digestion solution at 37°C for 20 min. Slices were incubated with blocking buffer at 37°C for 30 min and then treated with denaturation solution at 78°C for 8 min. Sections were dehydrated through a graded ethanol series (70% to 100%, 2 min per step) and hybridized with Mt1 probes overnight at 37°C. Nuclei were counterstained with DAPI for 15 min at room temperature. Images were acquired using an Olympus FV1000 laser scanning confocal microscope (Olympus, Japan) and processed using ImageJ software. SCTR and Mt1 probe sequences are listed in Table S5.

### 4.15 Fiber Photometry Recording

A multi‐channel fiber photometry system was purchased from RWD Life Sciences (Shenzhen, China). To record 5‐HT neurotransmitter signals and calcium activity, AAV‐EF1α‐DIO‐r5HT2.0‐WPRE or AAV‐EF1α‐DIO‐jRGECO1a‐WPREs viruses were injected into the DRN, followed by implantation of an optical fiber above the injection site. After three weeks, fluorescence signals were recorded at a wavelength of 560 nm. Prior to testing, mice were habituated to the test apparatus for 3 min to minimize stress‐induced behavioral artifacts. Three tail suspension trials, each lasting 6 s, were then performed with a 2‐min interval between trials. 5‐HT transients and calcium dynamics were recorded via fiber photometry. The onset of tail suspension was defined as time zero. Data were analyzed using the recording software provided.

### 4.16 Chronic Unpredictable Stress (CUS)

The CUS procedure was carried out as previously described [77]. Briefly, mice were individually housed and subjected to two different stressors daily for 14 days. Stressors included restraint (2 h), wet bedding (24 h) with a 45° cage tilt, inescapable foot shocks, elevated platform exposure (30 min), constant light (24 h), and tail pinch (15 min).

### 4.17 Female Urine Sniffing Test (FUST)

Prior to testing, male mice were housed individually. One hour before the experiment, mice were acclimated to cotton swabs clamped with a hemostat and placed on the inner cage lid. During testing, each mouse was sequentially exposed for 3 min to two types of cotton swabs: one moistened with water and the other with 80 µL urine from estrous female mice (freshly collected from mice of the same strain). A 45‐min interval was maintained between exposures. Time spent sniffing each swab was recorded.

### 4.18 Sucrose Preference Test (SPT)

Prior to testing, two water bottles were placed in the cage at the maximum possible distance, and mice were acclimated for one week. Four hours before testing, mice were individually housed without access to water. During testing (19:00–21:00), mice were given free access to both water and 1% sucrose solution. Sucrose preference was calculated as the percentage of sucrose solution consumption relative to total fluid intake.

### 4.19 Forced Swim Test (FST)

Mice were placed in a cylindrical container (25 cm height, 10 cm diameter) filled with 15 cm of water maintained at 24°C. A 6‐min video recording was obtained, and immobility time was quantified during the final 4 min. Immobility was defined as floating with only minimal movements required to maintain balance and respiration.

Novelty‐suppressed feeding test (NSFT): Mice were food‐deprived for 24 h and tested the following afternoon. The apparatus consisted of a 60 cm × 60 cm × 40 cm open field box lined with 2 cm corncob bedding, with a round filter paper (15 cm diameter) placed at the center. Mice were introduced into the arena from the same corner with a consistent head orientation. Latency to feed (time to first food consumption) was recorded, with a maximum duration of 10 min. Food intake was measured after 5, 10, and 30 min.

### 4.20 Open Field Test (OFT)

The OFT was conducted in a 60 cm × 60 cm × 40 cm arena under uniform illumination. Mice were placed in the center of the arena, and spontaneous locomotor activity was recorded for 5 min. The arena was cleaned with 20% ethanol between trials to eliminate olfactory cues. Time spent in the center zone and total distance traveled were analyzed using ANY­maze software.

### 4.21 Elevated Plus Maze Test (EPMT)

The EPM consisted of four arms (30 cm length, 5 cm width) arranged in a cross, with a central platform (5 cm × 5 cm). During testing, mice were placed in the central and allowed to freely explore the open and closed arms. A camera recorded activity for 5 min, and time spent in each arm and the number of entries into each arm were quantified.

### 4.22 Light‐Dark Box Test (LDT)

The apparatus consisted a light chamber (27 cm × 27 cm × 30 cm) and a dark chamber (18 cm × 27 cm × 30 cm) connected by a partition allowing free passage. The light chamber was illuminated at 700 lux, whereas the dark chamber was fully enclosed. Mice were placed in the center of the dark chamber, and latency to first entry into the light chamber was recorded. The number of entries into and time spent in the light chamber were measured over 5 min.

### 4.23 Locomotor Activity

Locomotor activity was assessed using SuperFlex Fusion open‐field chambers (40 cm × 40 cm × 40 cm; Omnitech Electronics, USA). Mice were placed in the center of the chamber and allowed to freely explore for 30 min. Total distance traveled and locomotor trajectories were automatically recorded by using Fusion Software.

### 4.24 Phosphorylation Sites Prediction of the KAT7 Gene

Phosphorylation sites were predicted using the GPS_6.0 software (https://gps.biocuckoo.cn/download.php) [78, 79]. GPS_6.0 was used to identify potential phosphorylation sites in the KAT7 gene. Predicted phosphorylation sites were examined in the commonly used HT22 cell line.

### 4.25 Statistical Analysis

All data were presented as means ± S.E.M. Statistical analyses were performed using GraphPad Prism 10 (GraphPad Software, USA). The Shapiro‐Wilk test and F‐test were used to assess normality and homogeneity of variance, respectively. For comparisons between two independent groups, a two‐tailed unpaired Student's t test was used for normally distributed data, whereas the Mann‐Whitney U test was applied for non‐normally distributed data. For normally distributed data with unequal variances, two‐tailed unpaired t tests with Welch's correction were used. Comparisons among three groups were performed using one‐way analysis of variance (ANOVA) followed by Sidak's post hoc test for normally distributed data, or the Kruskal‐Wallis test followed by Dunn's post hoc test for non‐normally distributed data. For analyses involving two factors and their interaction, two‐way ANOVA was performed followed by Holm‐Sidak's post hoc test. Statistical significance was defined as *P* < 0.05 for all tests. The sample sizes and statistical details were presented in the figure legends. Western blotting signals were normalized to controls to eliminate confounding sources of variation, which was set to 100%, and all other experimental values were presented as relative fold changes following data transformation. No samples were excluded, and all datasets were subsequently analyzed. Experiments were performed under blinded conditions to minimize assessment bias.

## Author Contributions

C. L., W. L., and D. W. conceptualized the project, designed the experiments, and wrote the manuscript. F.‐T.M. performed and analyzed the main experiments and bioinformatics datasets. J.L., J.‐J.D., M.W., H.‐F.L., W.‐T.W., M.‐D.Z., S.‐J.J., Y.‐F.C., L.‐H.X., L.D., D.W., D.Z., and G.‐F.Q. performed the experiments. W.‐TW performed the electrophysiological studies.

## Conflicts of Interest

The authors declare no conflicts of interest.

## Ethics Approvals

All animal care and experimental procedures were conducted in strict accordance with protocols approved by the Institutional Animal Care and Use Committee of Binzhou Medical University Hospital (Approval No. 20220622–15).

## Supporting information




**Supporting File 1**: advs77985‐sup‐0001‐DatasetS1.xlsx.

**Supporting File 2**: advs77985‐sup‐0002‐DatasetS2.xlsx.

**Supporting File 3**: advs77985‐sup‐0003‐DatasetS3.xlsx.

**Supporting File 4**: advs77985‐sup‐0004‐DatasetS4.xlsx.

**Supporting File 5**: advs77985‐sup‐0005‐SuppMat.pdf.

**Supporting File 6**: advs77985‐sup‐0006‐SuppMat.docx.

## Data Availability

The data that support the findings of this study are available from the corresponding author upon reasonable request.
