# Supplementary material for: SCTR–Expressing Astrocyte–Serotonergic Neuron Signaling Drives Sexual Dimorphism in Depression Through KAT7/H4ac–Foxc2 Epigenetic Axis in an AMPK‐Dependent Manner
Source: Adv Sci (Weinh). 2026 Sep 27:e77985. Online ahead of print. doi: 10.1002/advs.77985 (PMC13616394; doi:10.1002/advs.77985)

Figure 1I

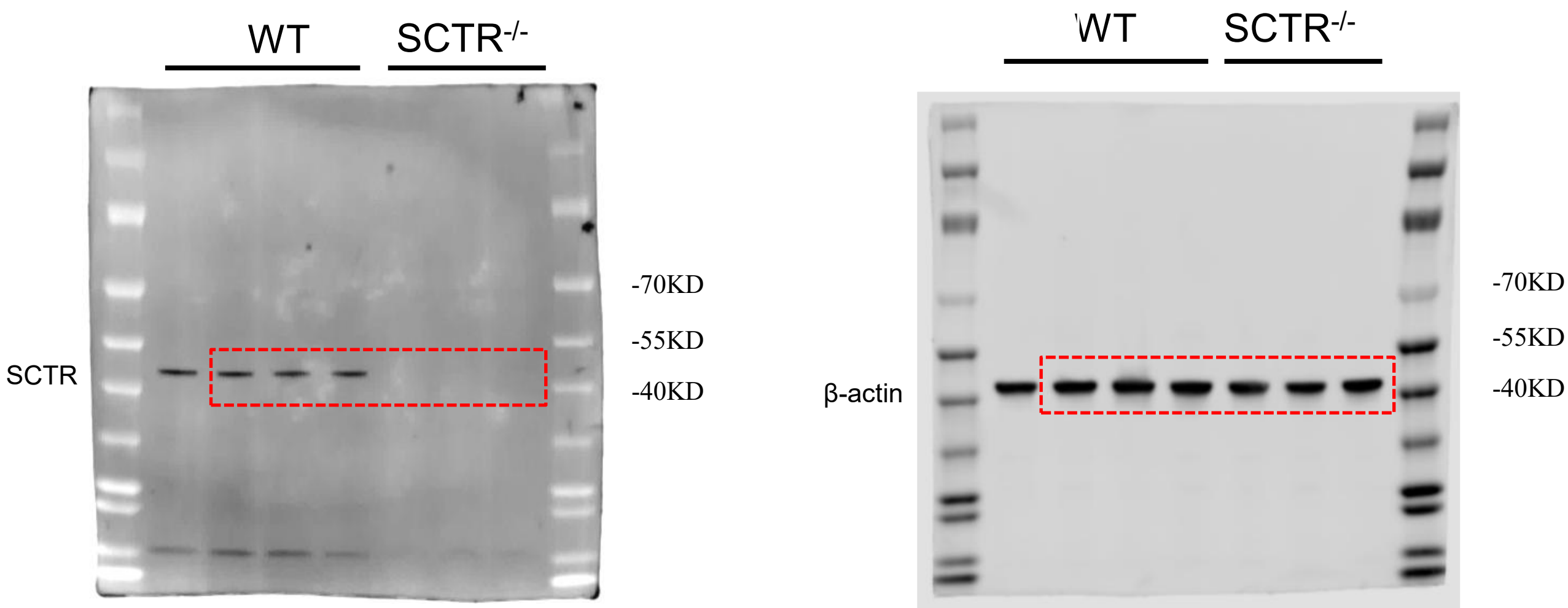

Figure 2F

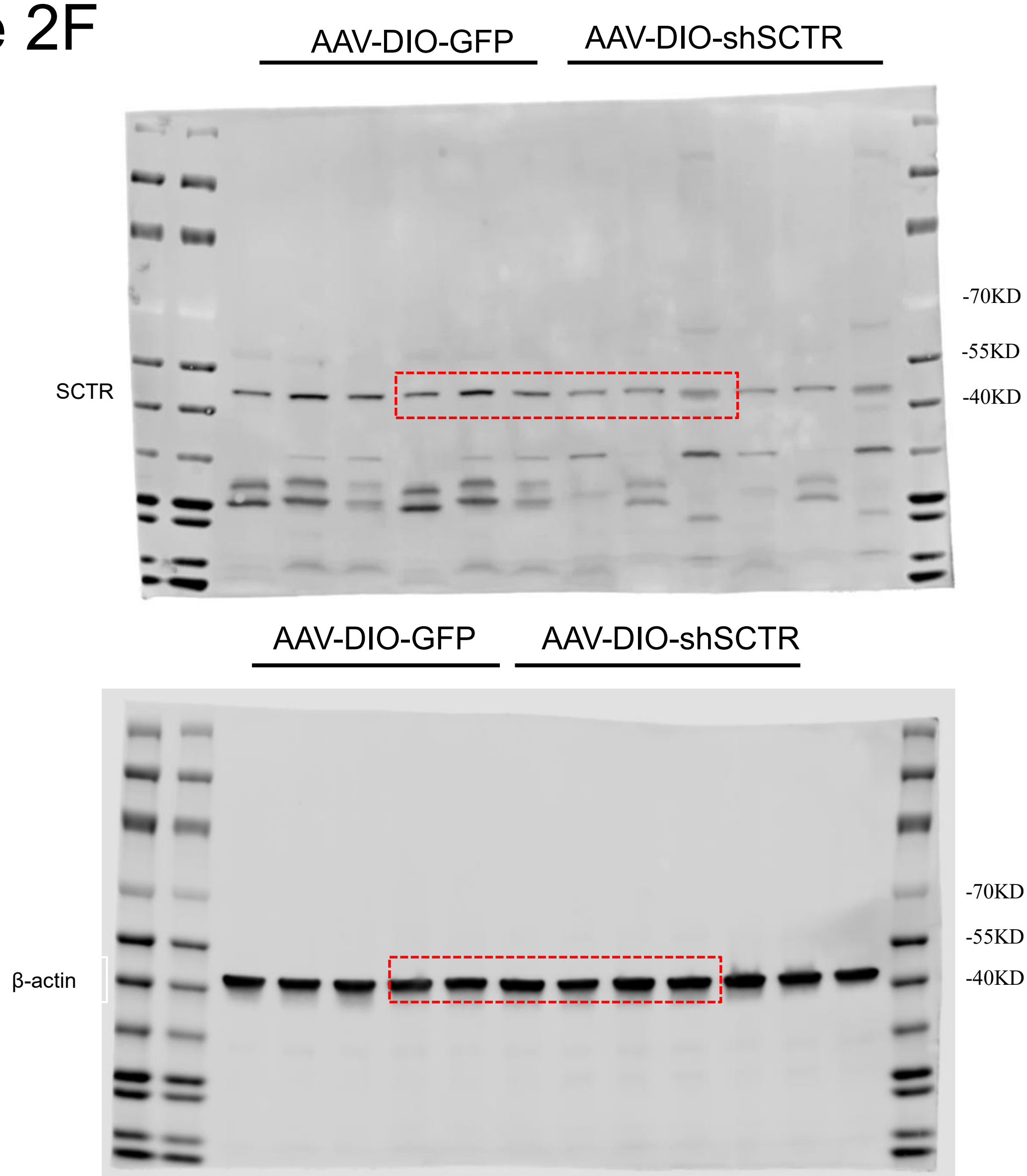

Figure 3C

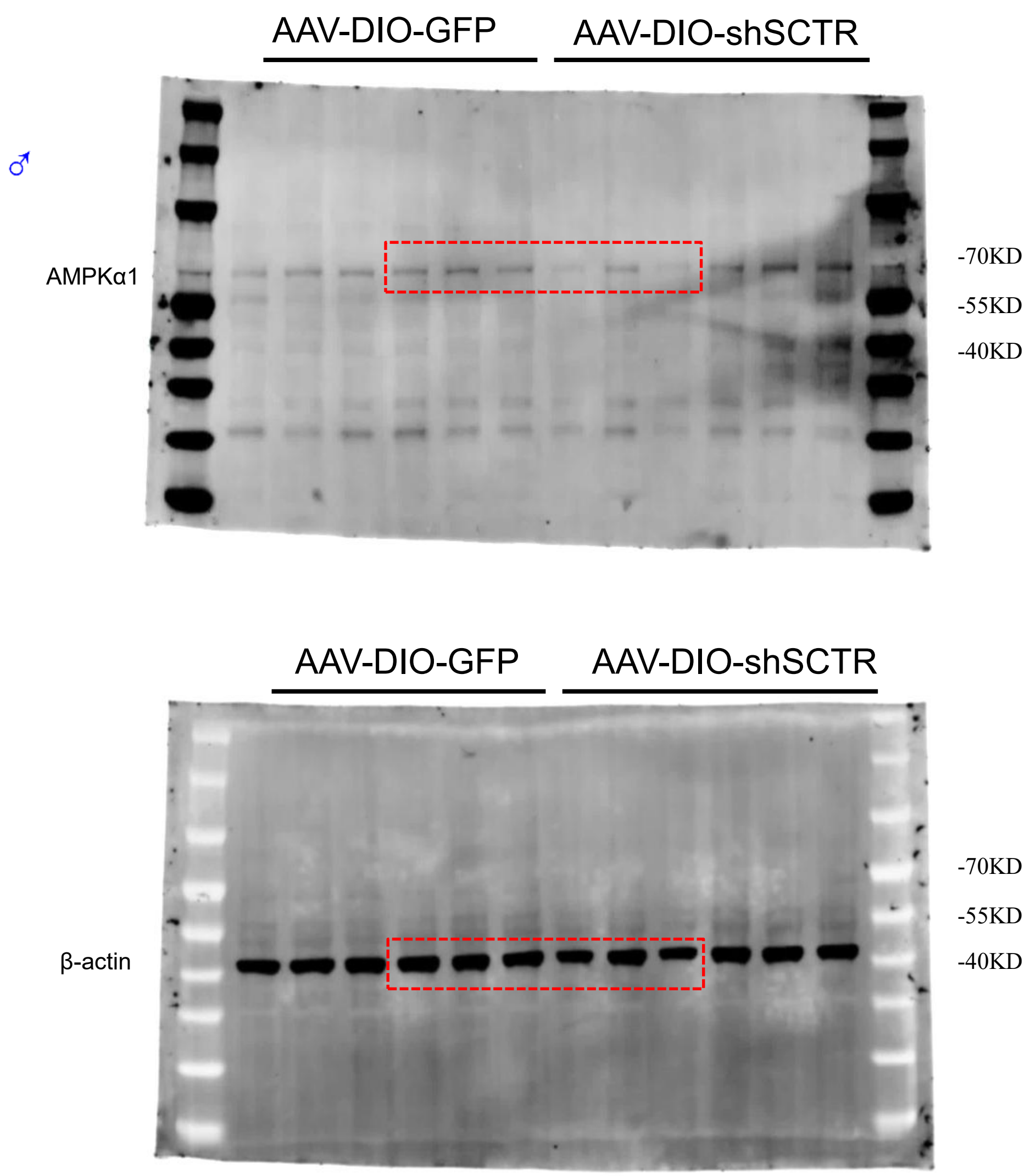
AAV-DIO-GFP
AAV-DIO-shSCTR
♂
AMPKα1
-70KD
-55KD
-40KD
AAV-DIO-GFP
AAV-DIO-shSCTR
β-actin
-70KD
-55KD
-40KD

Figure 3C

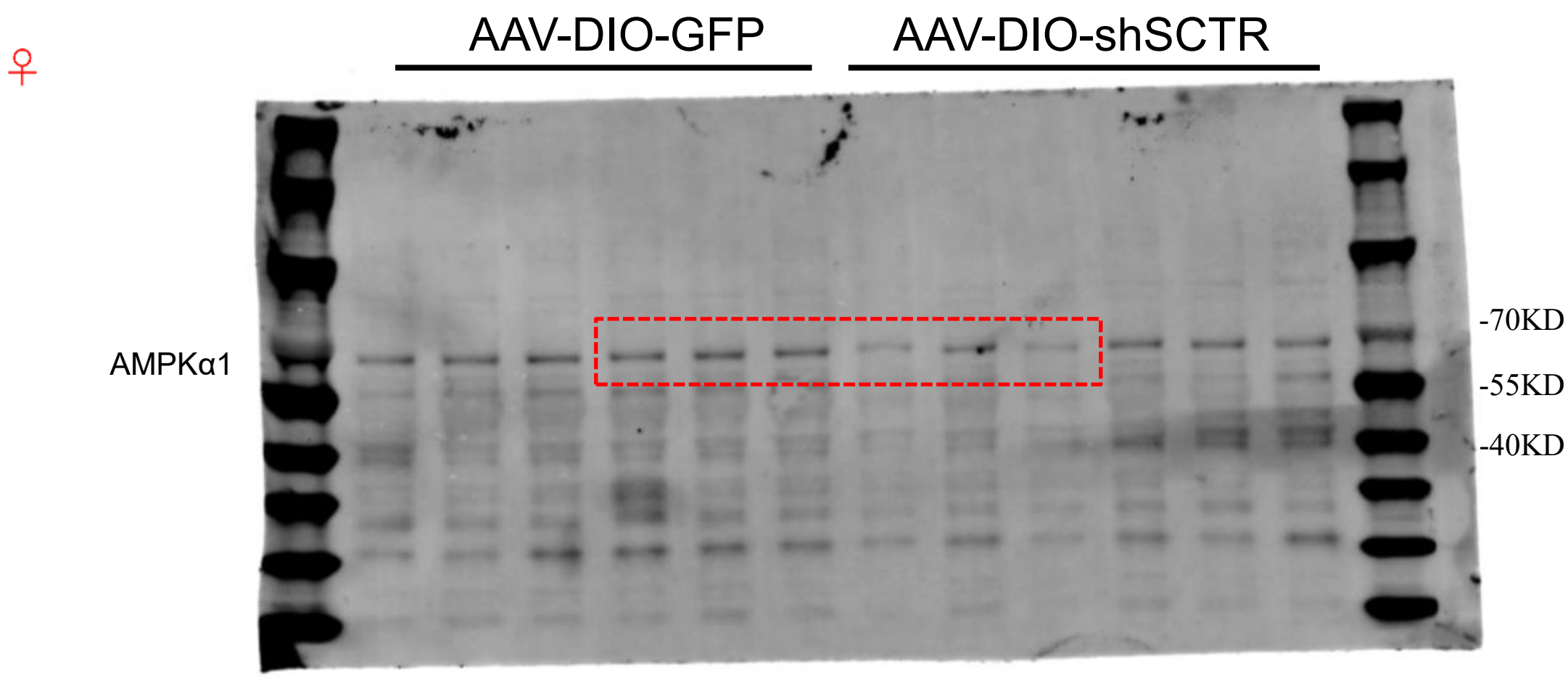
♀
AAV-DIO-GFP
AAV-DIO-shSCTR
AMPKα1
-70KD
-55KD
-40KD

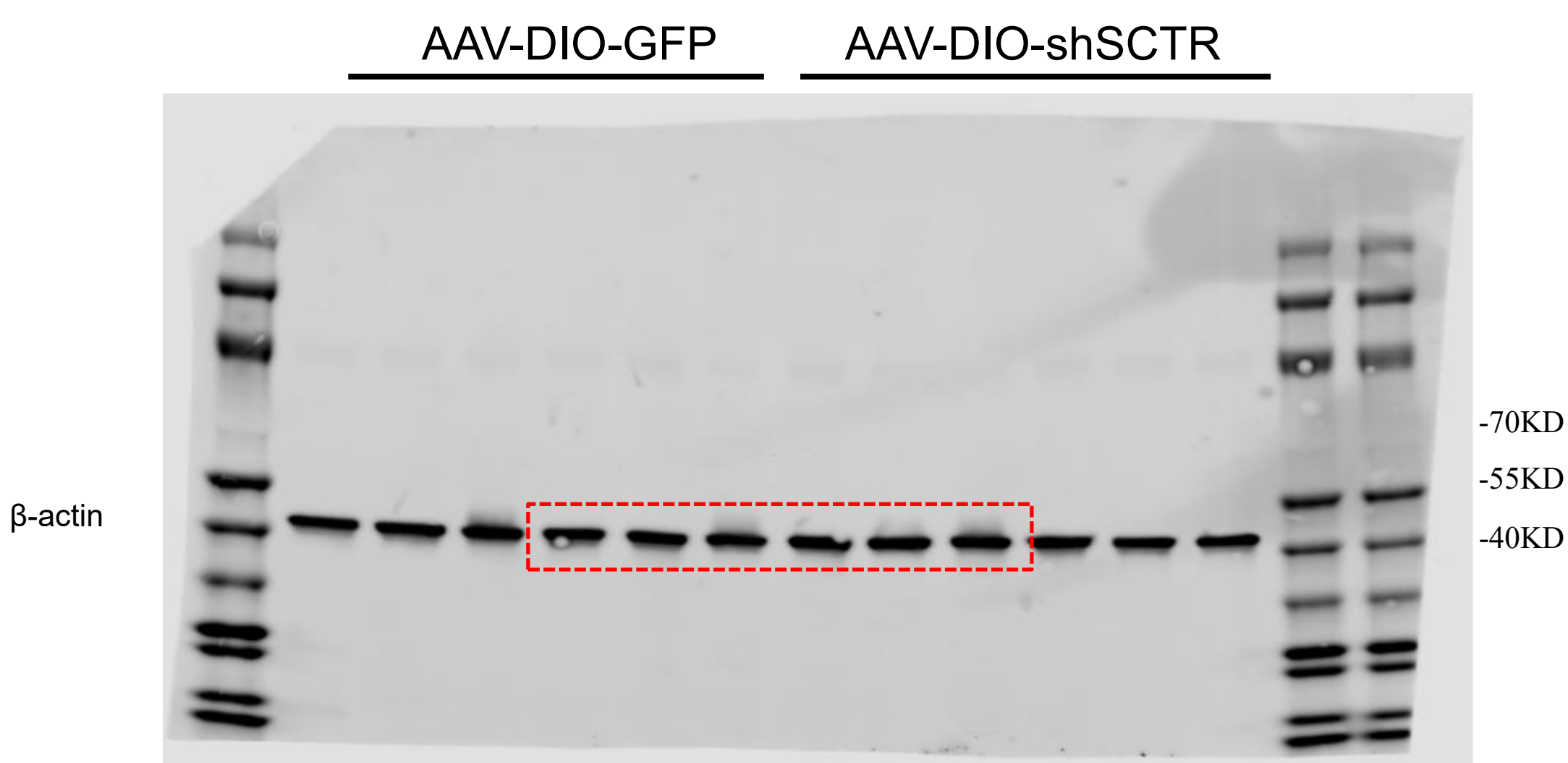
AAV-DIO-GFP
AAV-DIO-shSCTR
β-actin
-70KD
-55KD
-40KD

Figure 3E

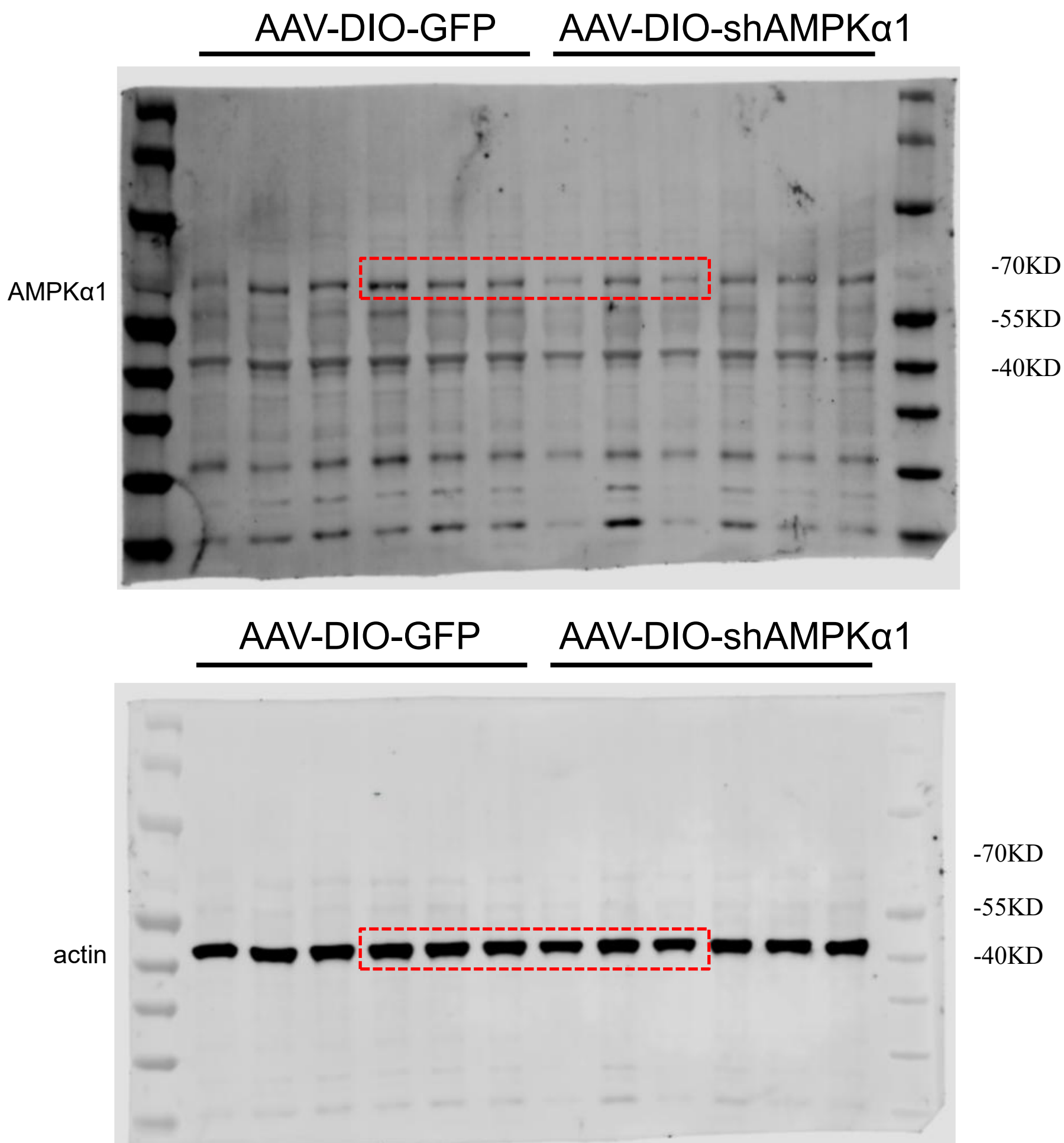

AAV-DIO-GFP
AAV-DIO-shAMPKα1
AMPKα1
-70KD
-55KD
-40KD
AAV-DIO-GFP
AAV-DIO-shAMPKα1
actin
-70KD
-55KD
-40KD

Figure 4F

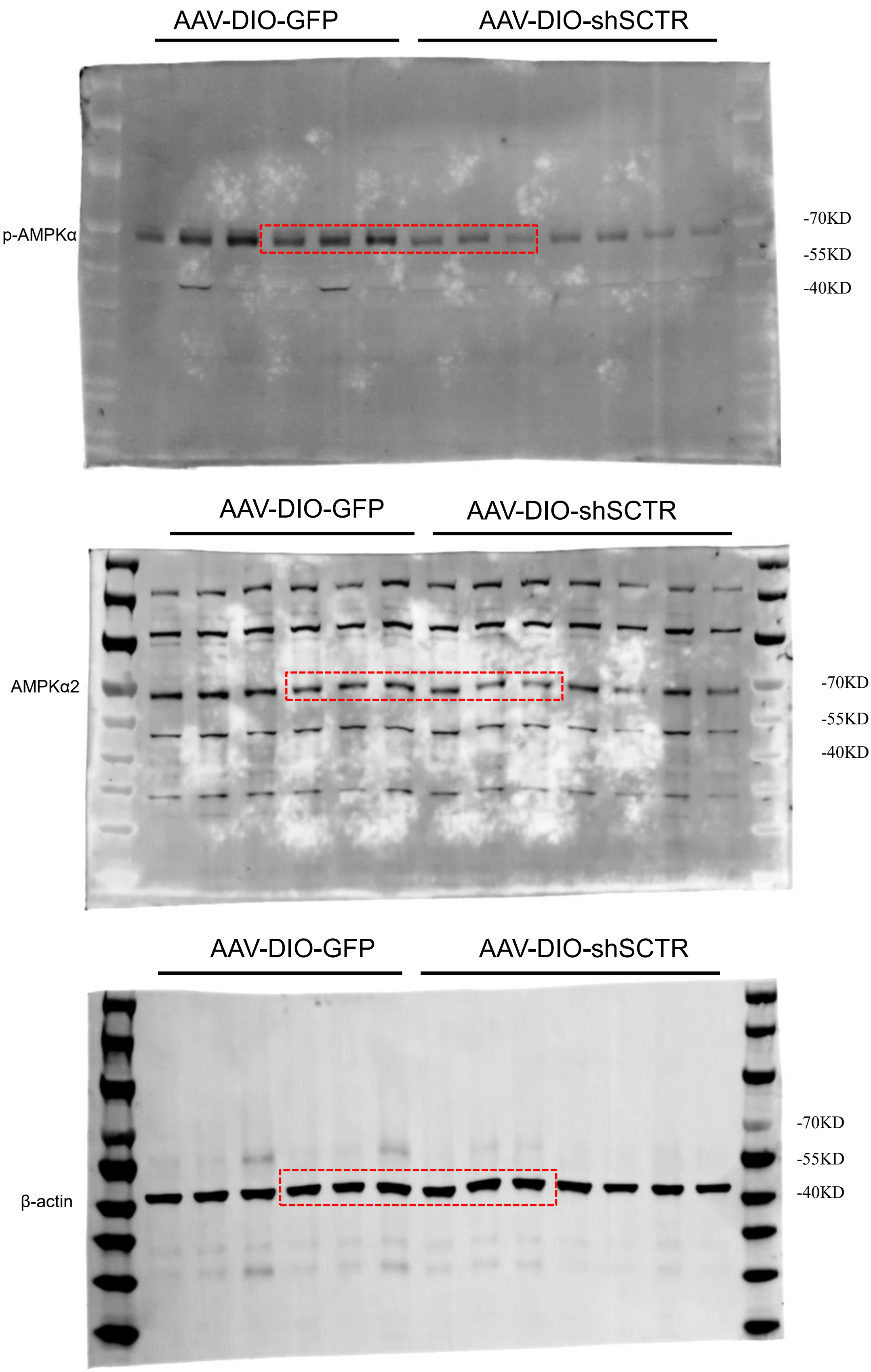
AAV-DIO-GFP
AAV-DIO-shSCTR
p-AMPKα
-70KD
-55KD
-40KD
AAV-DIO-GFP
AAV-DIO-shSCTR
AMPKα2
-70KD
-55KD
-40KD
AAV-DIO-GFP
AAV-DIO-shSCTR
β-actin
-70KD
-55KD
-40KD

Figure 4H

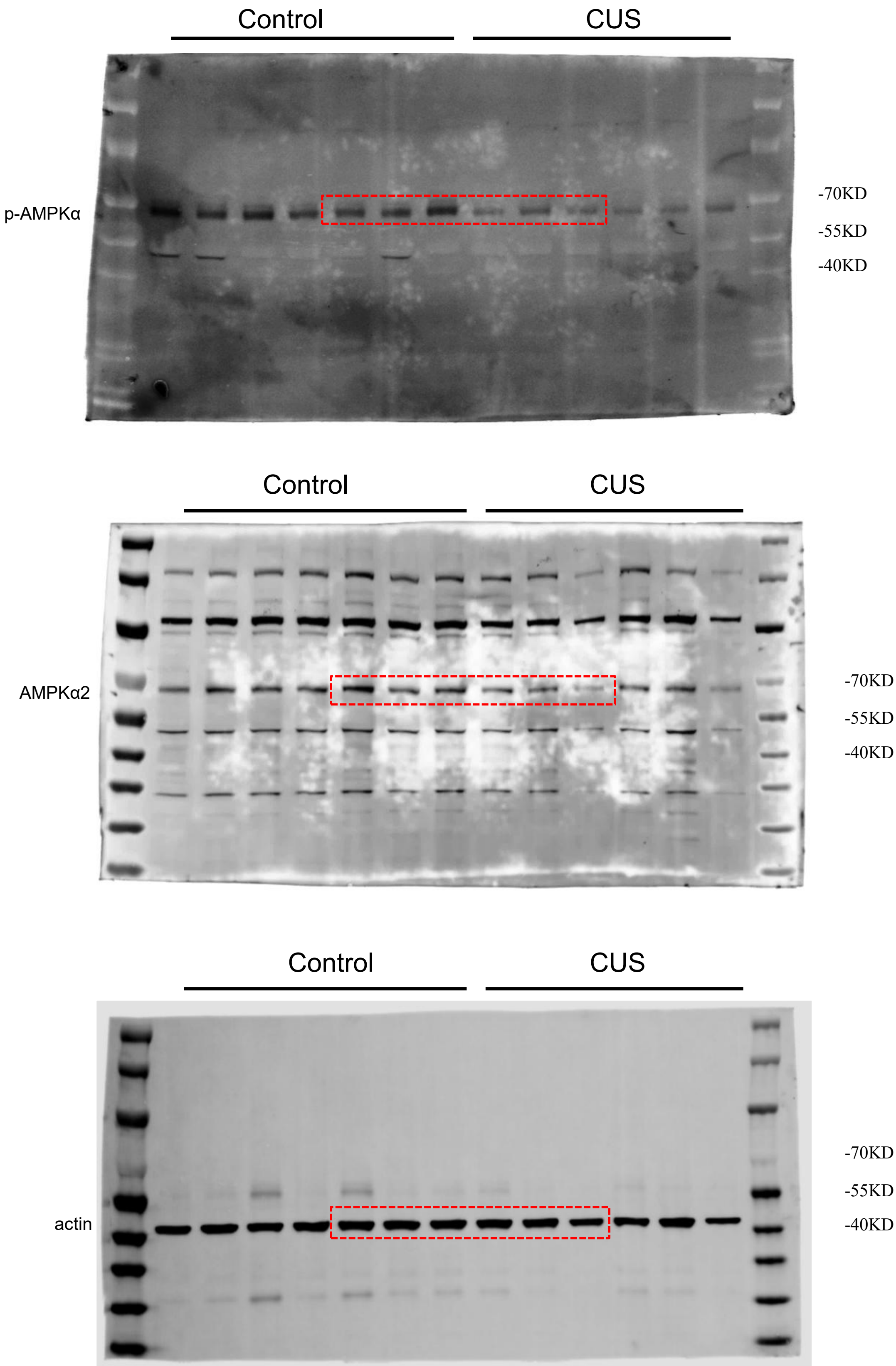

Control
CUS
p-AMPKα
-70KD
-55KD
-40KD
Control
CUS
AMPKα2
-70KD
-55KD
-40KD
Control
CUS
actin
-70KD
-55KD
-40KD

Figure 4L

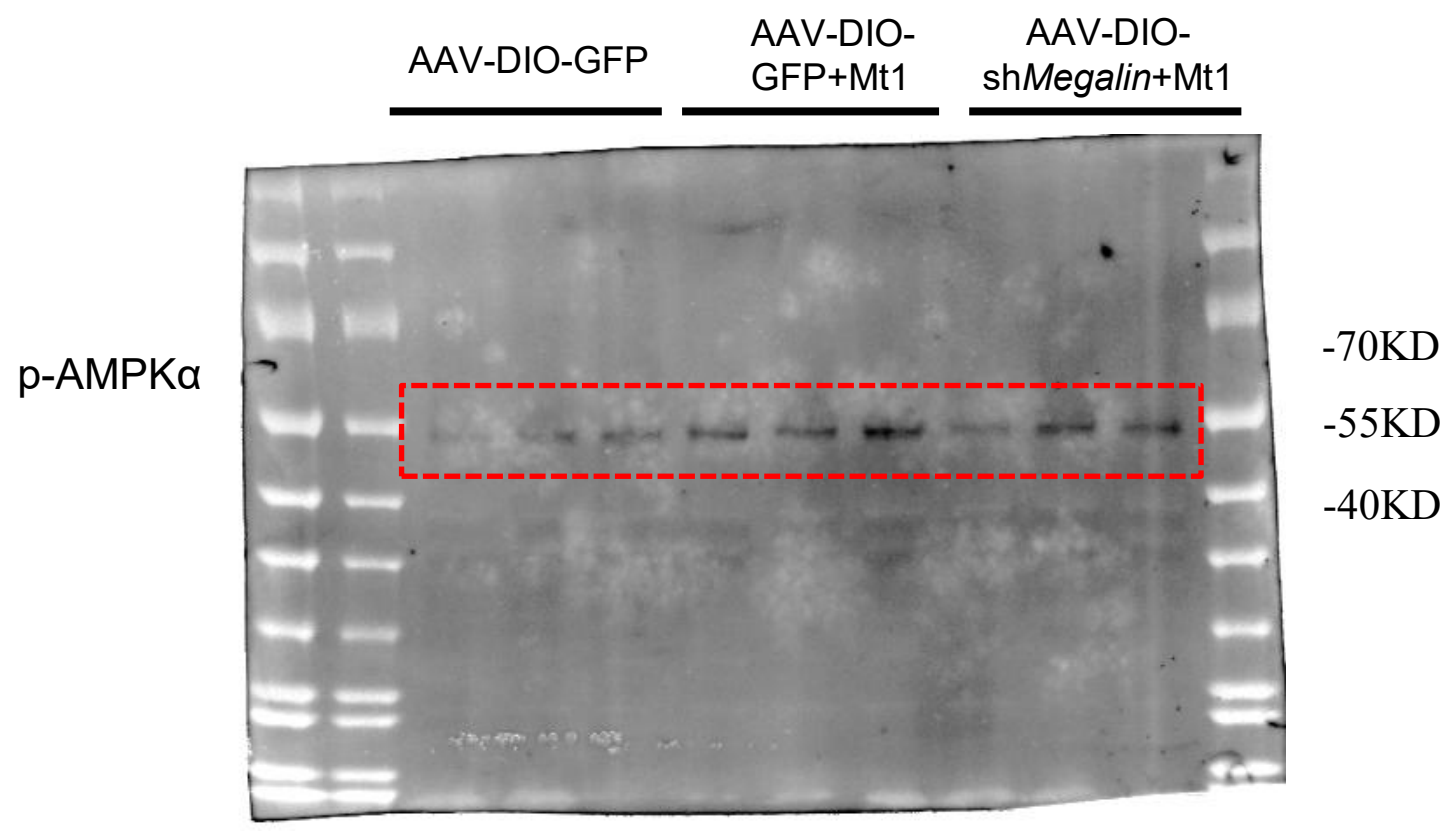

AAV-DIO-GFP
AAV-DIO-GFP+Mt1
AAV-DIO-shMegalin+Mt1
p-AMPKα
-70KD
-55KD
-40KD

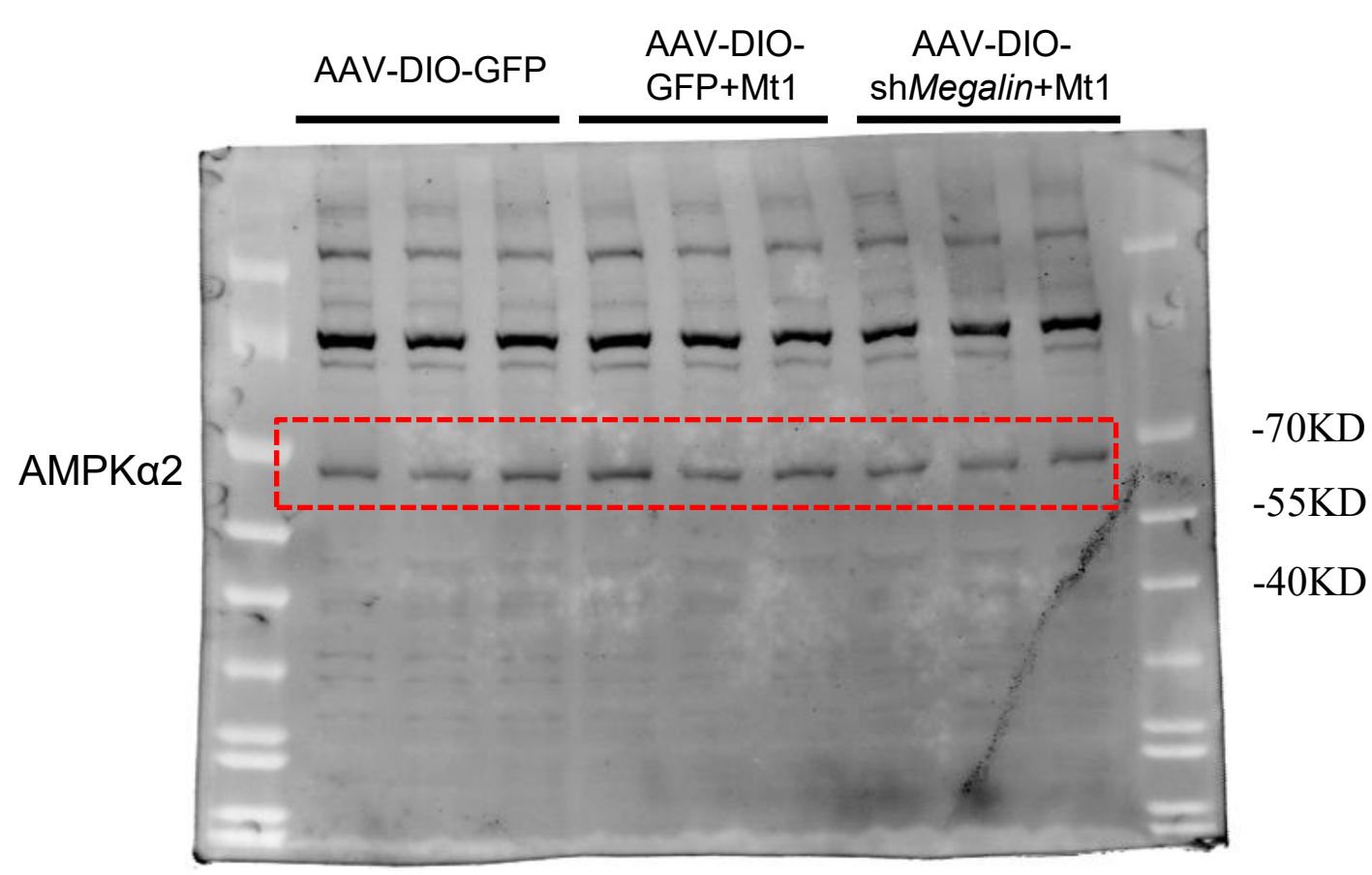

AAV-DIO-GFP
AAV-DIO-GFP+Mt1
AAV-DIO-shMegalin+Mt1
AMPKα2
-70KD
-55KD
-40KD

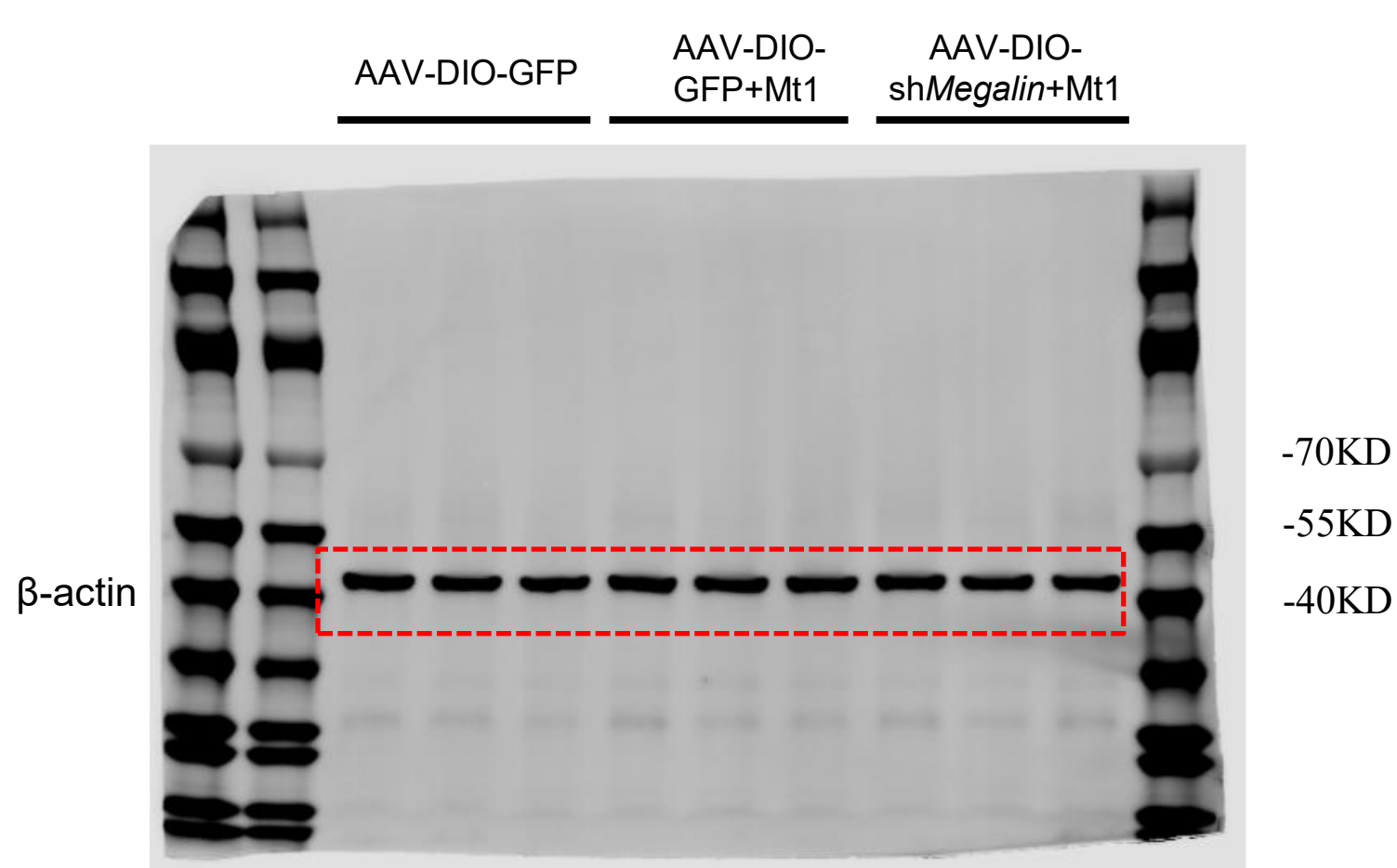

AAV-DIO-GFP
AAV-DIO-GFP+Mt1
AAV-DIO-shMegalin+Mt1
β-actin
-70KD
-55KD
-40KD

Figure 4M

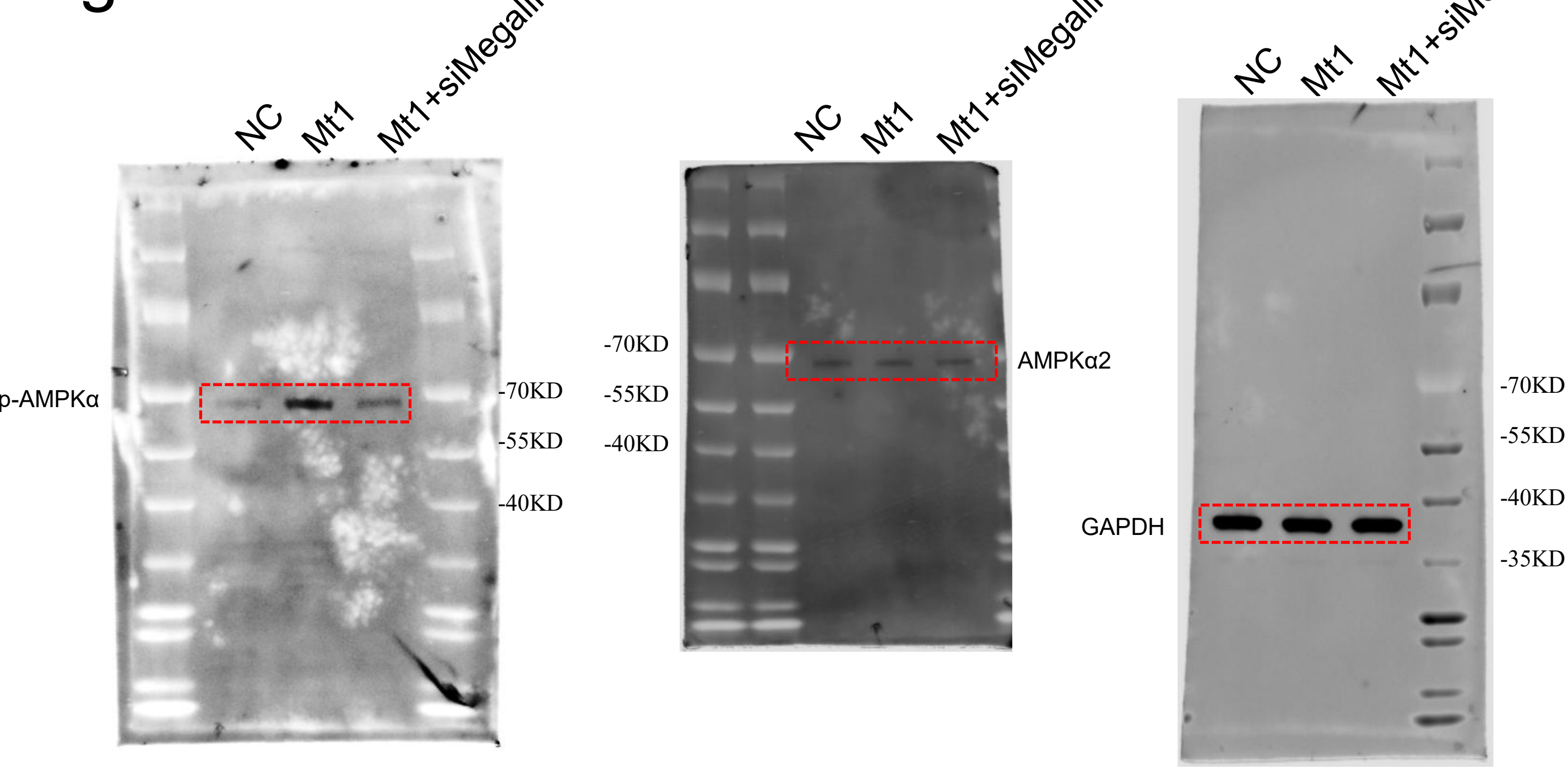

Figure 5D

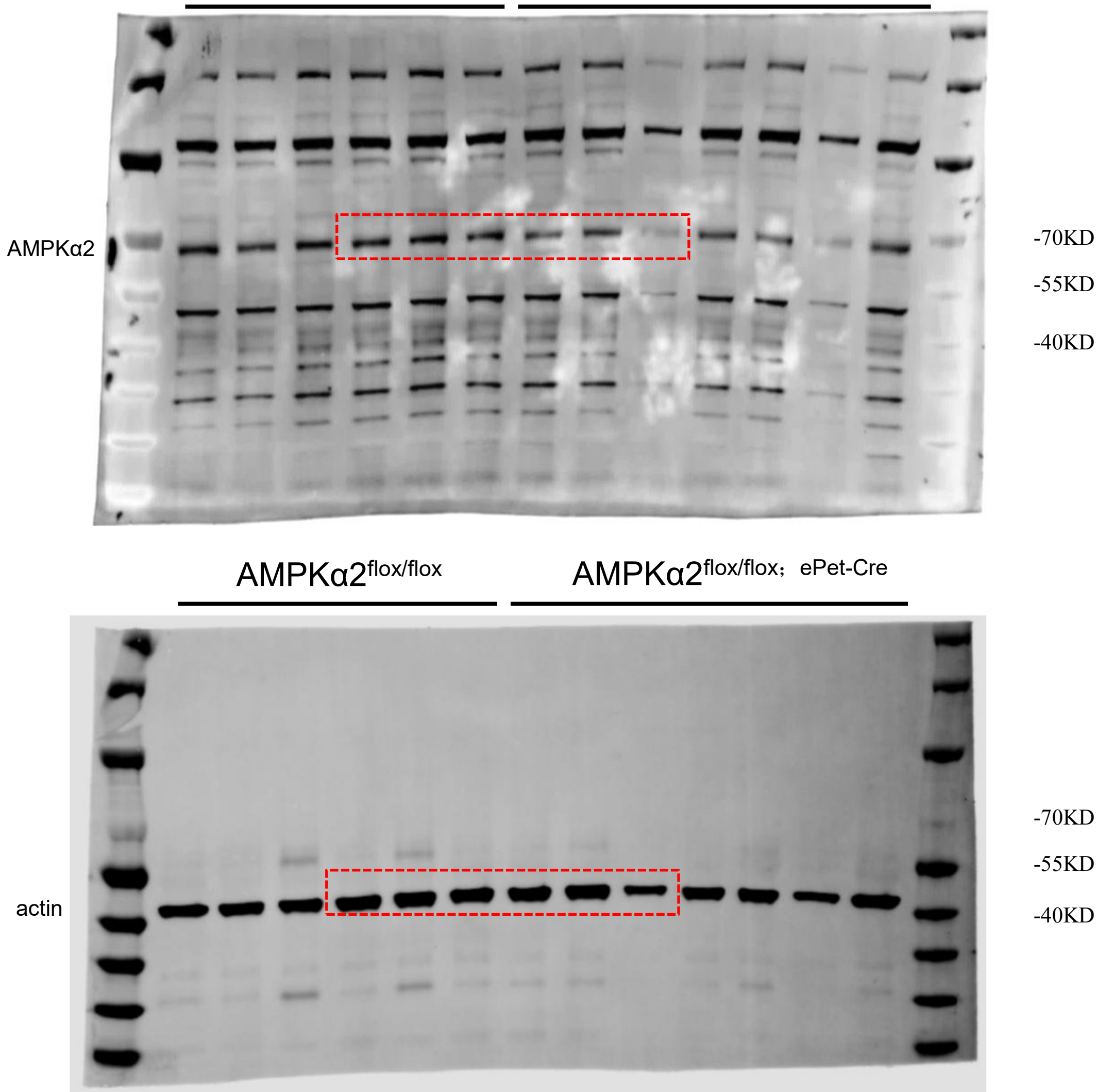

Figure 5M

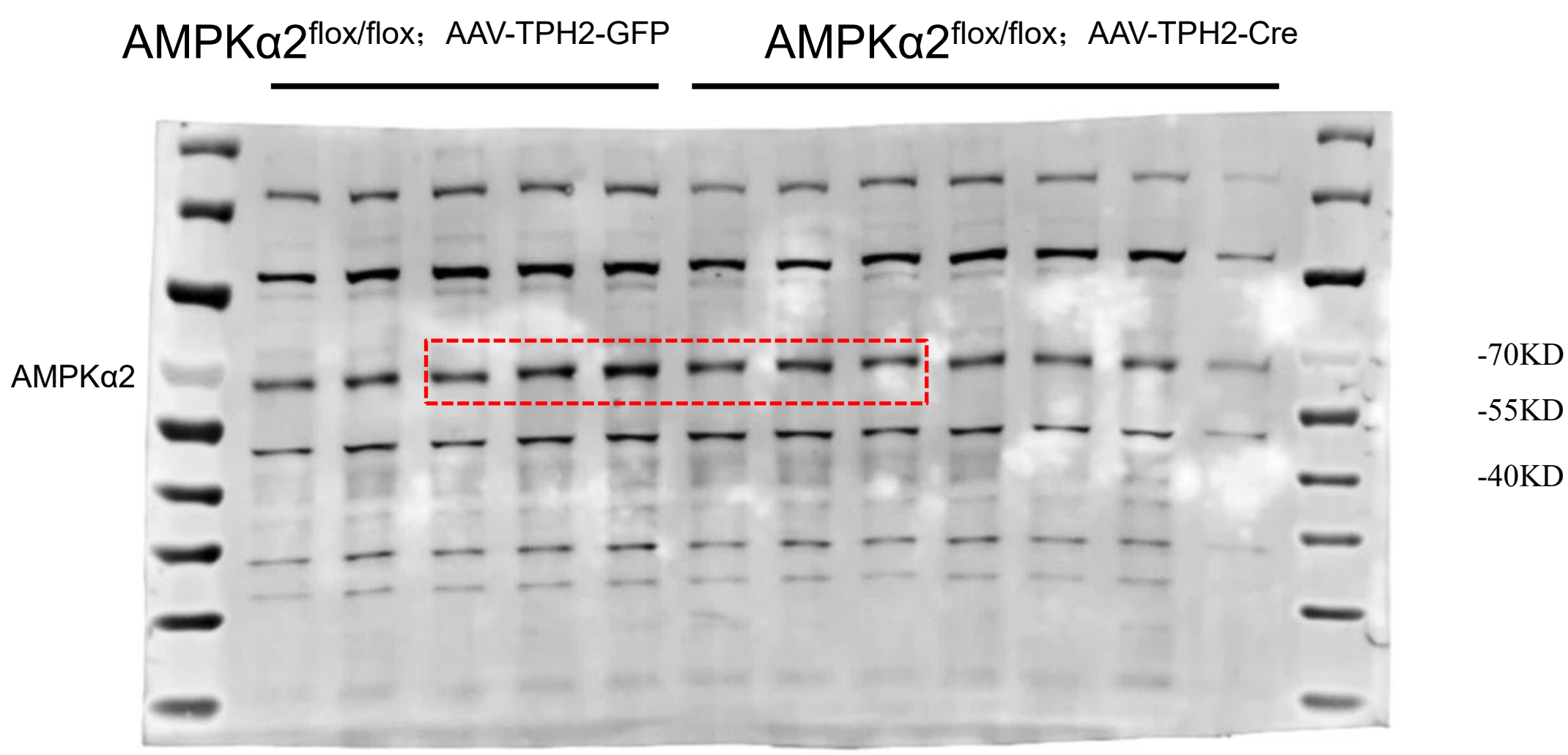
AMPKα2flox/flox; AAV-TPH2-GFP
AMPKα2flox/flox; AAV-TPH2-Cre
AMPKα2
-70KD
-55KD
-40KD

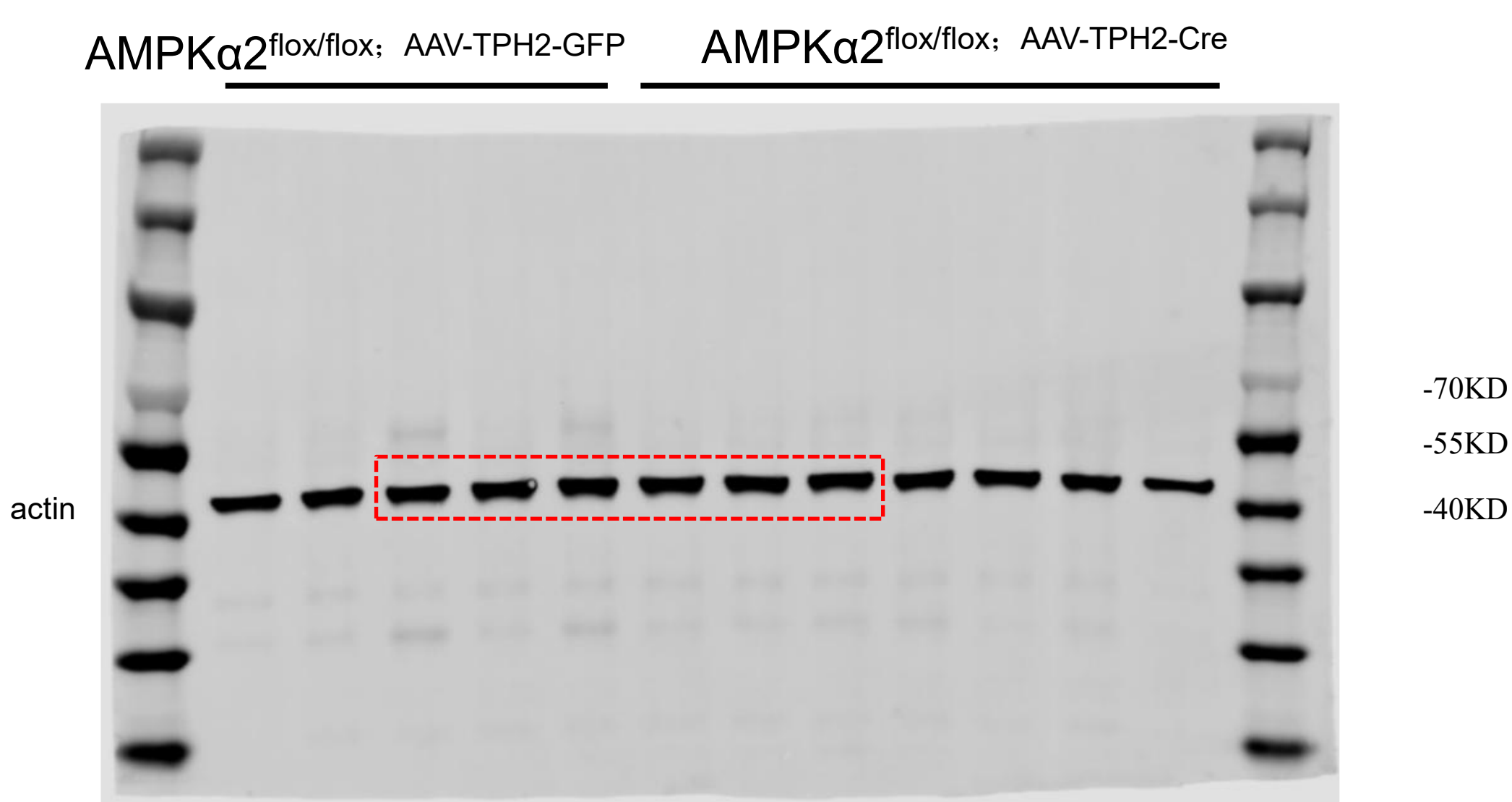
AMPKα2flox/flox; AAV-TPH2-GFP
AMPKα2flox/flox; AAV-TPH2-Cre
actin
-70KD
-55KD
-40KD

Figure 6C

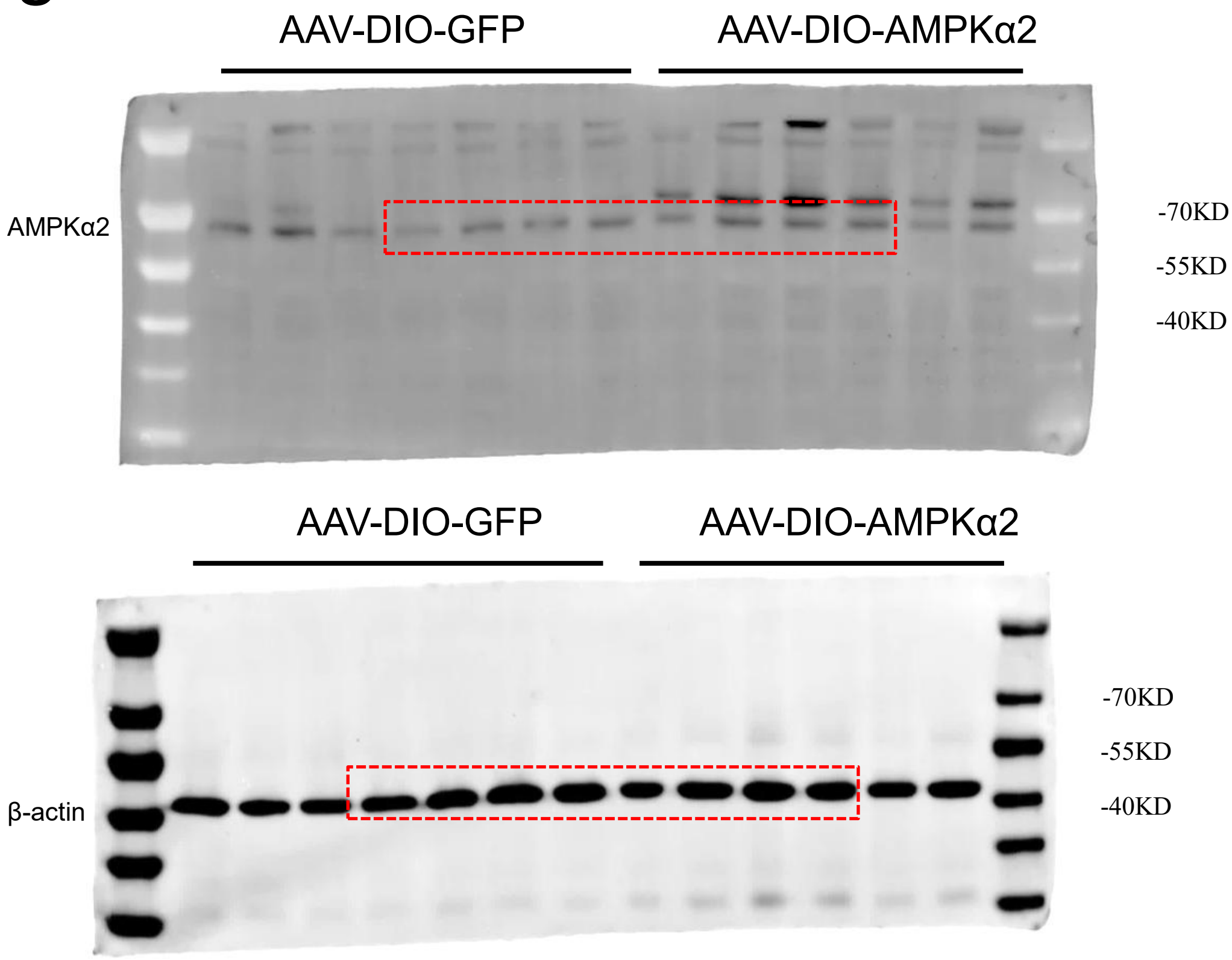

Figure 7D

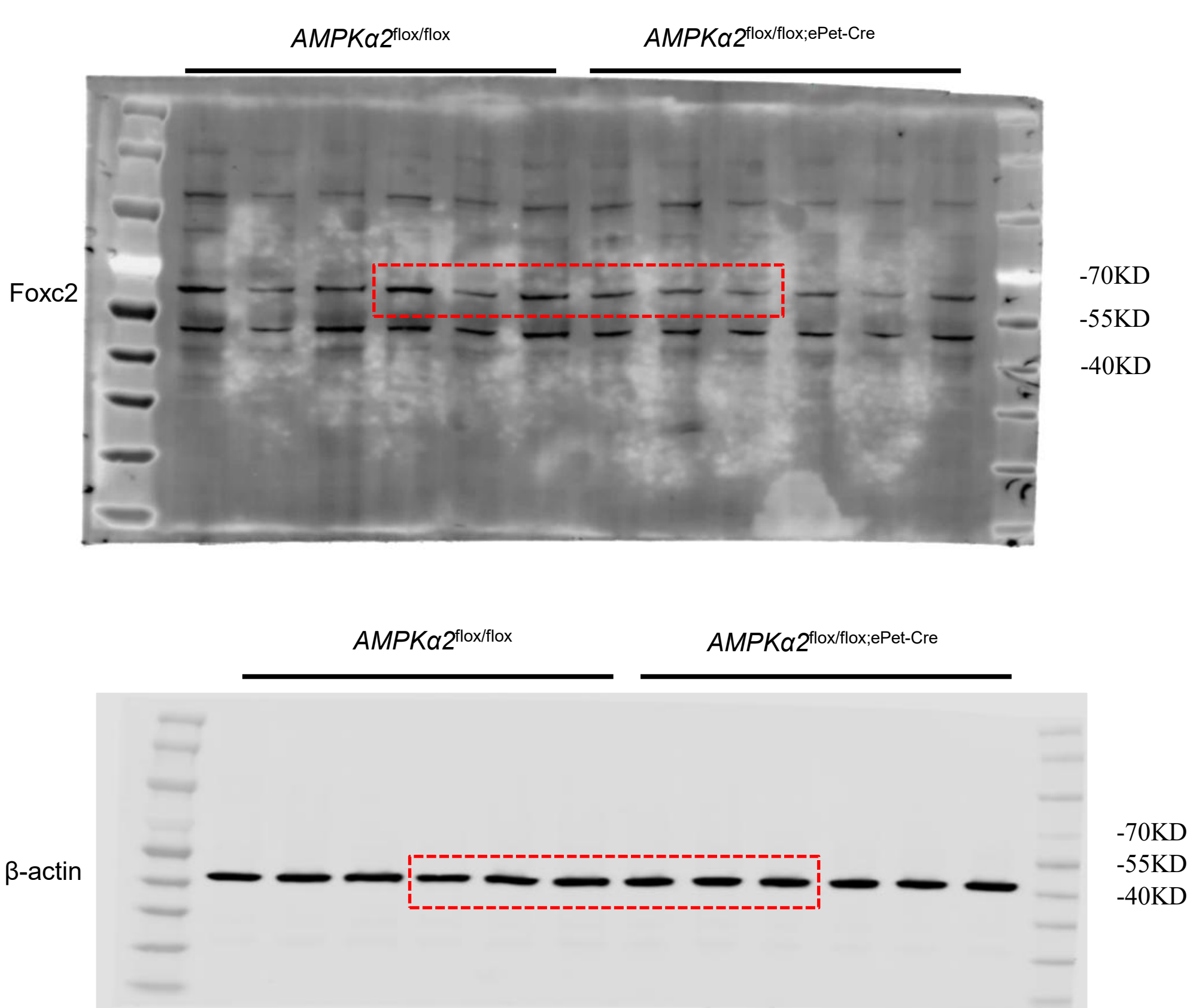

Figure 7I

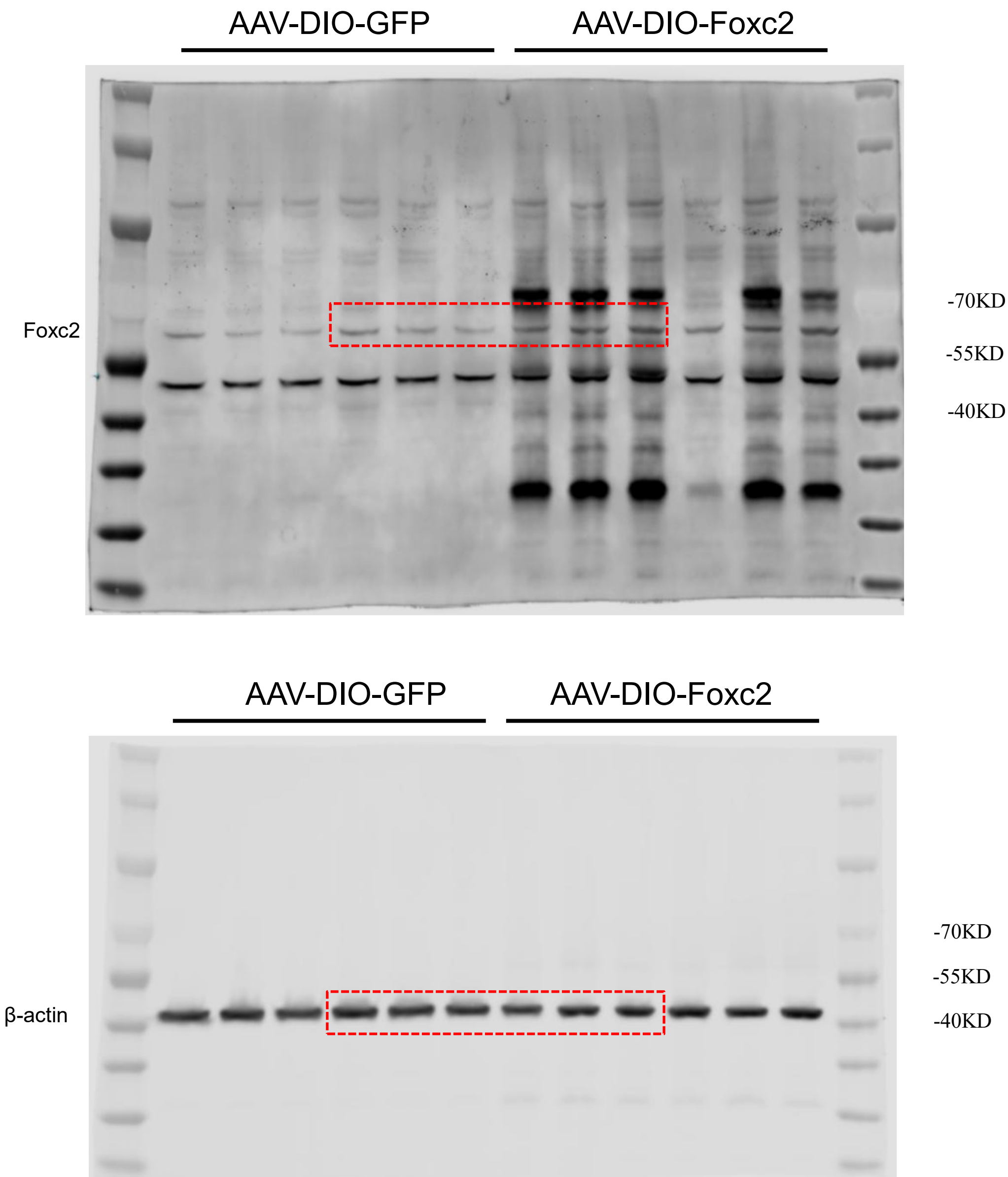

AAV-DIO-GFP
AAV-DIO-Foxc2
Foxc2
-70KD
-55KD
-40KD
AAV-DIO-GFP
AAV-DIO-Foxc2
β-actin
-70KD
-55KD
-40KD

Figure 8C

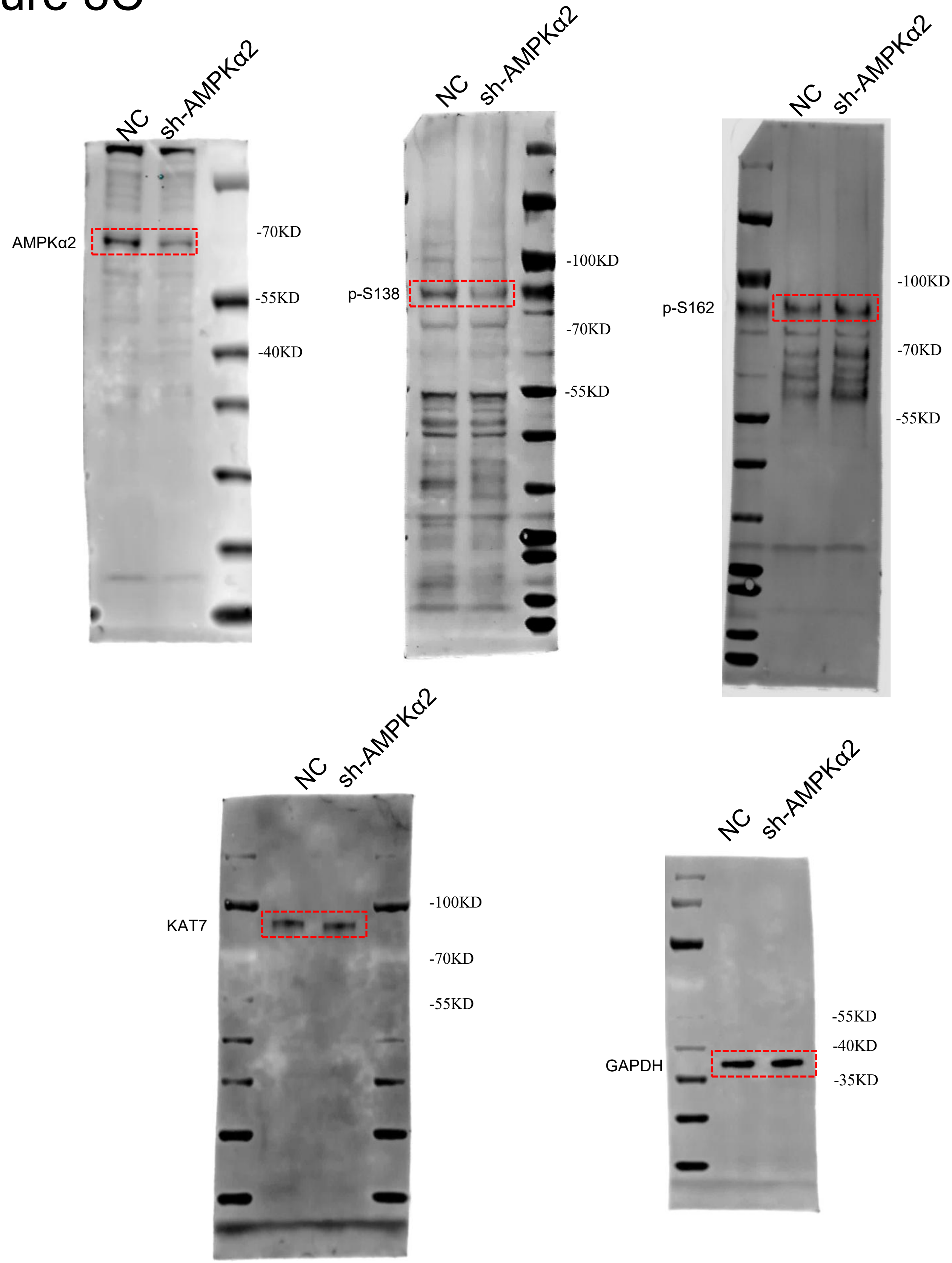
NC
sh-AMPKα2
AMPKα2
-70KD
-55KD
-40KD
p-S138
-100KD
-70KD
-55KD
p-S162
-100KD
-70KD
-55KD
KAT7
-100KD
-70KD
-55KD
GAPDH
-55KD
-40KD
-35KD

Figure 8E

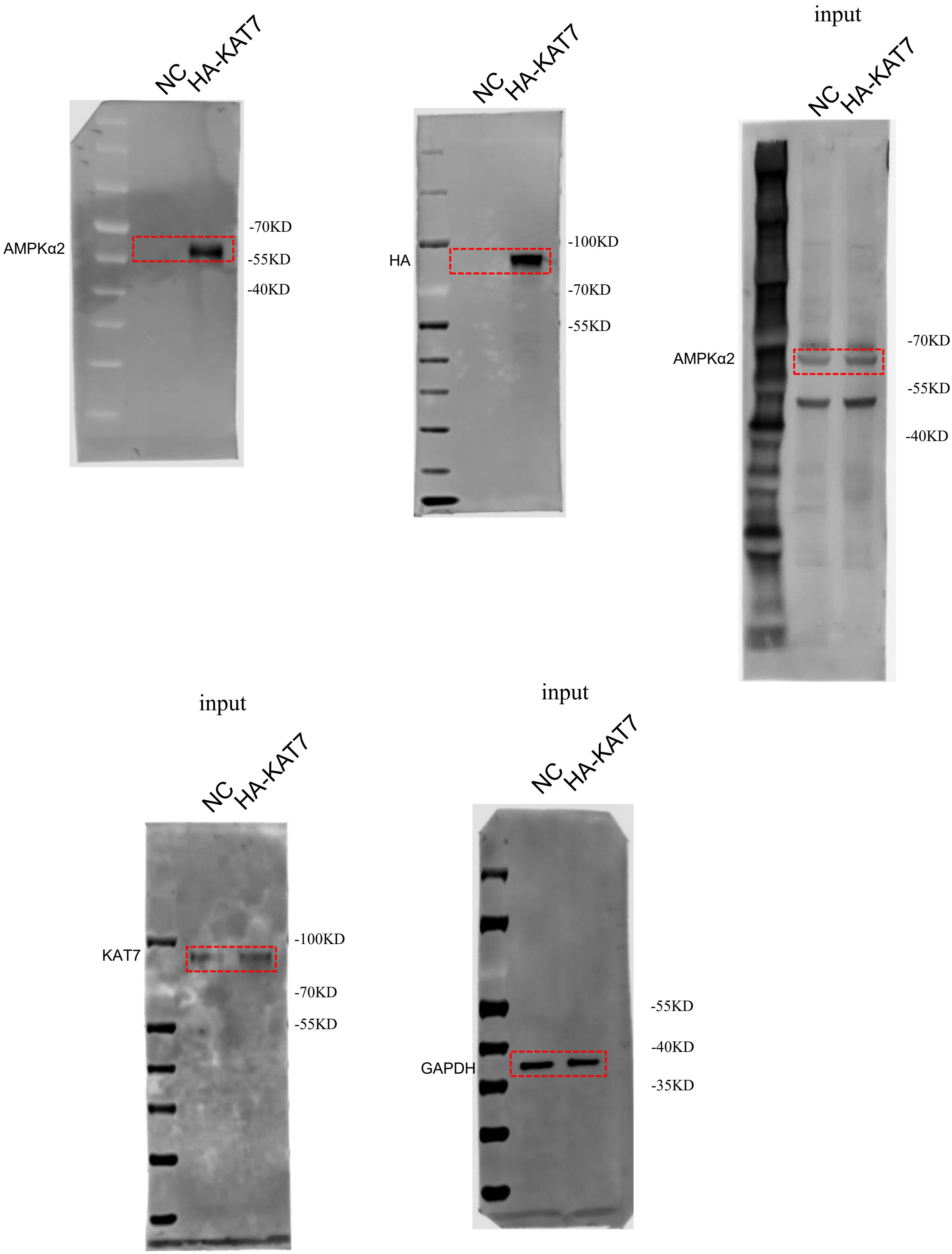

NC
HA-KAT7
AMPKα2
-70KD
-55KD
-40KD
NC
HA-KAT7
HA
-100KD
-70KD
-55KD
input
NC
HA-KAT7
AMPKα2
-70KD
-55KD
-40KD
input
NC
HA-KAT7
KAT7
-100KD
-70KD
-55KD
input
NC
HA-KAT7
GAPDH
-55KD
-40KD
-35KD

Figure 8F

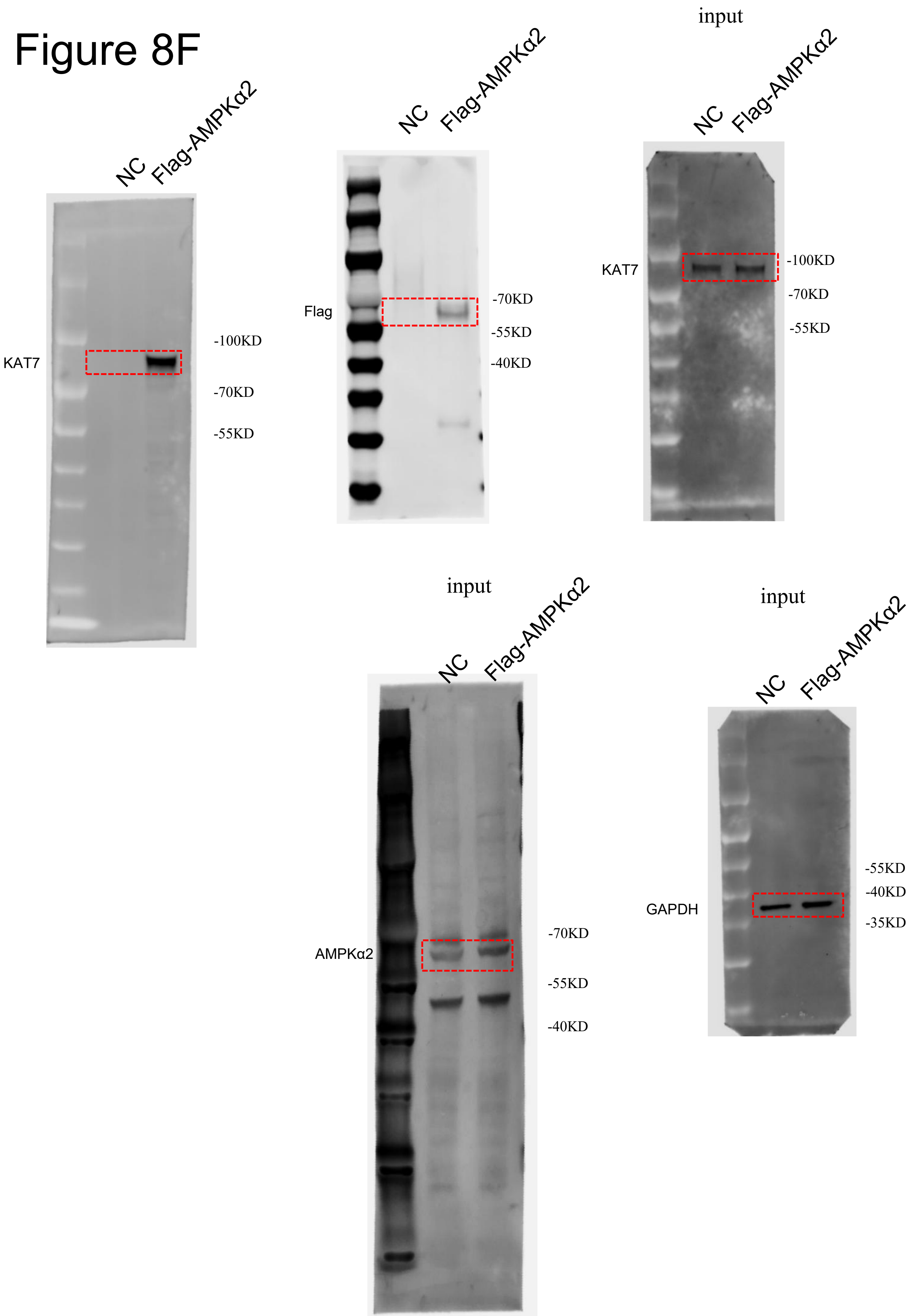
NC
Flag-AMPKα2
KAT7
-100KD
-70KD
-55KD
NC
Flag-AMPKα2
Flag
-70KD
-55KD
-40KD
input
NC
Flag-AMPKα2
KAT7
-100KD
-70KD
-55KD
input
NC
Flag-AMPKα2
AMPKα2
-70KD
-55KD
-40KD
input
NC
Flag-AMPKα2
GAPDH
-55KD
-40KD
-35KD

Figure 8G

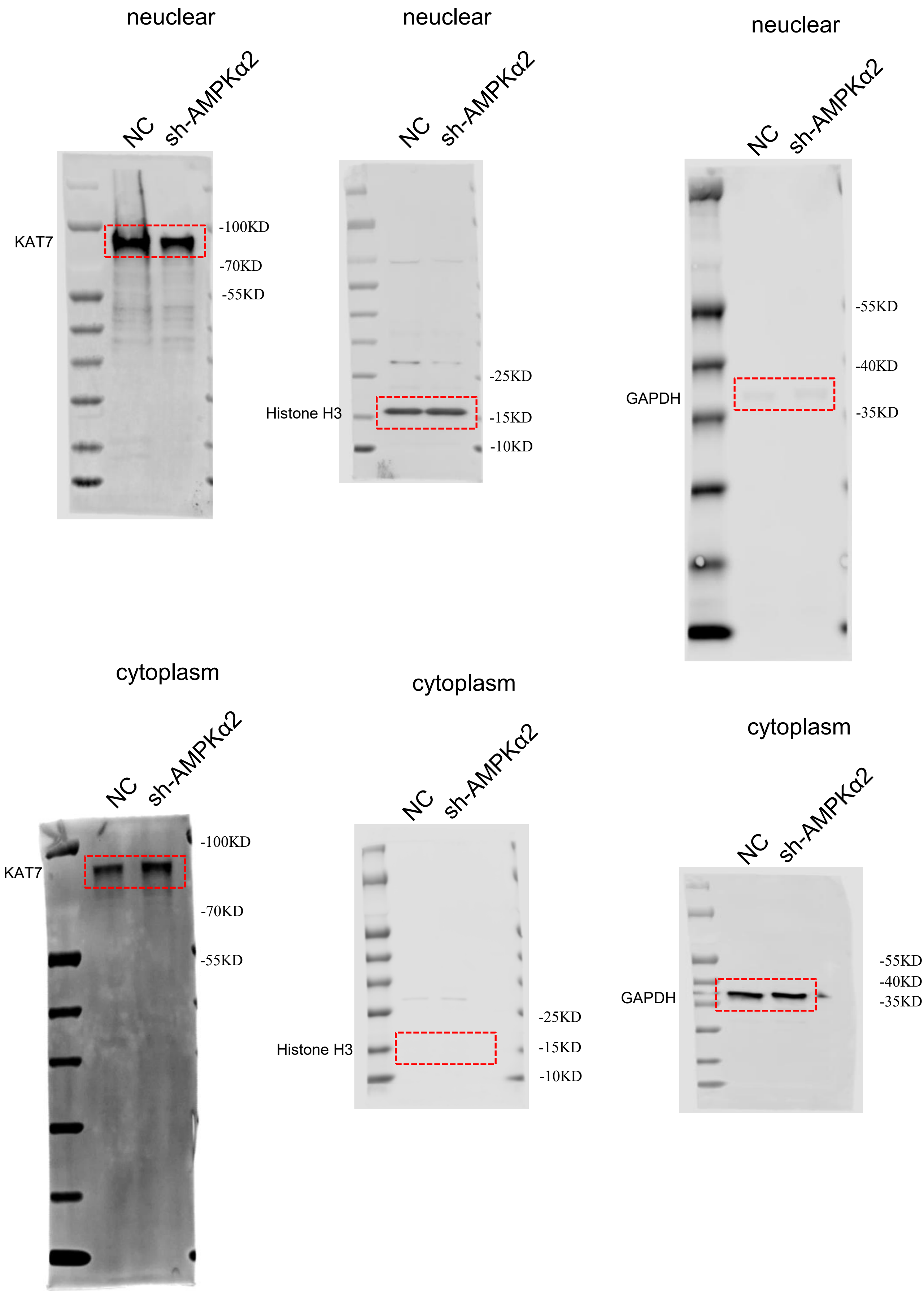
neuclear
NC
sh-AMPKα2
KAT7
-100KD
-70KD
-55KD
neuclear
NC
sh-AMPKα2
Histone H3
-25KD
-15KD
-10KD
neuclear
NC
sh-AMPKα2
GAPDH
-55KD
-40KD
-35KD
cytoplasm
NC
sh-AMPKα2
KAT7
-100KD
-70KD
-55KD
cytoplasm
NC
sh-AMPKα2
Histone H3
-25KD
-15KD
-10KD
cytoplasm
NC
sh-AMPKα2
GAPDH
-55KD
-40KD
-35KD

Figure 8H

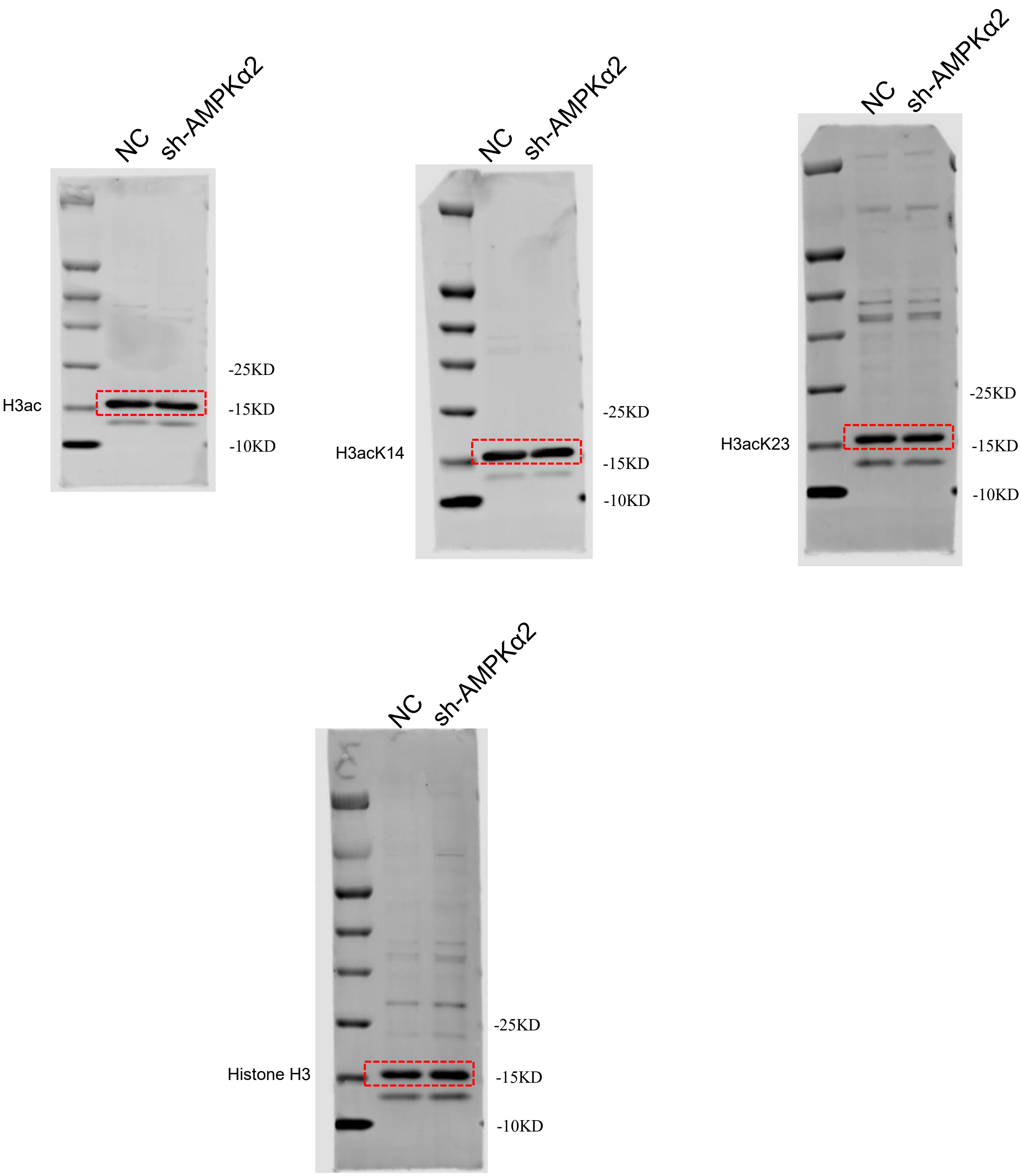

NC
sh-AMPKα2
H3ac
-25KD
-15KD
-10KD
NC
sh-AMPKα2
H3acK14
-25KD
-15KD
-10KD
NC
sh-AMPKα2
H3acK23
-25KD
-15KD
-10KD
NC
sh-AMPKα2
Histone H3
-25KD
-15KD
-10KD

Figure 8H

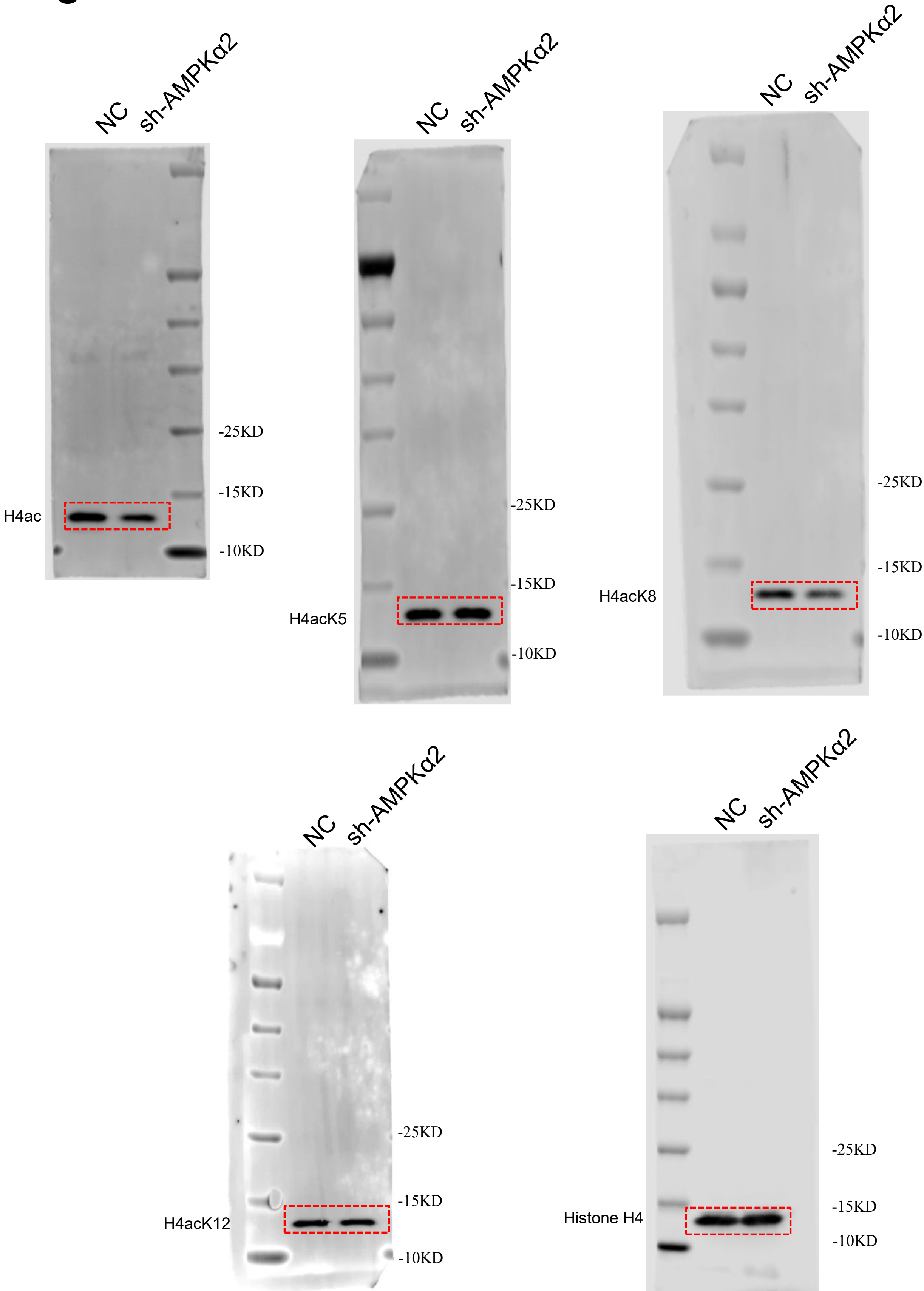
NC
sh-AMPKα2
H4ac
-25KD
-15KD
-10KD
NC
sh-AMPKα2
H4acK5
-25KD
-15KD
-10KD
NC
sh-AMPKα2
H4acK8
-25KD
-15KD
-10KD
NC
sh-AMPKα2
H4acK12
-25KD
-15KD
-10KD
NC
sh-AMPKα2
Histone H4
-25KD
-15KD
-10KD

Figure 8J

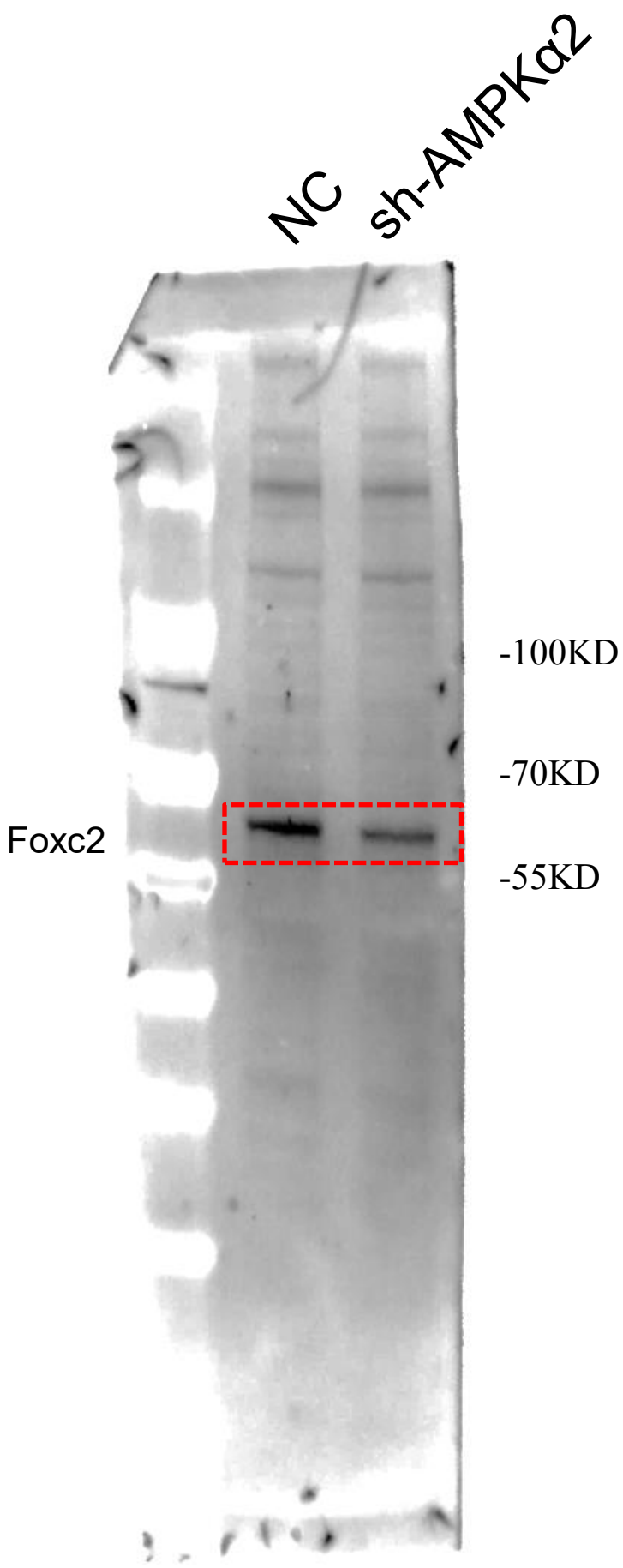
NC
sh-AMPKα2
-100KD
-70KD
Foxc2
-55KD

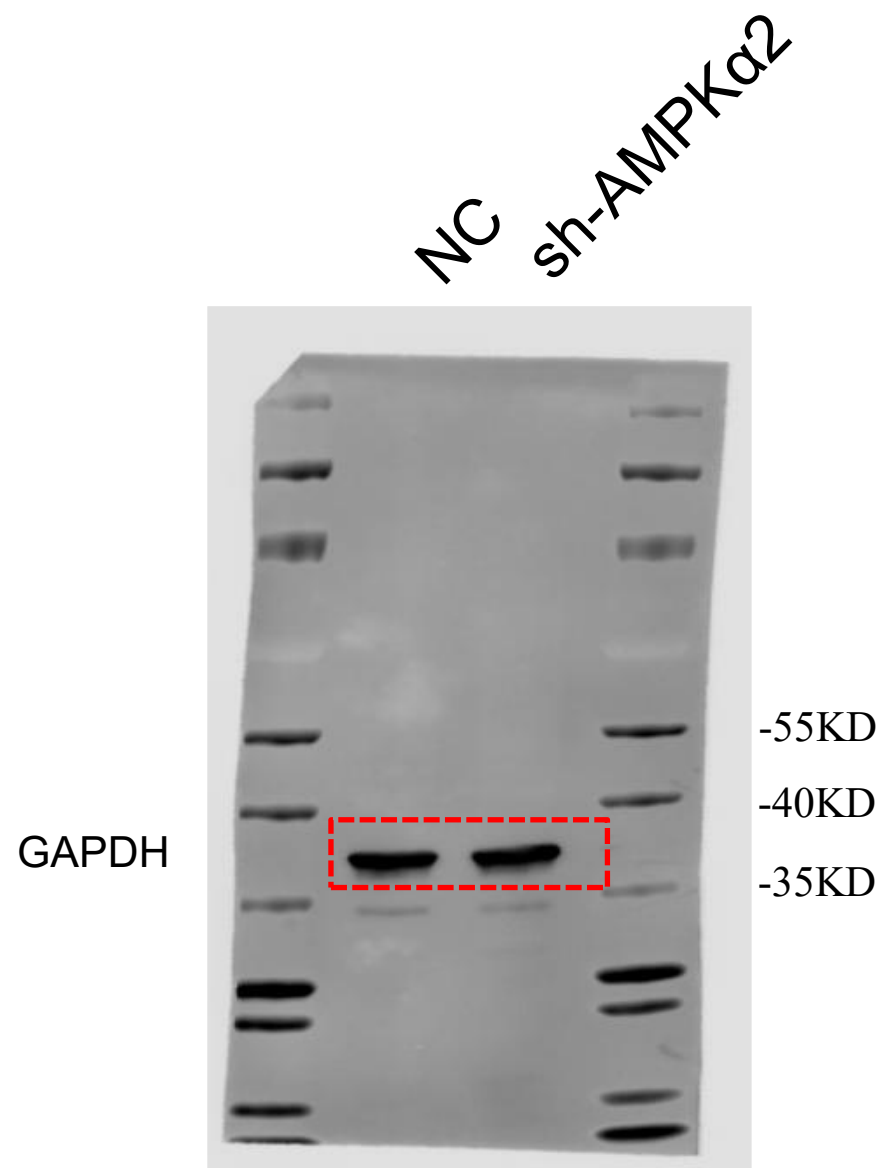
NC
sh-AMPKα2
-55KD
-40KD
GAPDH
-35KD

Figure 8J

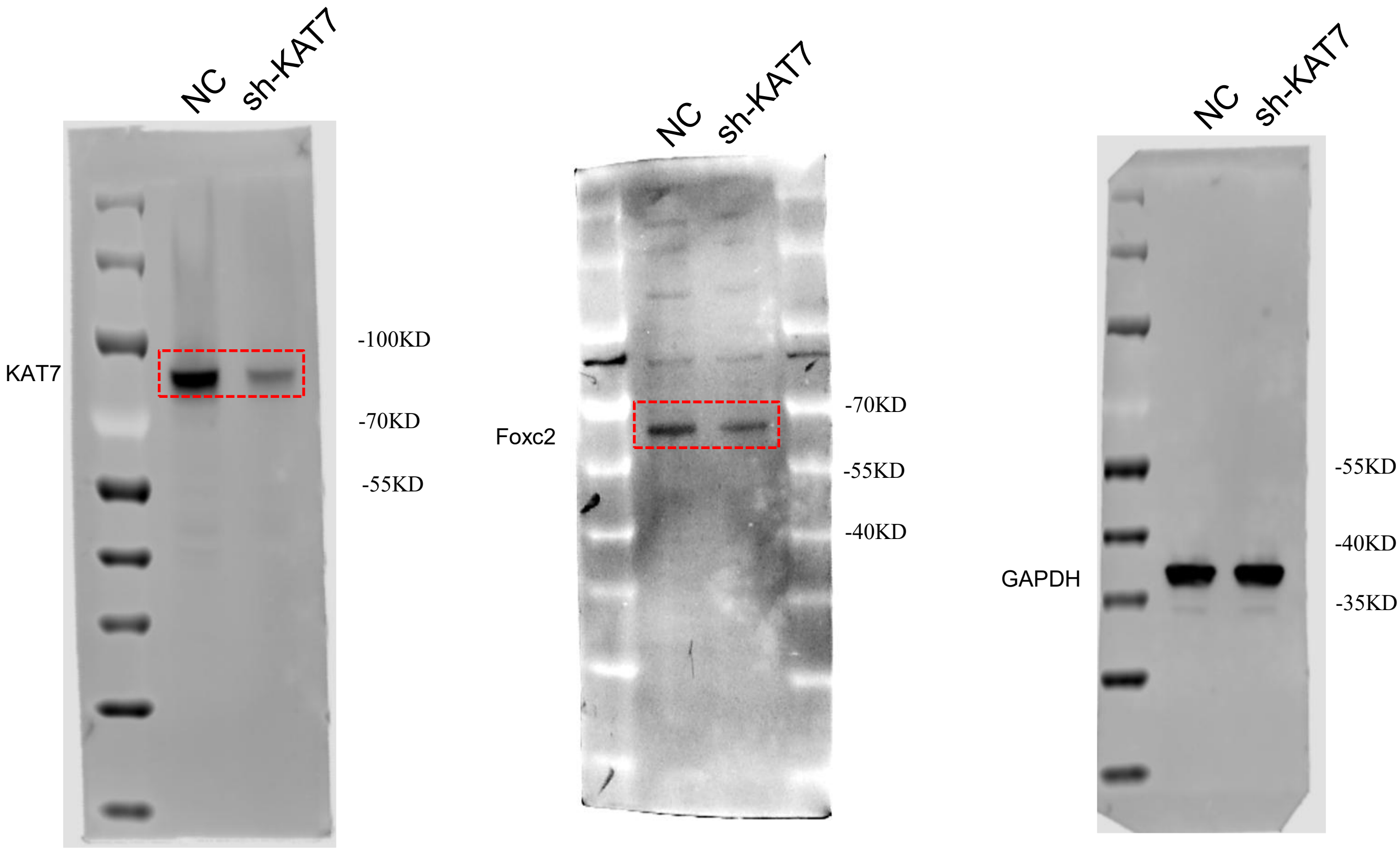

NC
sh-KAT7
KAT7
-100KD
-70KD
-55KD
NC
sh-KAT7
Foxc2
-70KD
-55KD
-40KD
NC
sh-KAT7
GAPDH
-55KD
-40KD
-35KD

Figure 8M

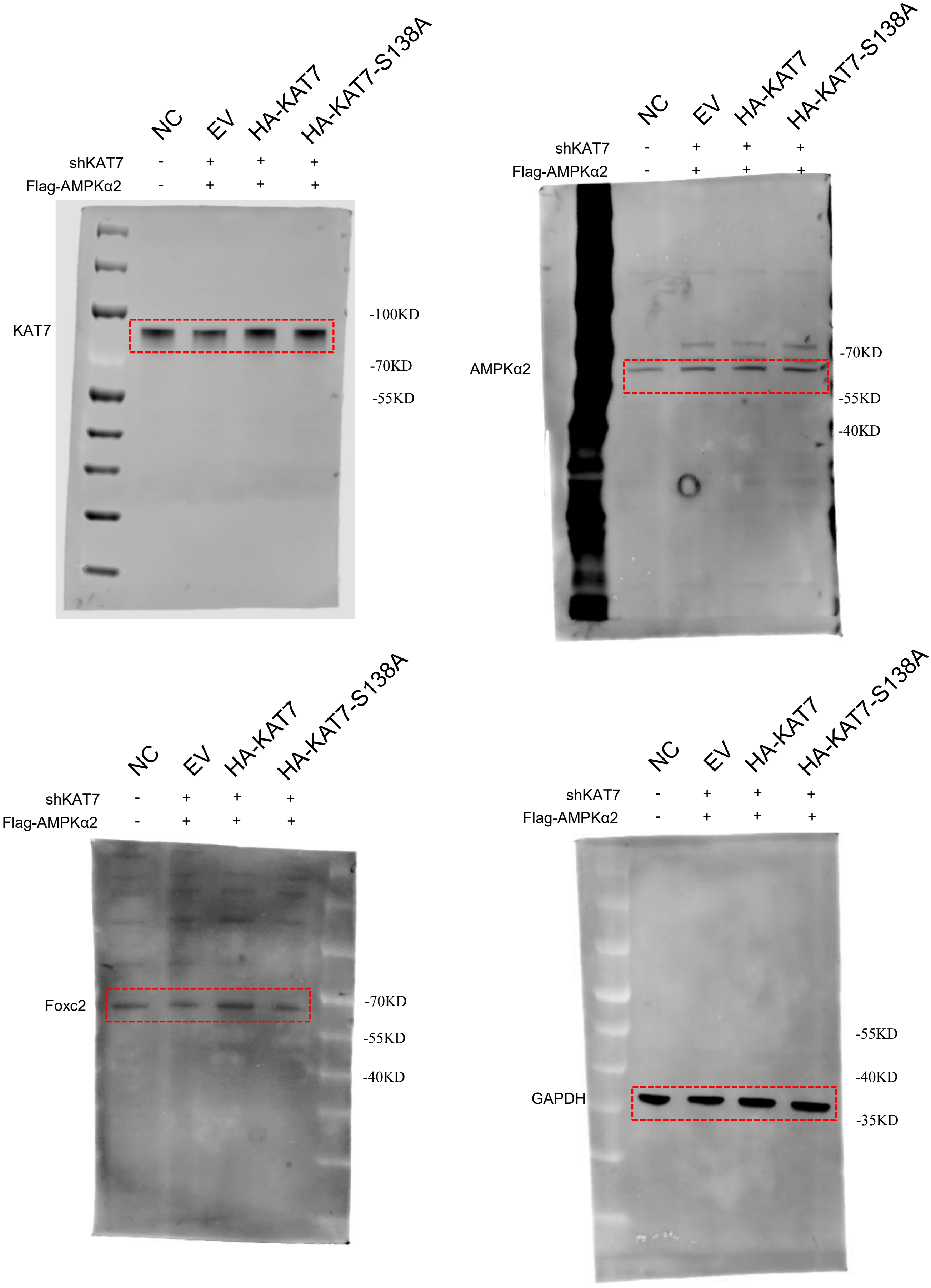
NC
EV
HA-KAT7
HA-KAT7-S138A
shKAT7 - + + +
Flag-AMPKα2 - + + +
KAT7
-100KD
-70KD
-55KD
AMPKα2
-70KD
-55KD
-40KD
Foxc2
-70KD
-55KD
-40KD
GAPDH
-55KD
-40KD
-35KD

Figure S5A

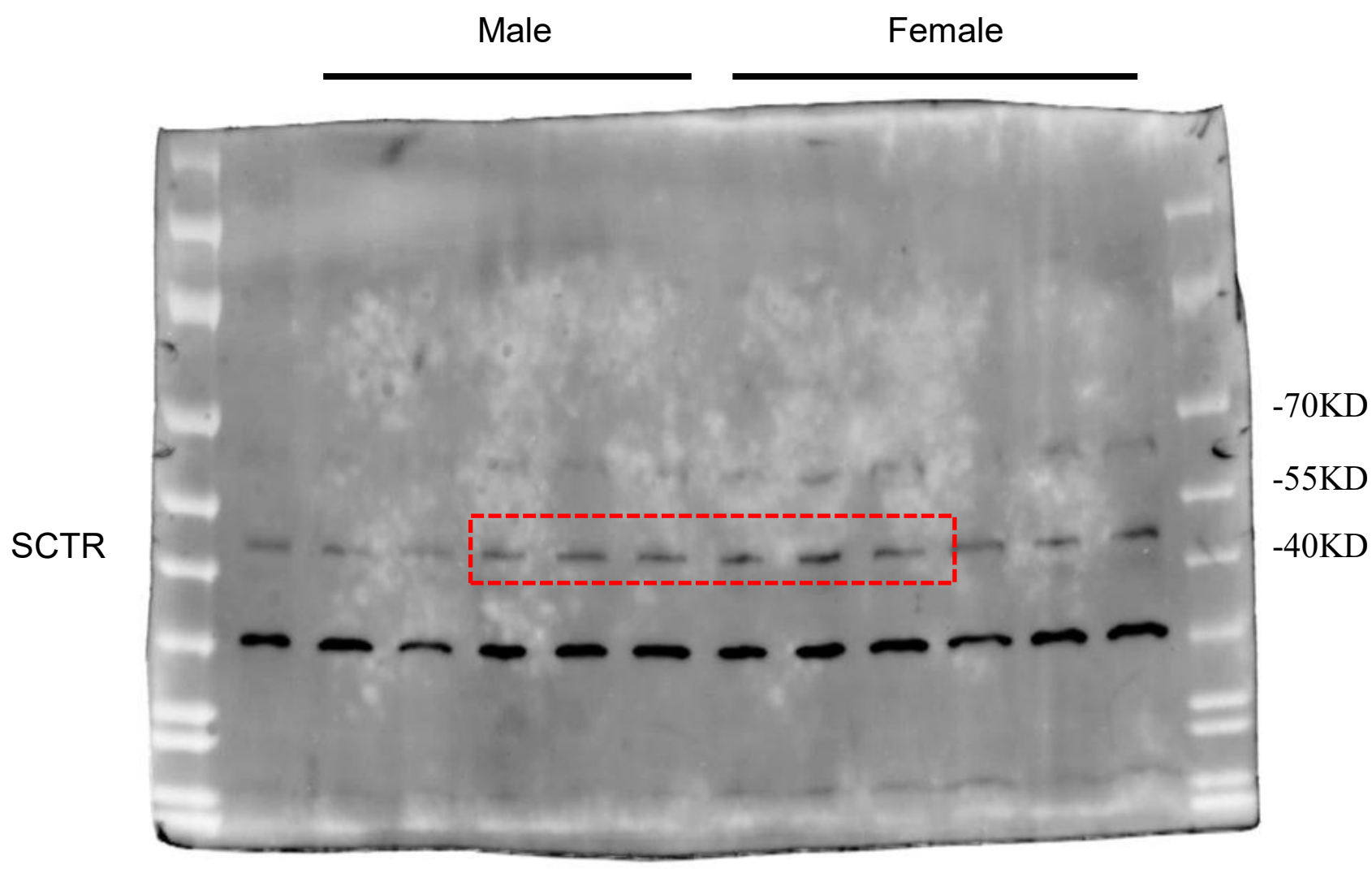
Male
Female
SCTR
-70KD
-55KD
-40KD

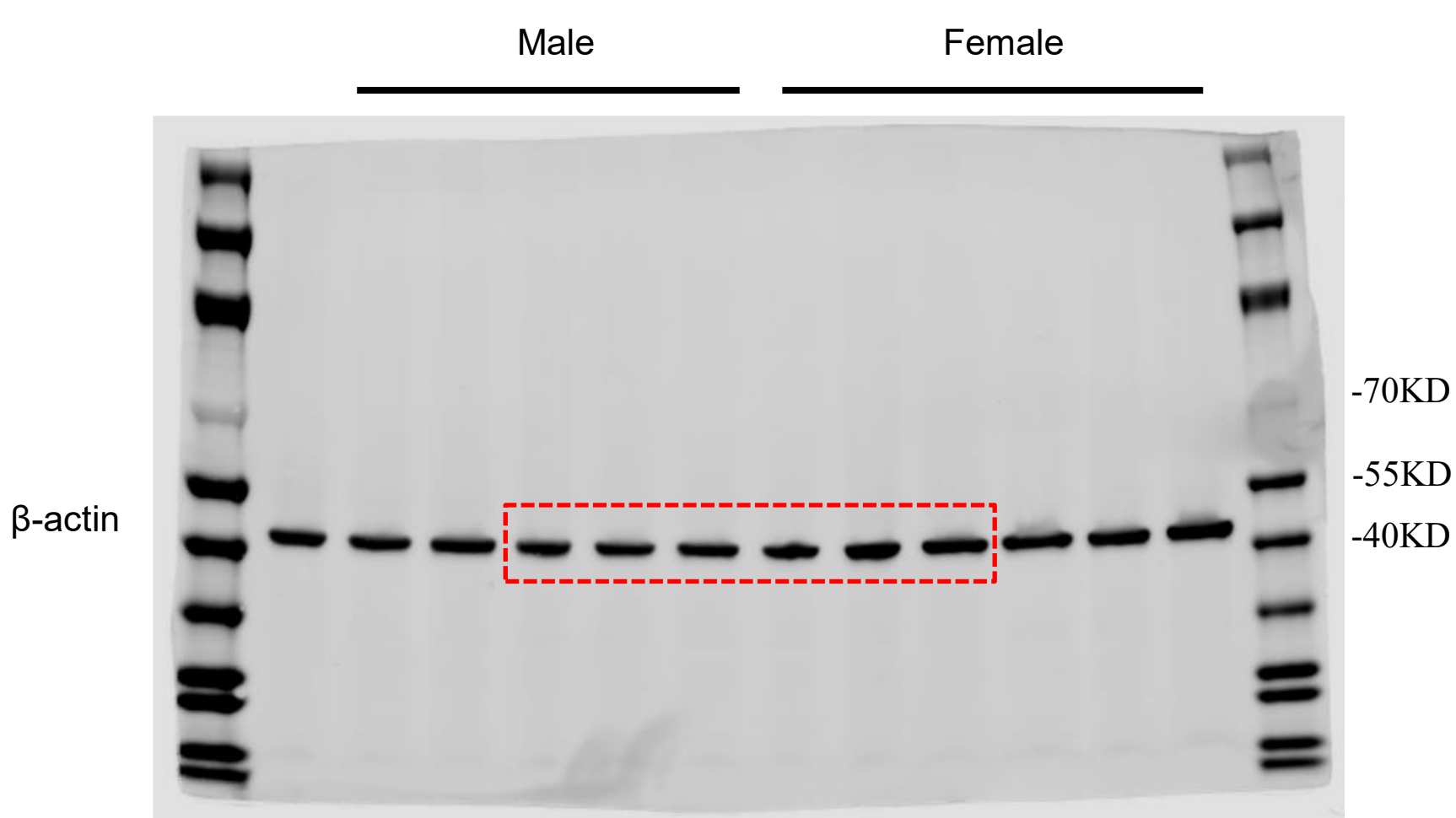
Male
Female
β-actin
-70KD
-55KD
-40KD

Figure S8A

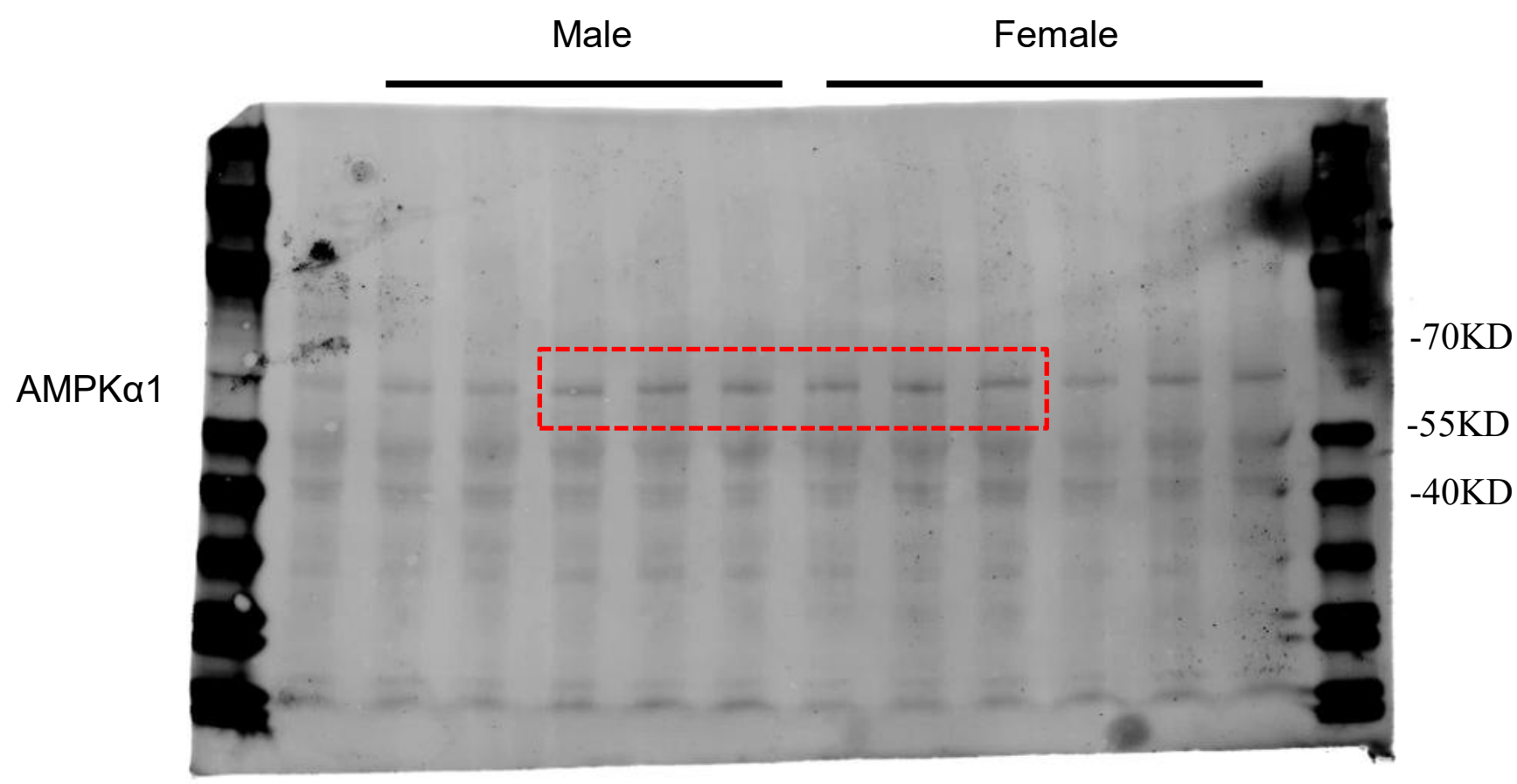
Male
Female
AMPKα1
-70KD
-55KD
-40KD

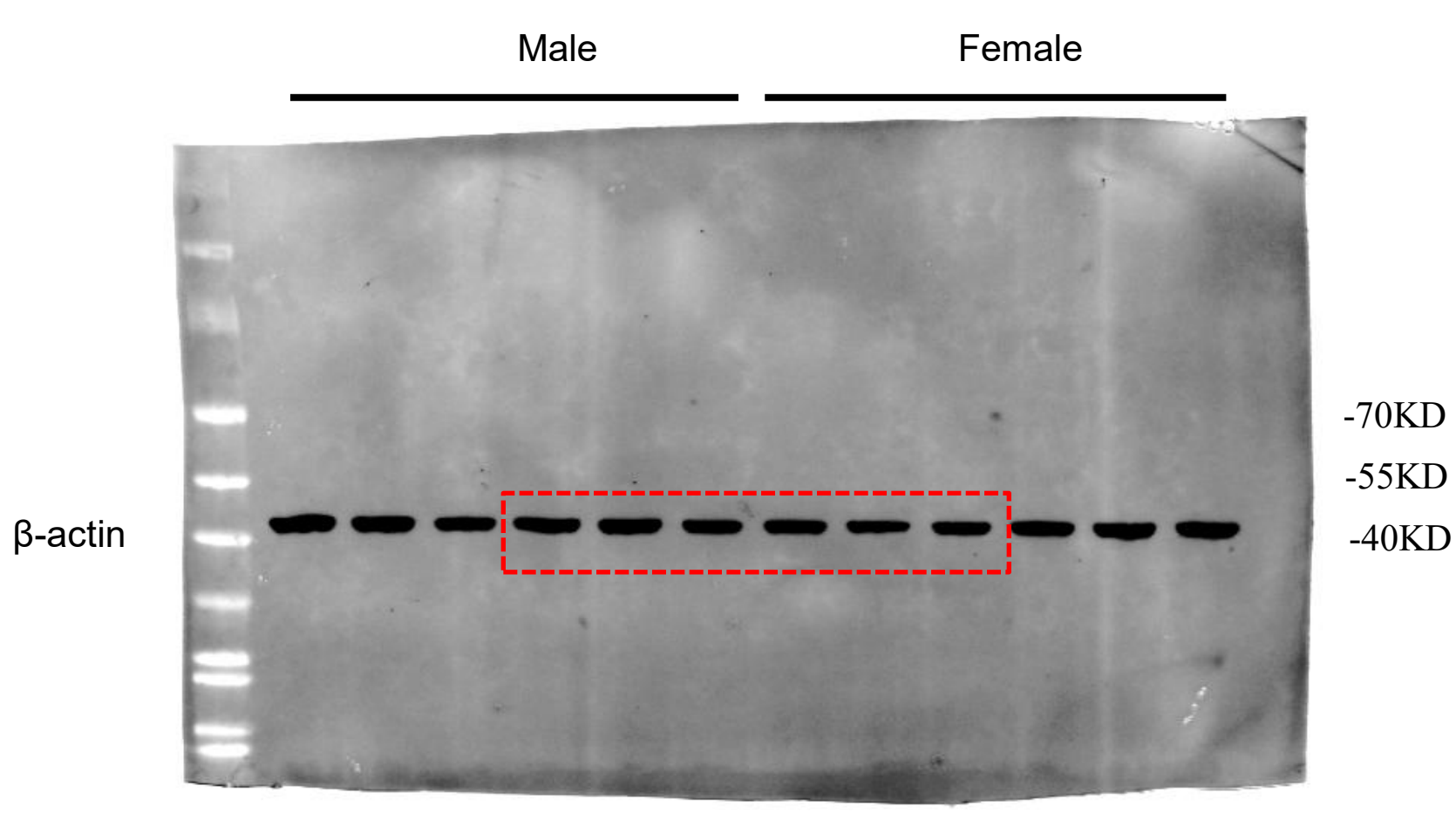
Male
Female
β-actin
-70KD
-55KD
-40KD

Figure S11A

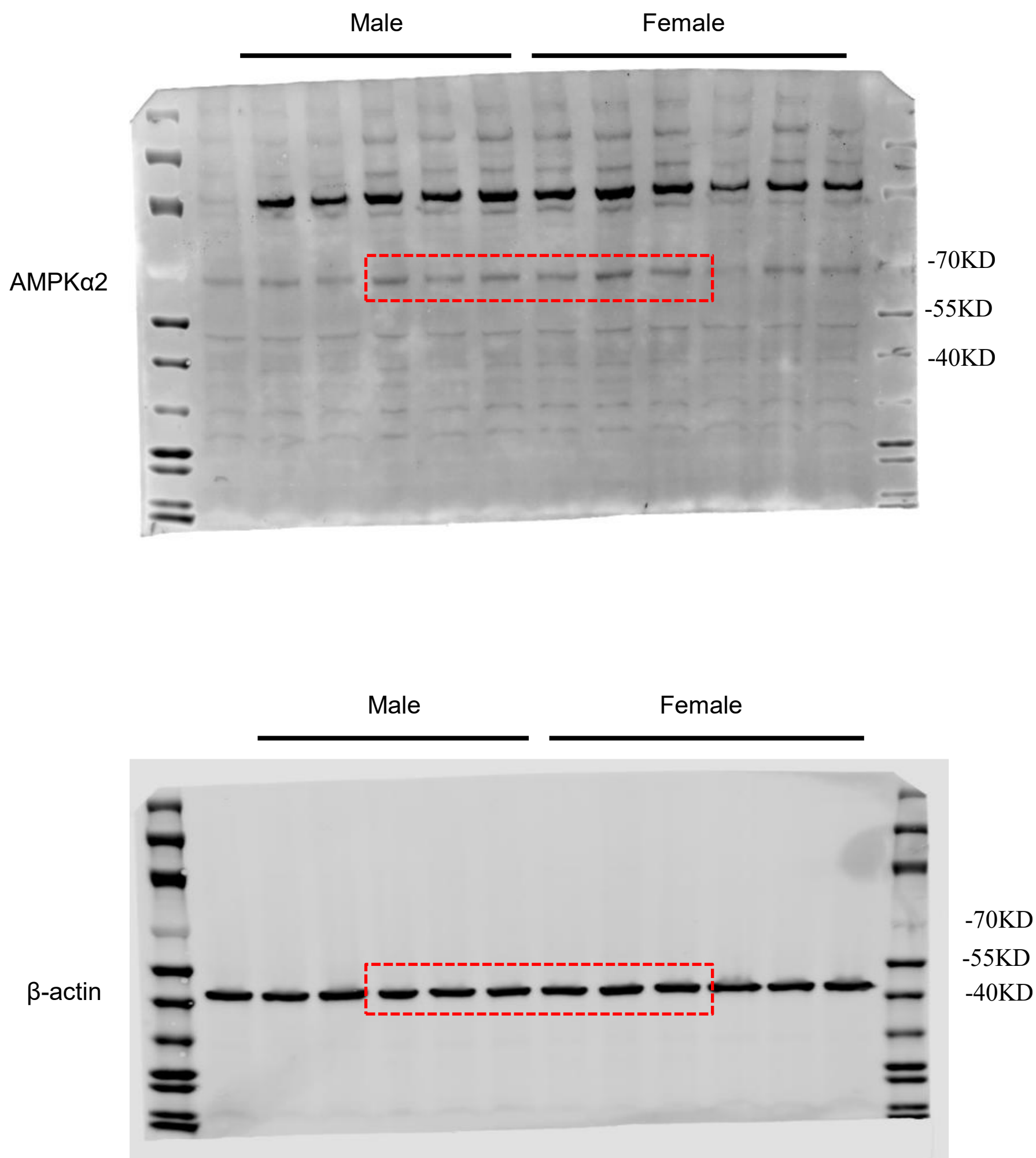
Male
Female
AMPKα2
-70KD
-55KD
-40KD
Male
Female
β-actin
-70KD
-55KD
-40KD

Figure S15C

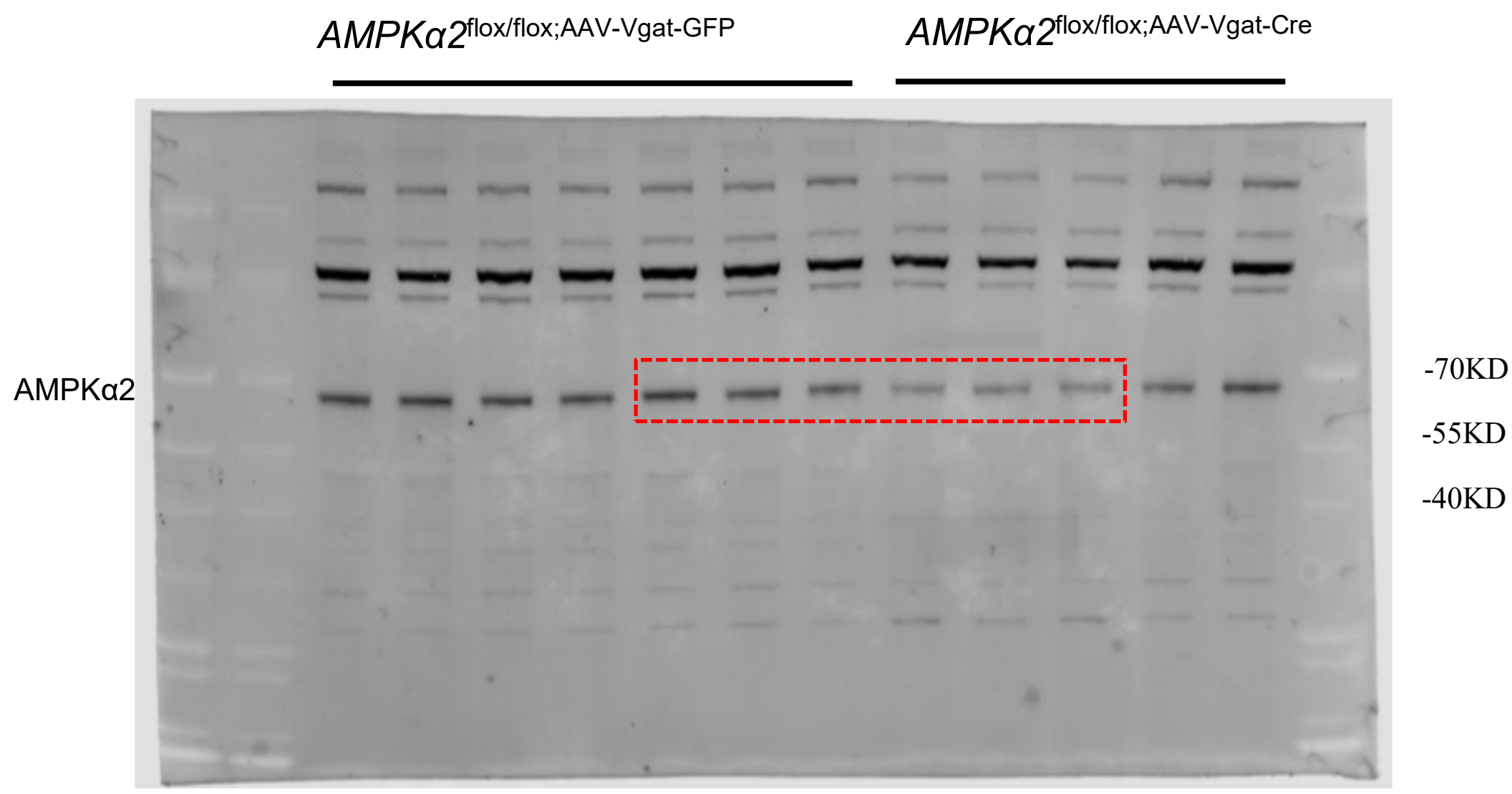

AMPKα2flox/flox;AAV-Vgat-GFP
AMPKα2flox/flox;AAV-Vgat-Cre
AMPKα2
-70KD
-55KD
-40KD

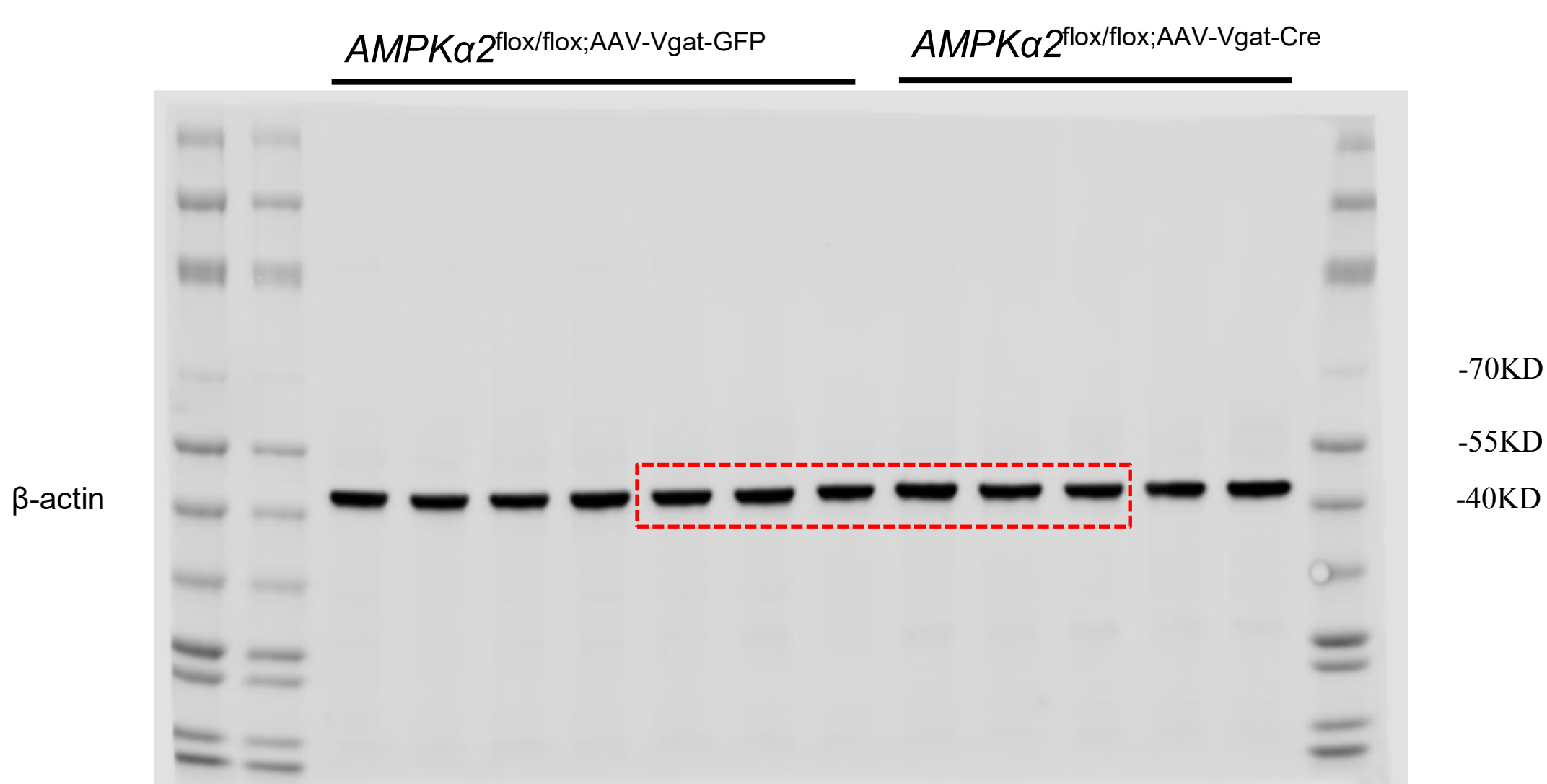

AMPKα2flox/flox;AAV-Vgat-GFP
AMPKα2flox/flox;AAV-Vgat-Cre
β-actin
-70KD
-55KD
-40KD

Figure S17C

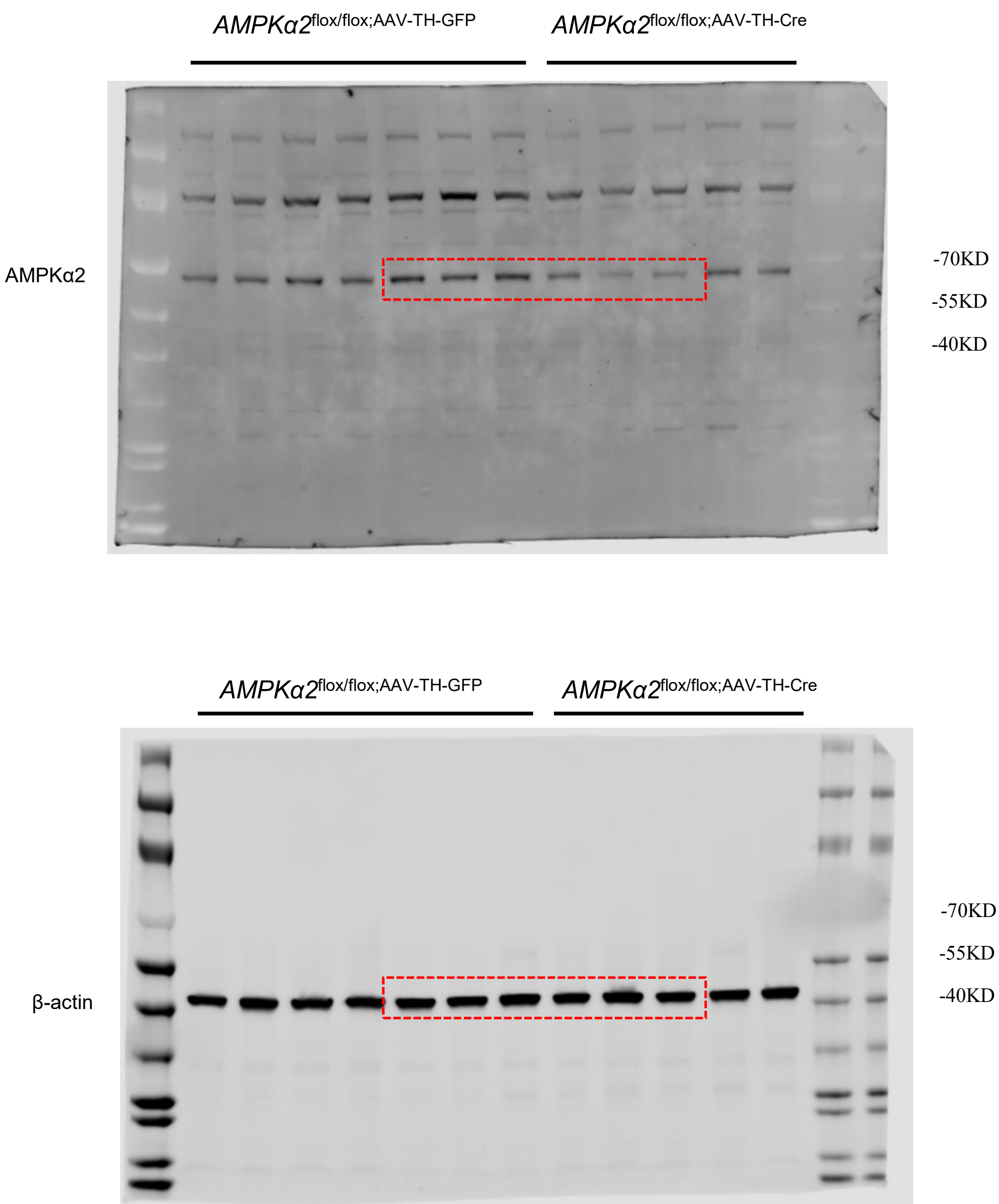

AMPKα2flox/flox;AAV-TH-GFP
AMPKα2flox/flox;AAV-TH-Cre
AMPKα2
-70KD
-55KD
-40KD
AMPKα2flox/flox;AAV-TH-GFP
AMPKα2flox/flox;AAV-TH-Cre
β-actin
-70KD
-55KD
-40KD

Figure S24C

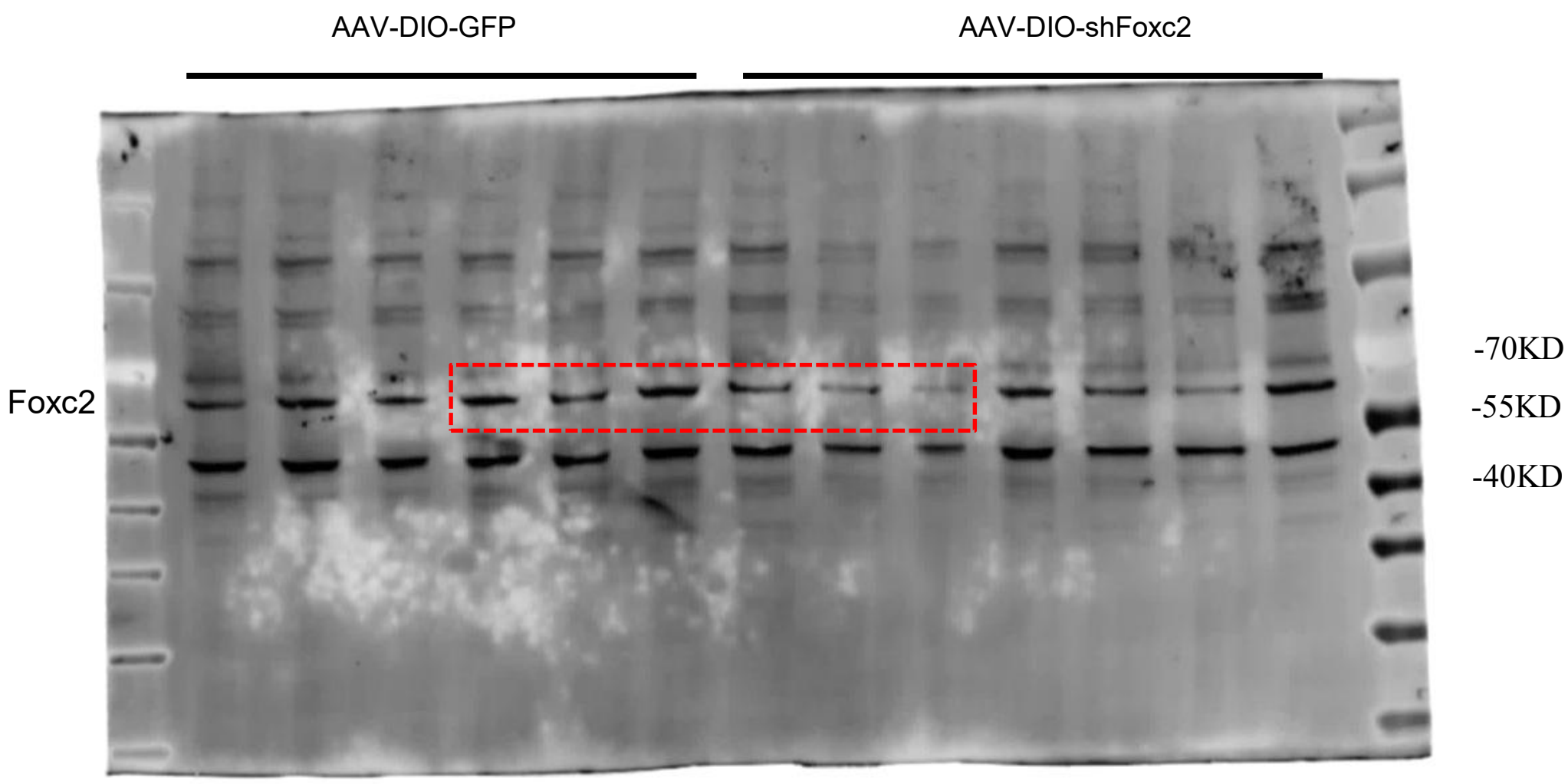
AAV-DIO-GFP
AAV-DIO-shFoxc2
Foxc2
-70KD
-55KD
-40KD

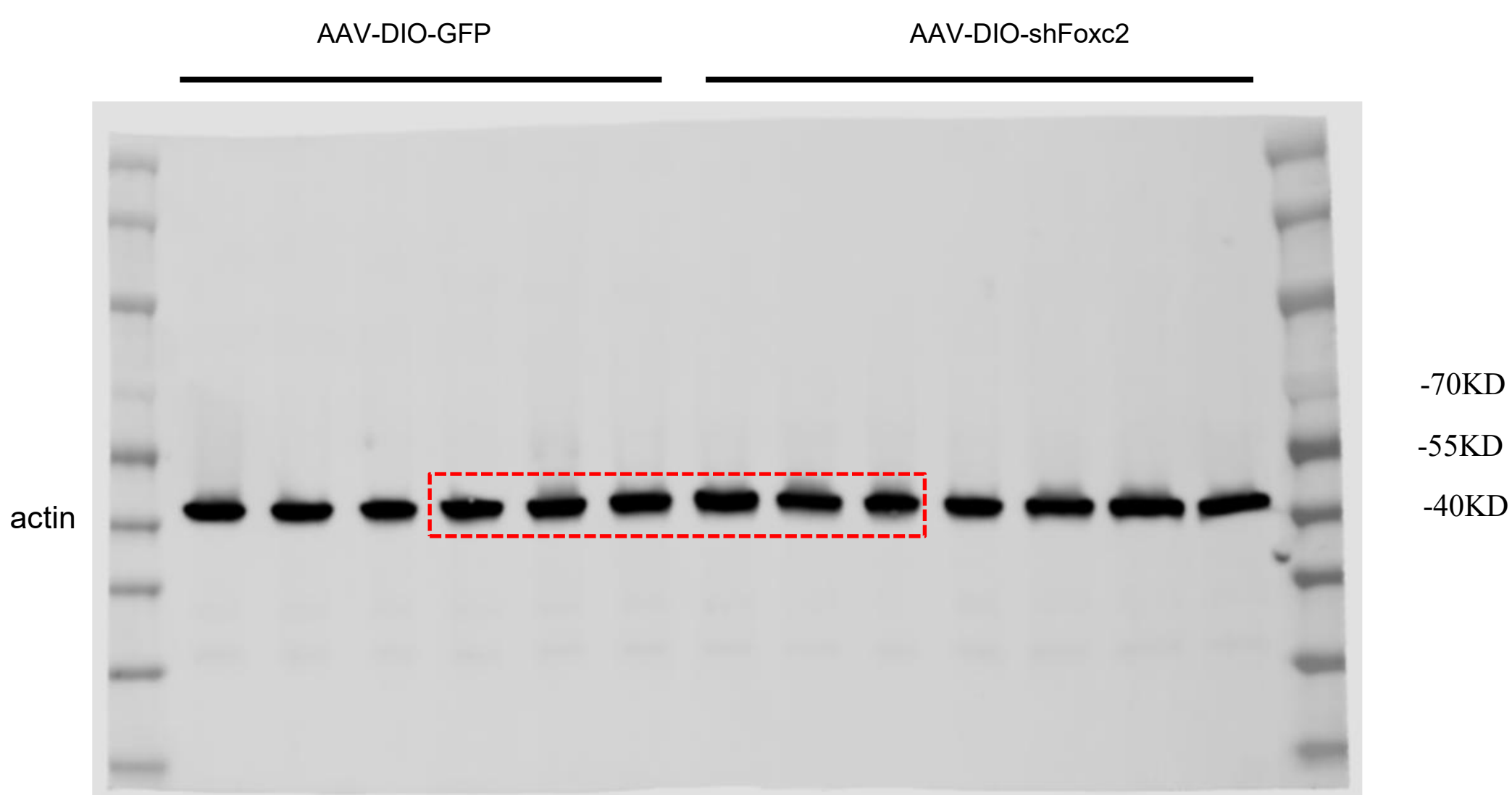
AAV-DIO-GFP
AAV-DIO-shFoxc2
actin
-70KD
-55KD
-40KD

Figure S27B

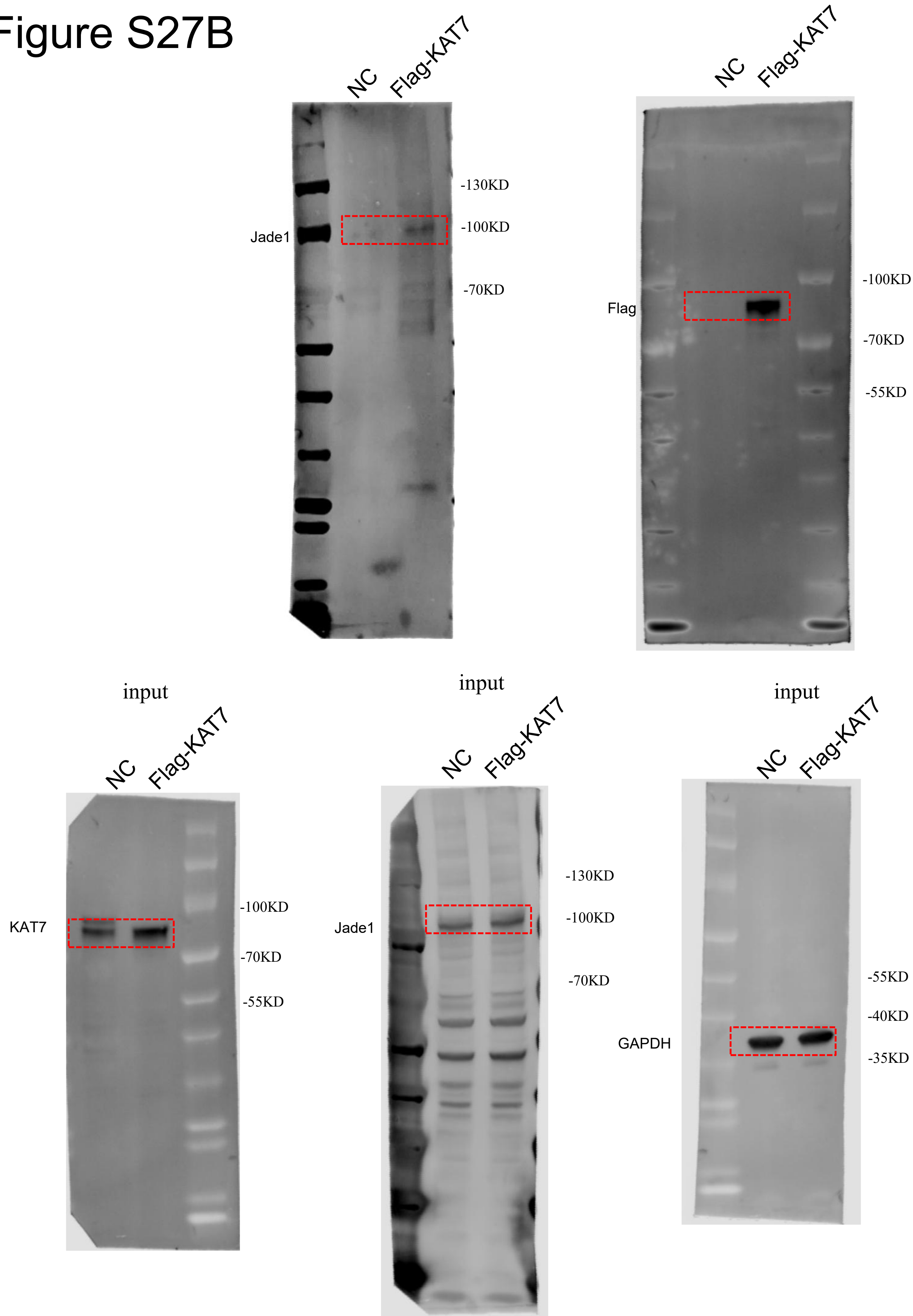
NC
Flag-KAT7
-130KD
-100KD
-70KD
Jade1
NC
Flag-KAT7
-100KD
-70KD
-55KD
Flag
input
NC
Flag-KAT7
-100KD
-70KD
-55KD
KAT7
input
NC
Flag-KAT7
-130KD
-100KD
-70KD
Jade1
input
NC
Flag-KAT7
-55KD
-40KD
-35KD
GAPDH

Figure 2B

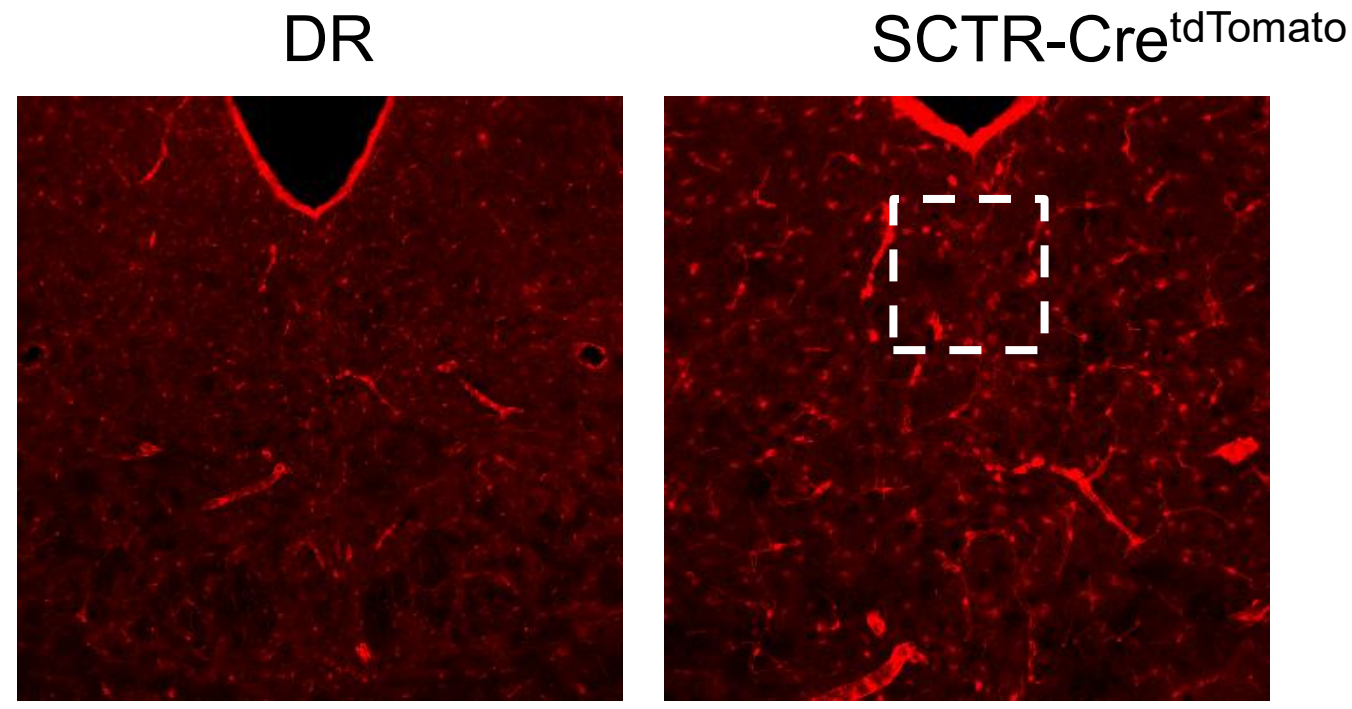

Figure 2C

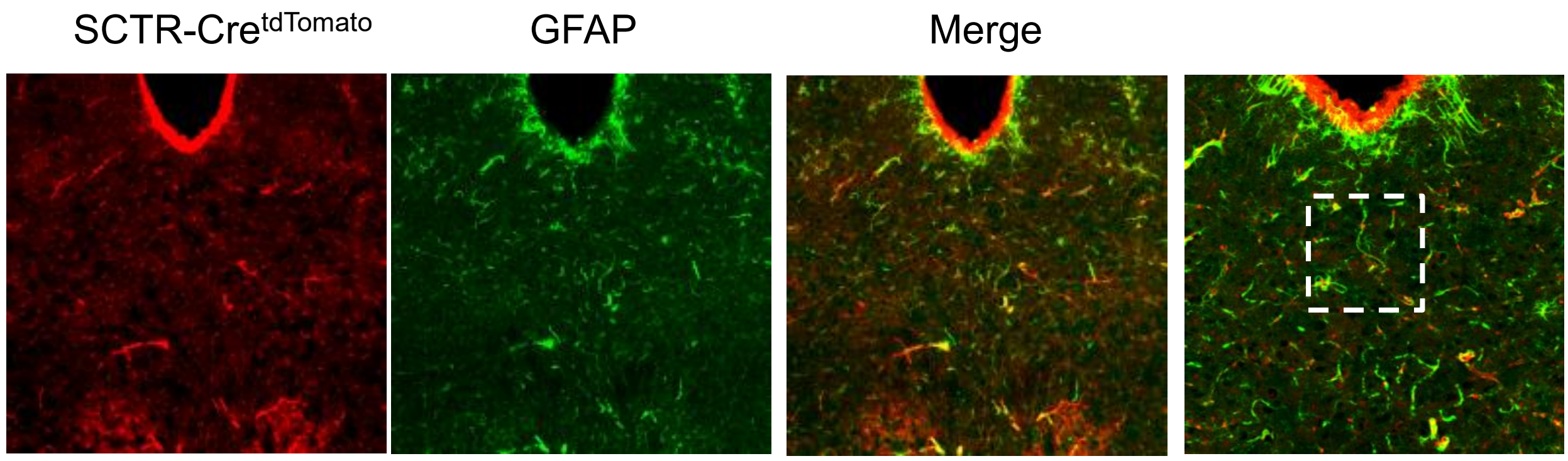

Figure 2E

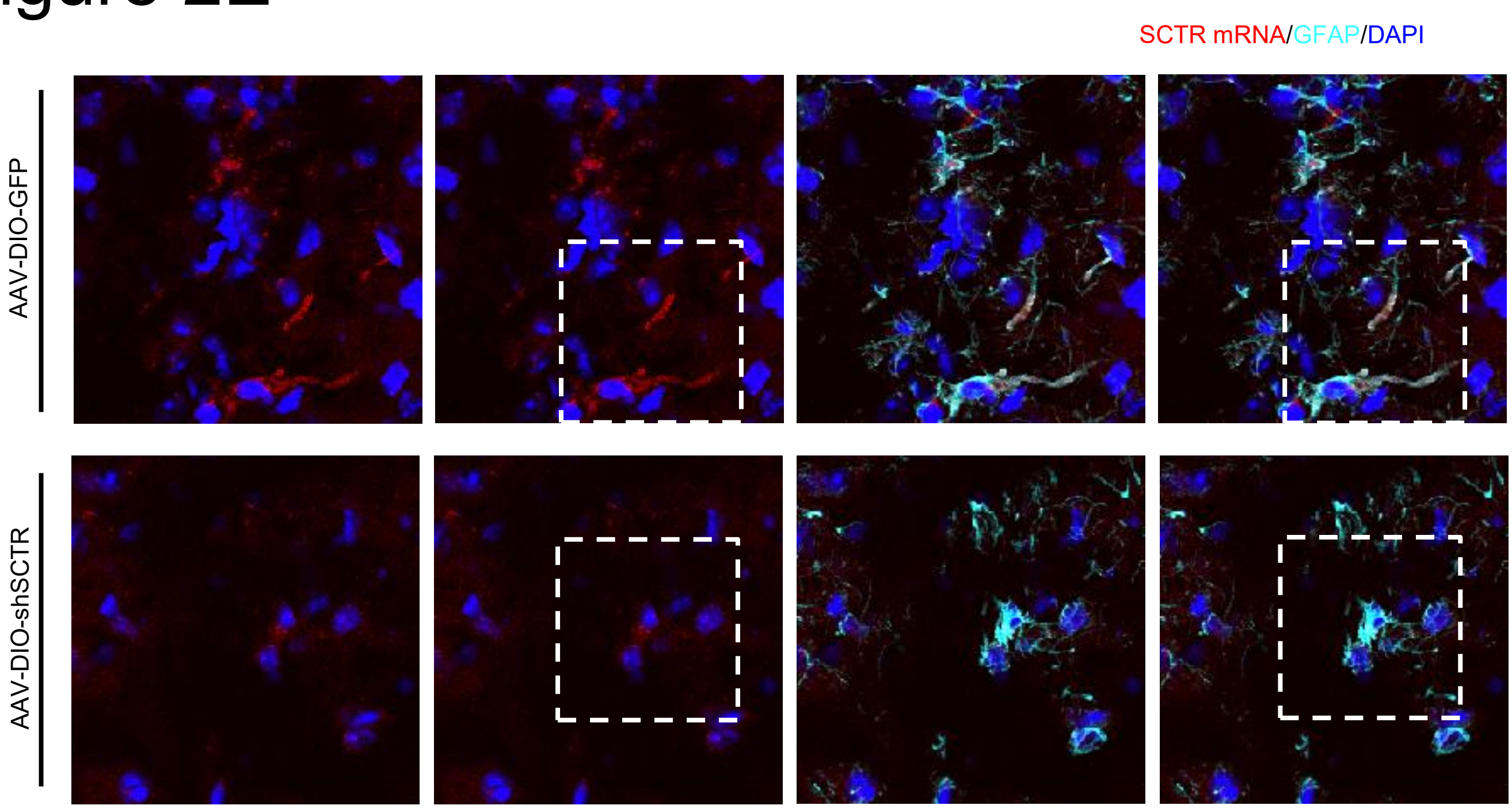

Figure 3A

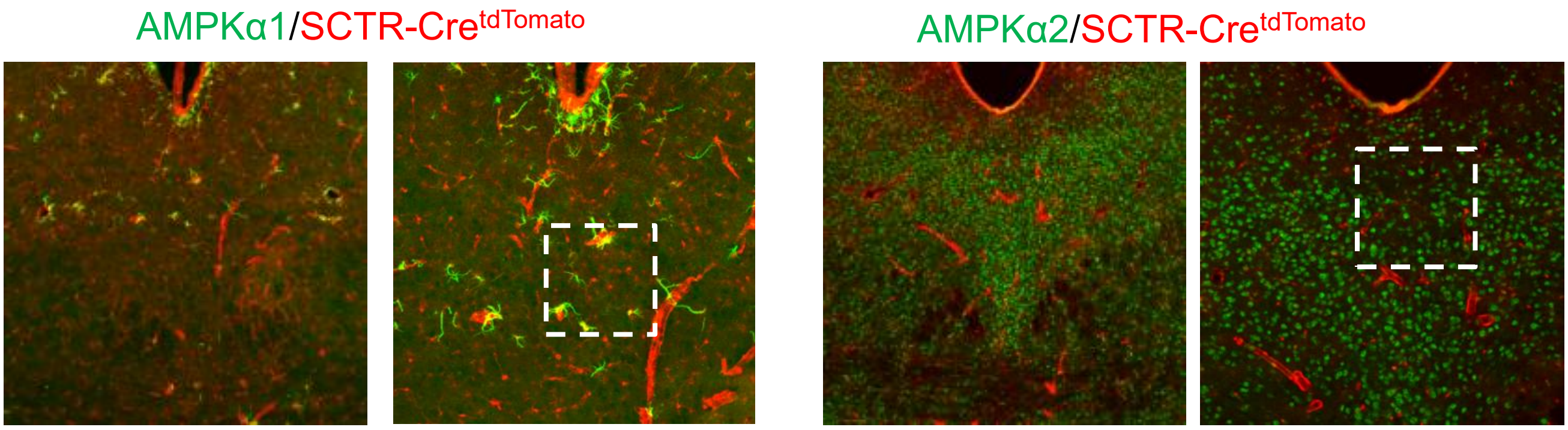

Figure 3B

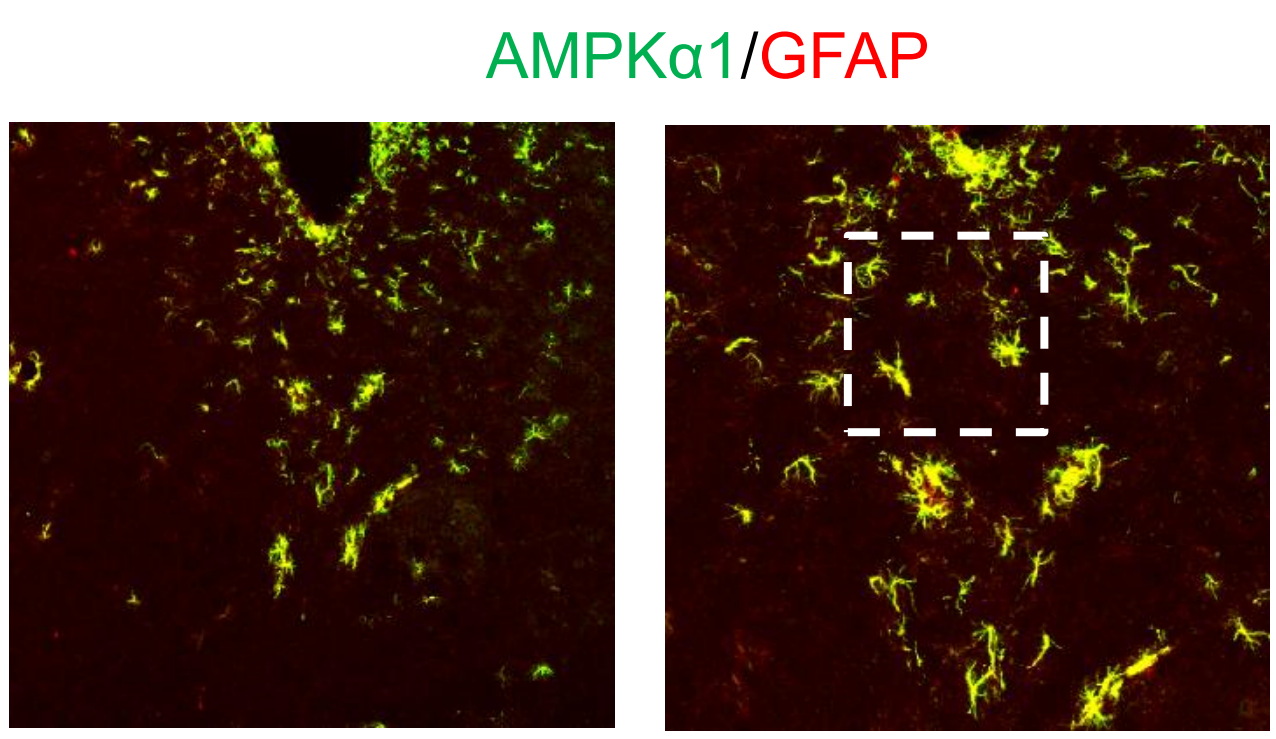

Figure 3D

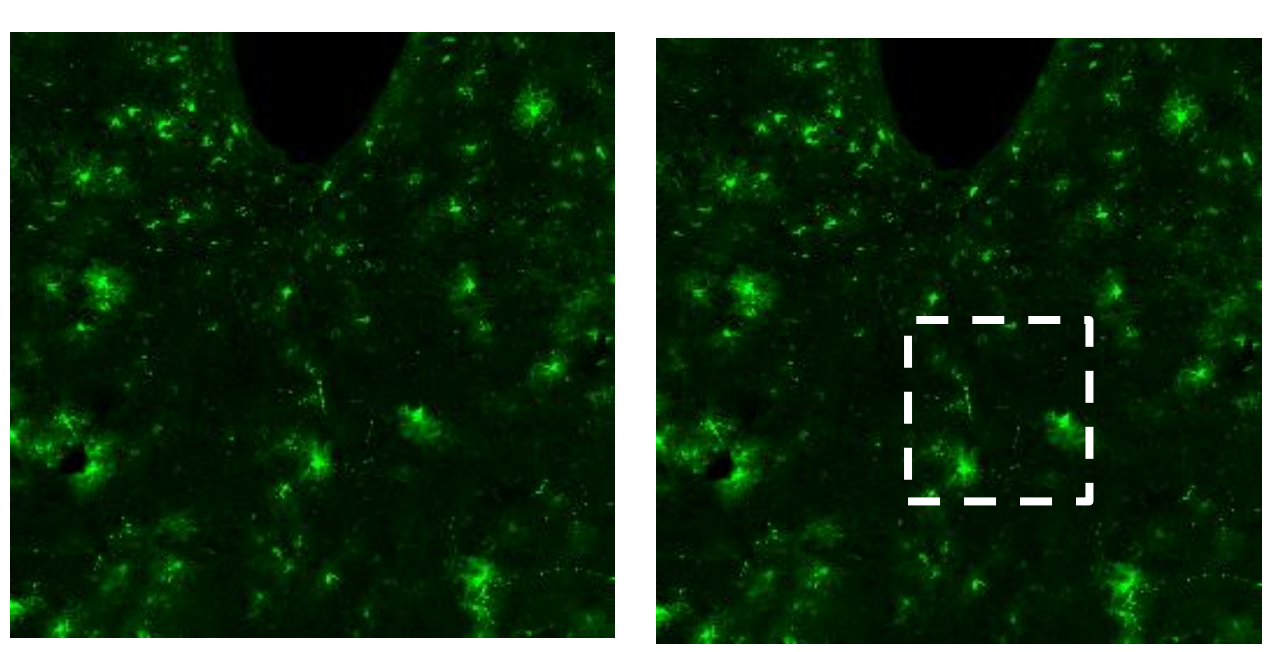

Figure 4D

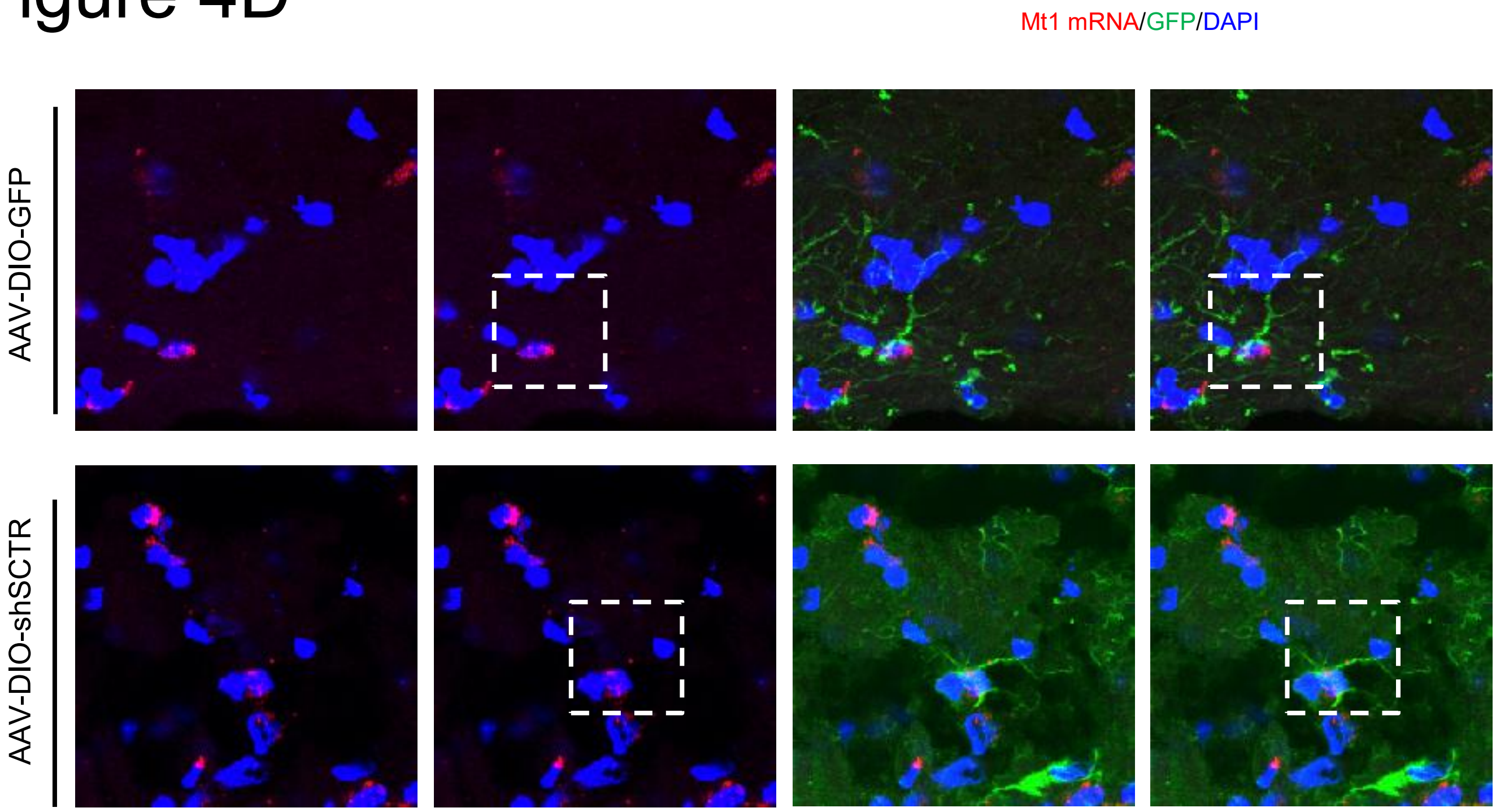

Figure 4J

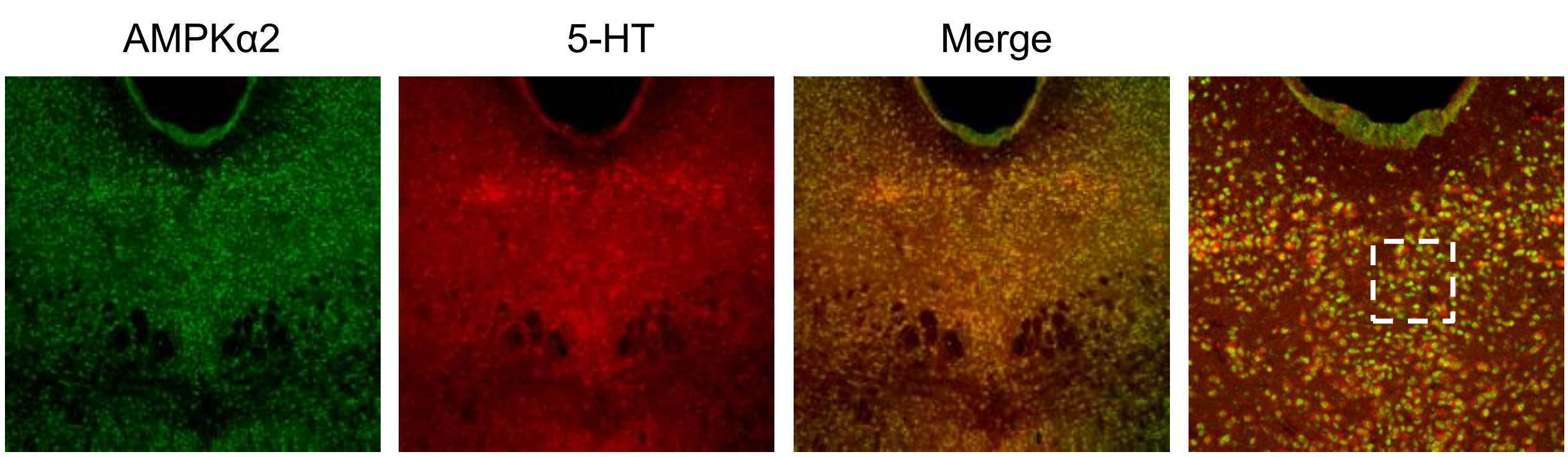

Figure 5B

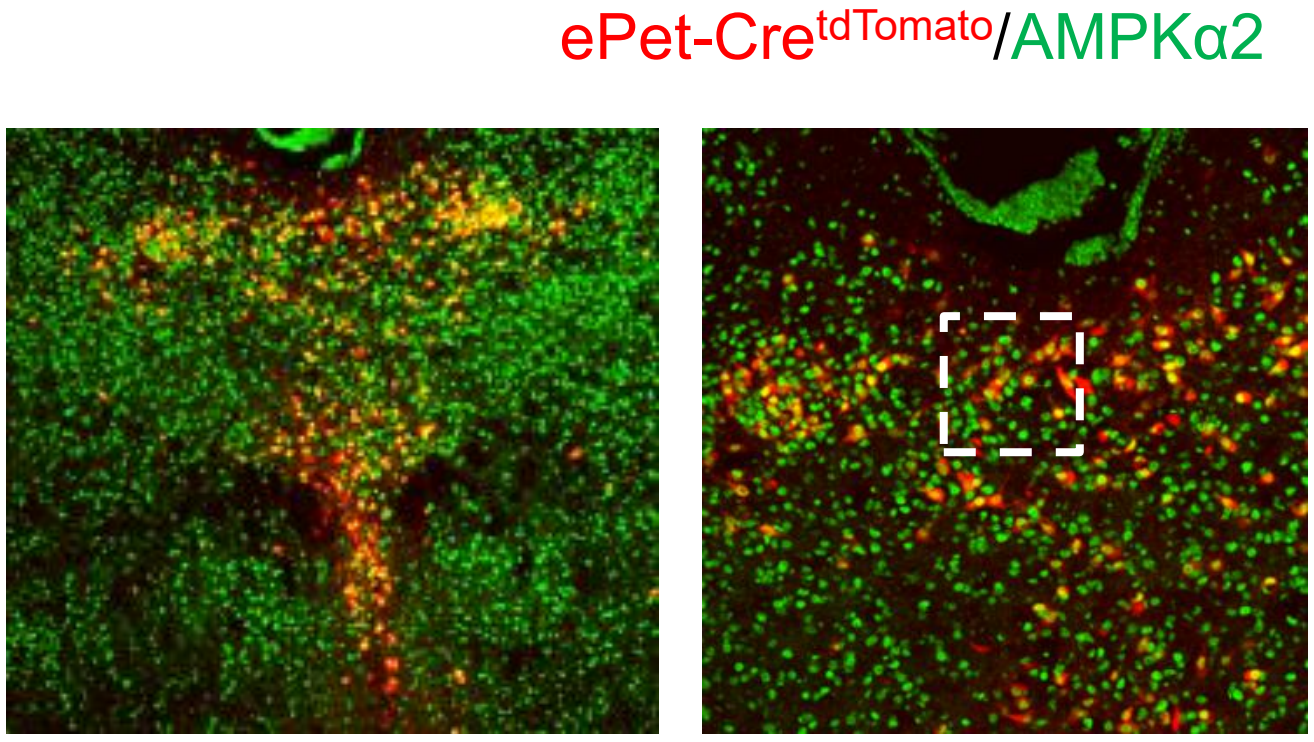

Figure 5L

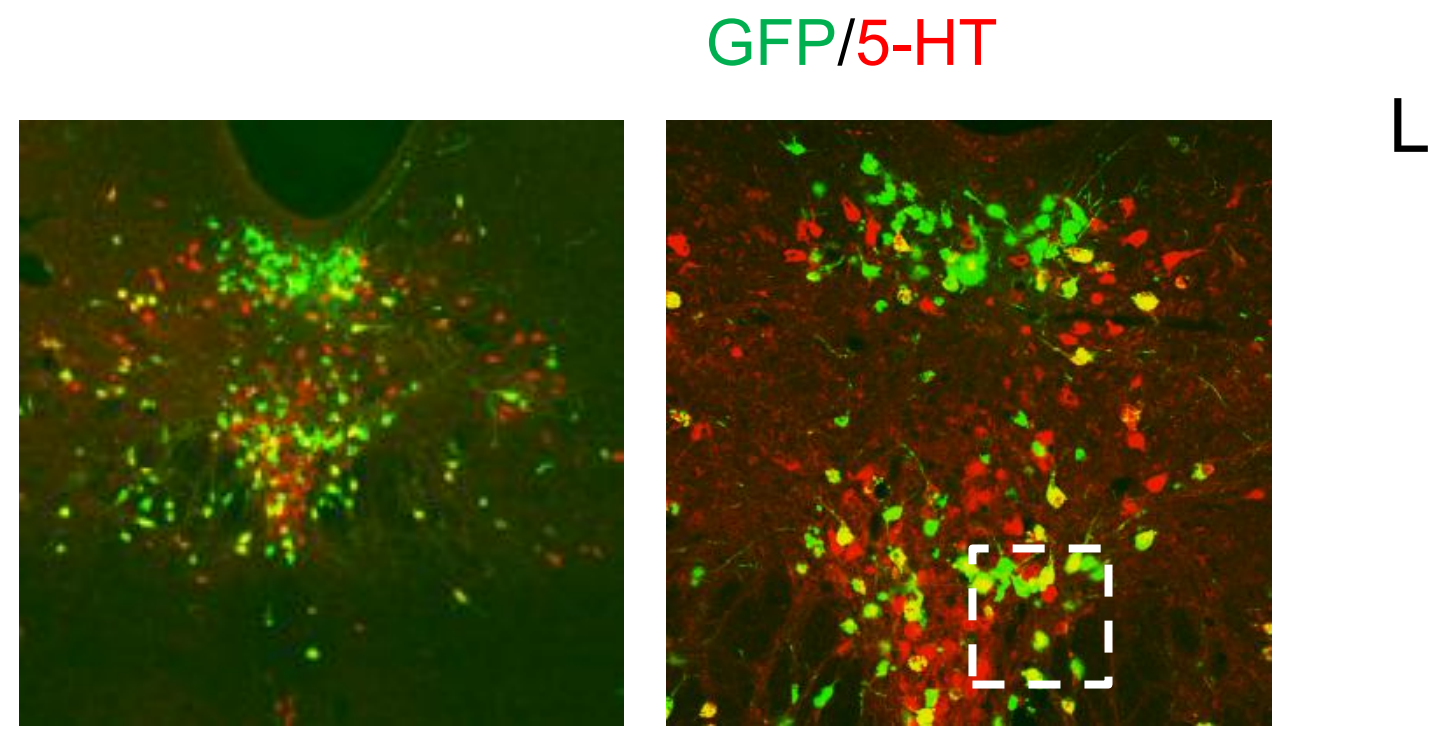

Figure 6B

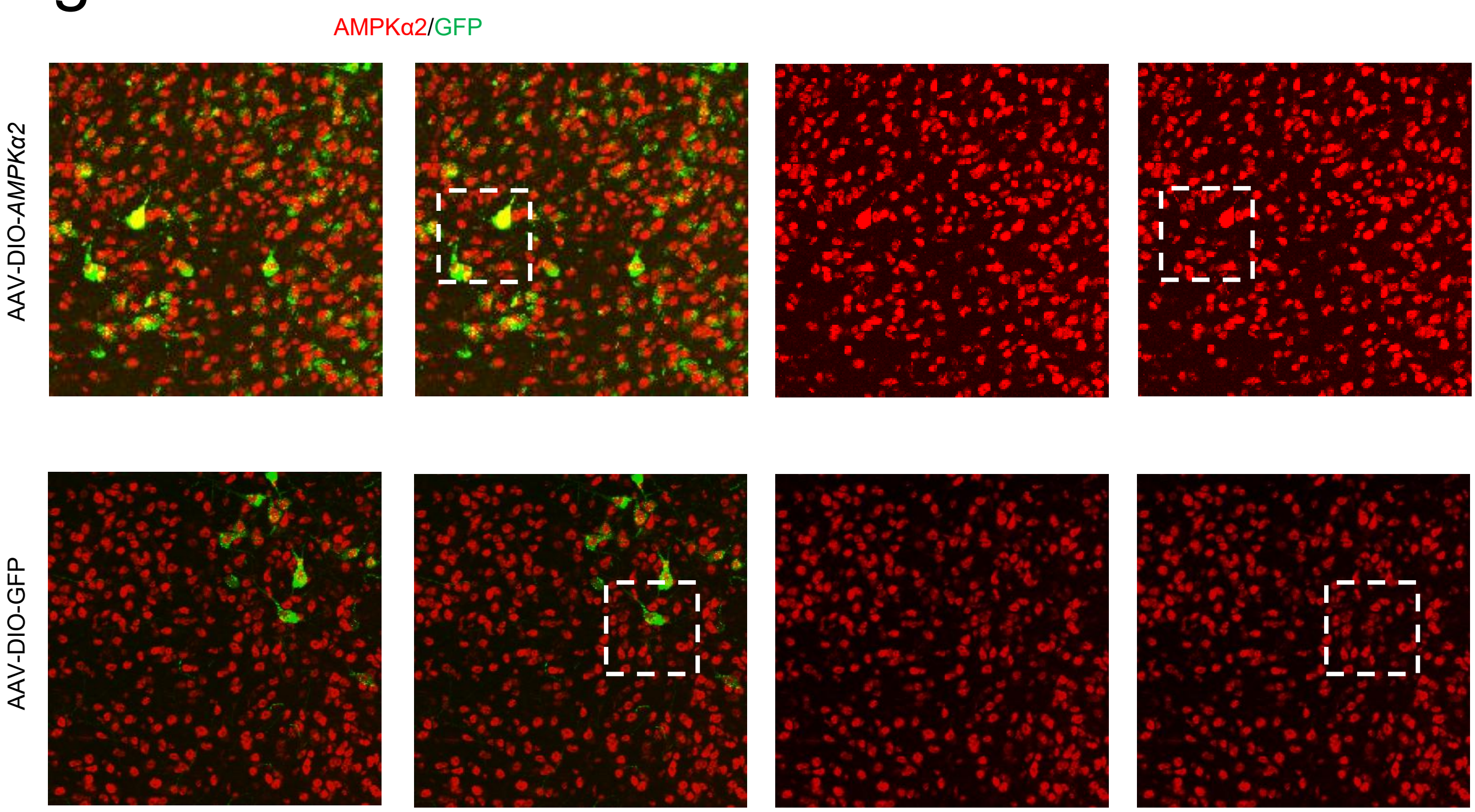

Figure 6I

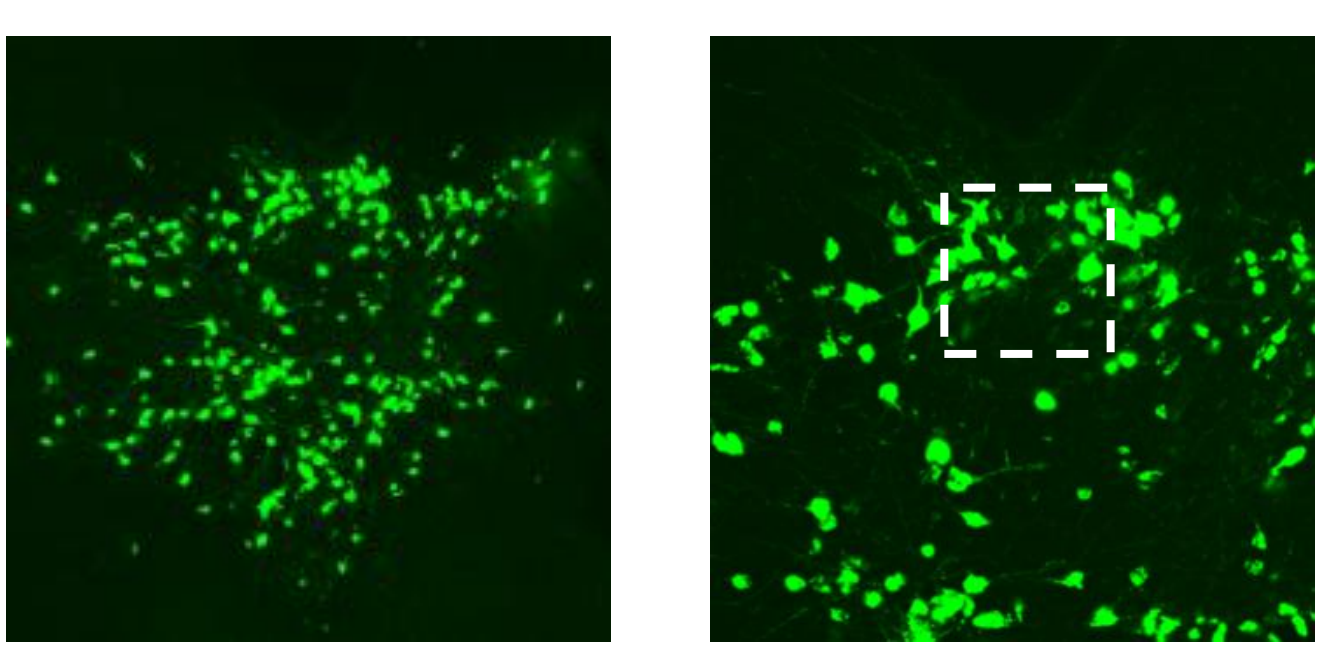

Figure 7F

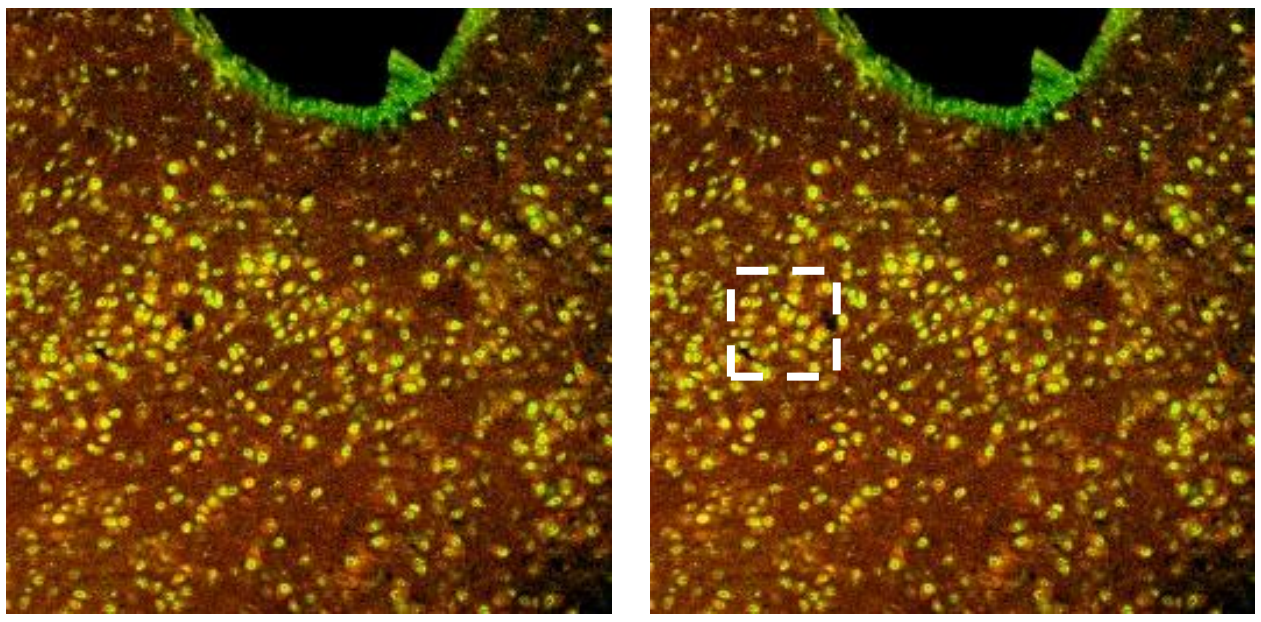

Figure 7H

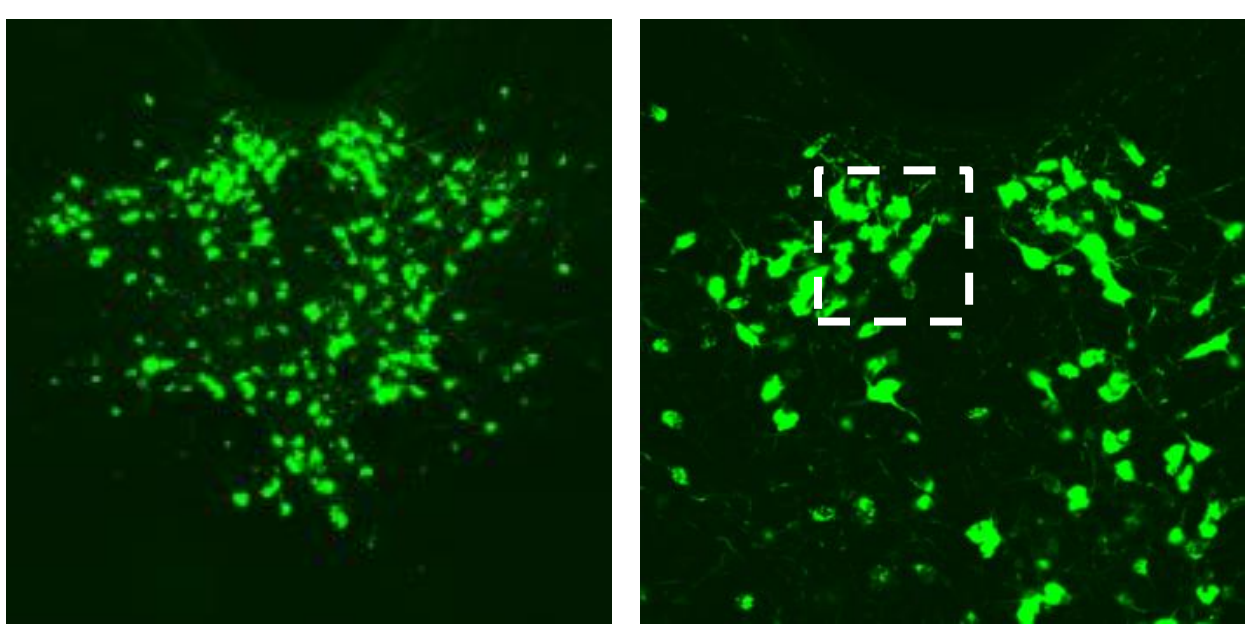

Figure 7O

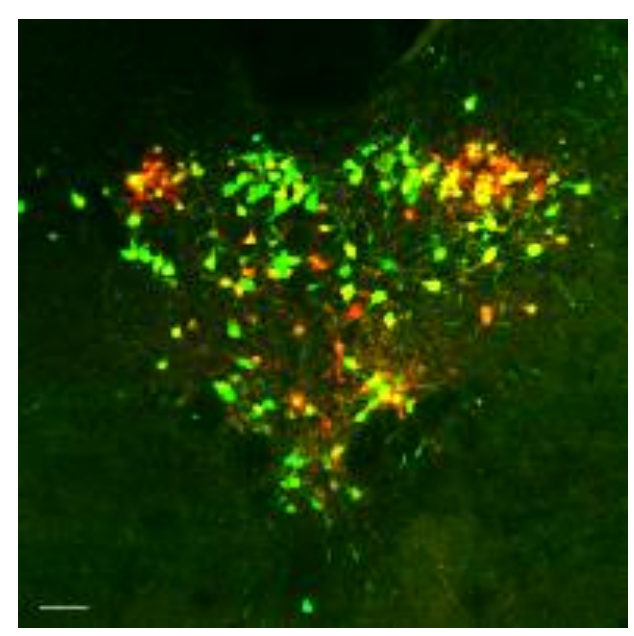

Figure 7Q

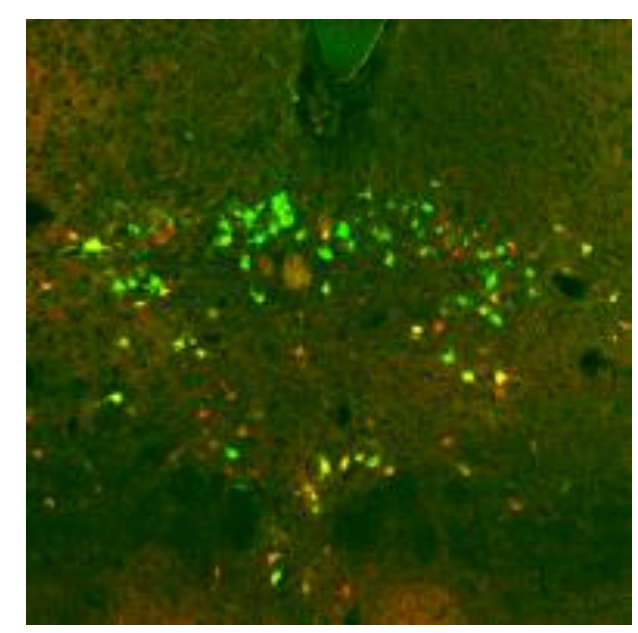

Figure 8D

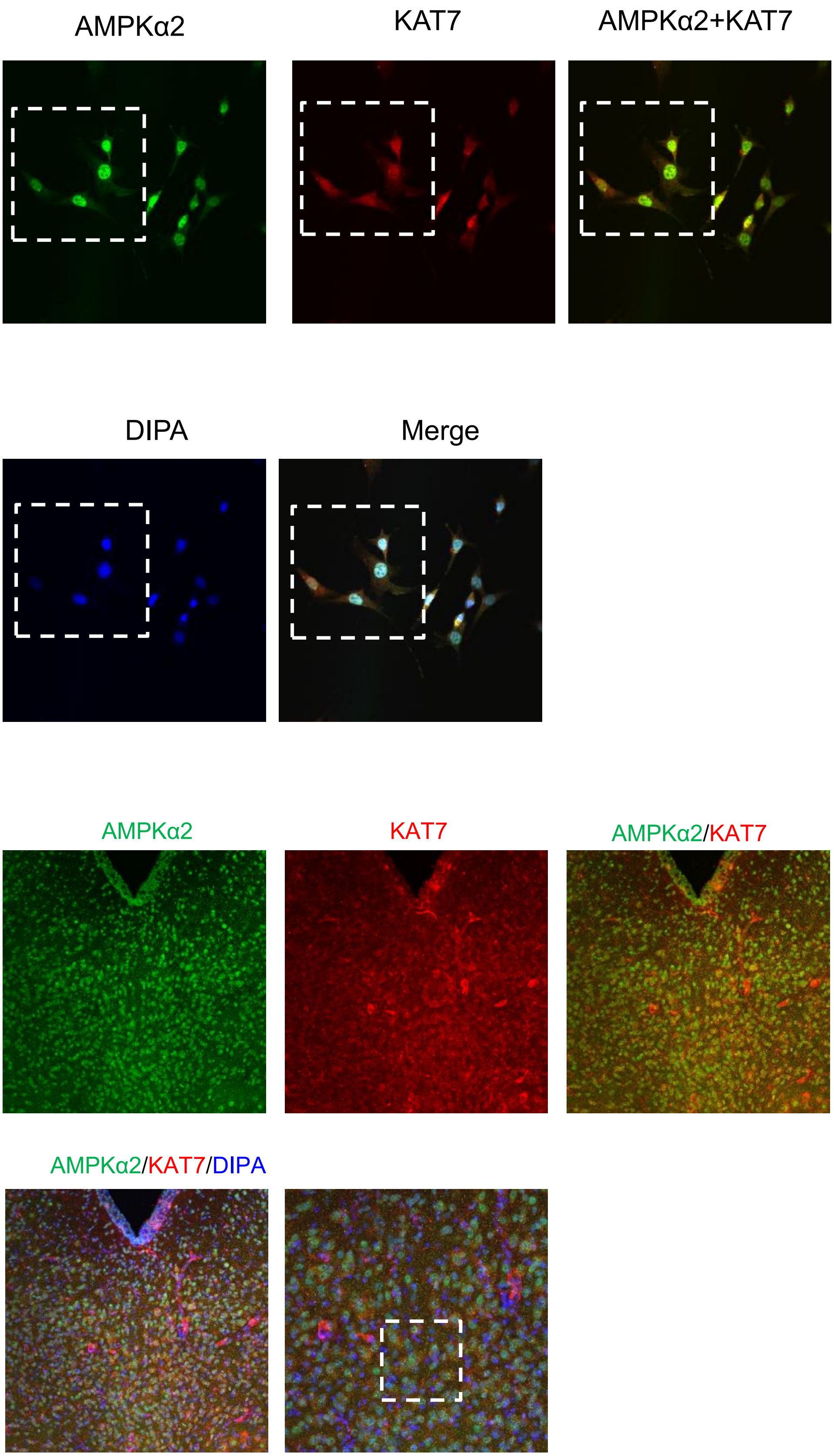
AMPKα2
KAT7
AMPKα2+KAT7
DIPA
Merge
AMPKα2
KAT7
AMPKα2/KAT7
AMPKα2/KAT7/DIPA

Figure S4B

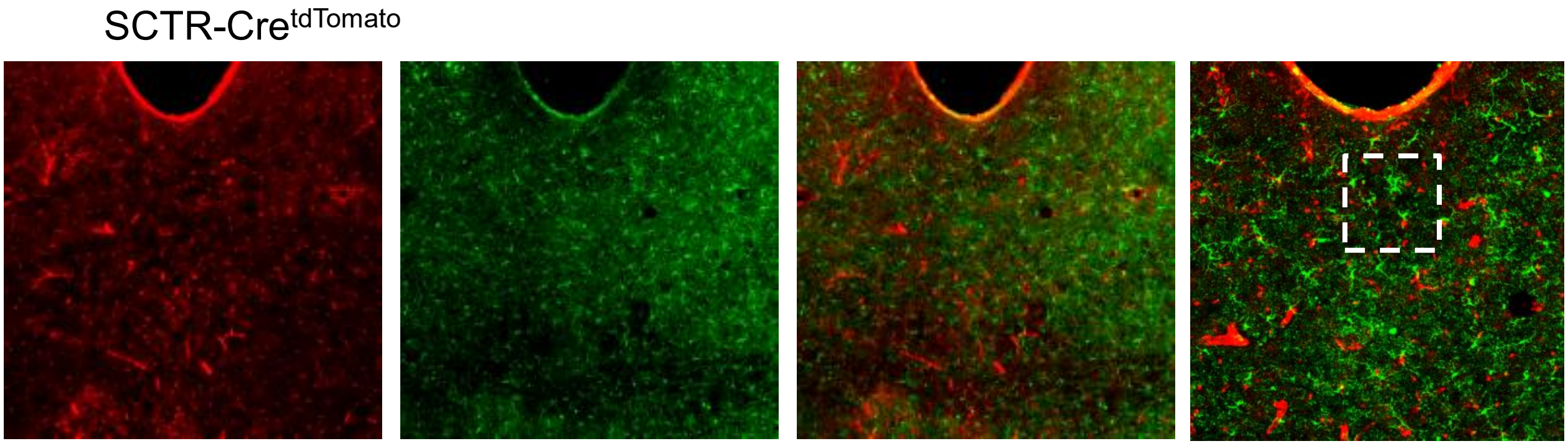

Figure S4C

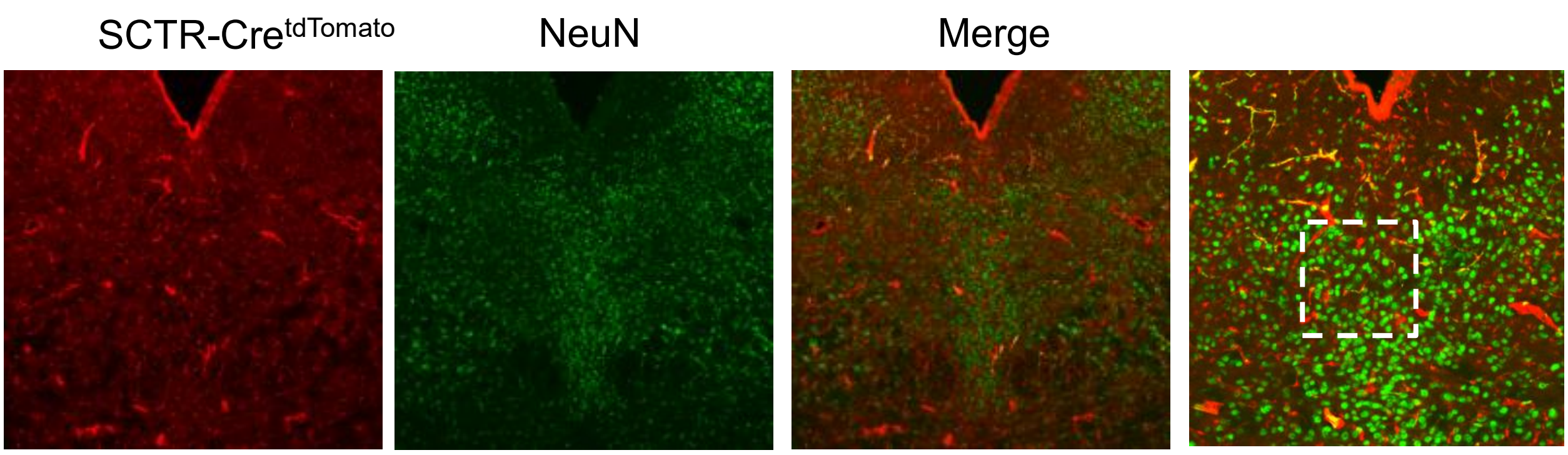

Figure S7A

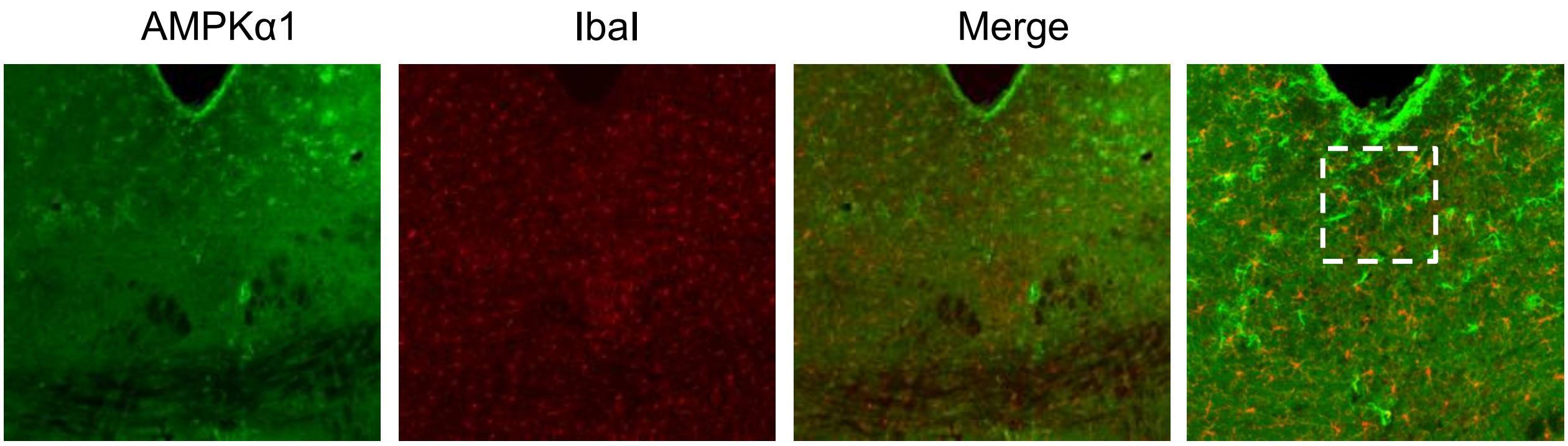

Figure S7B

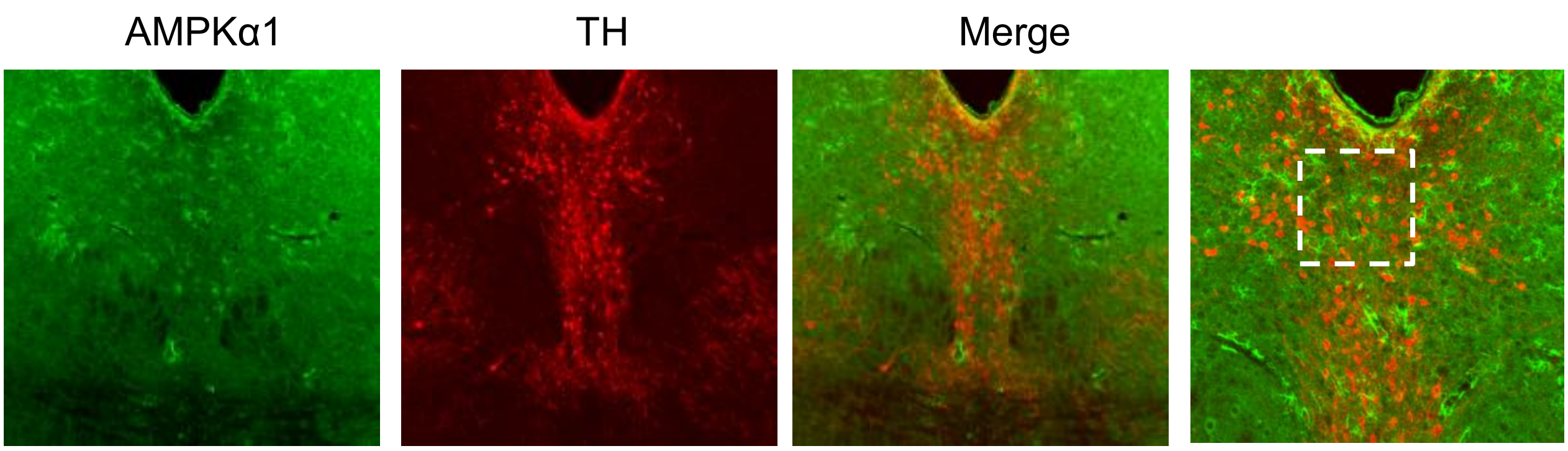

Figure S7C

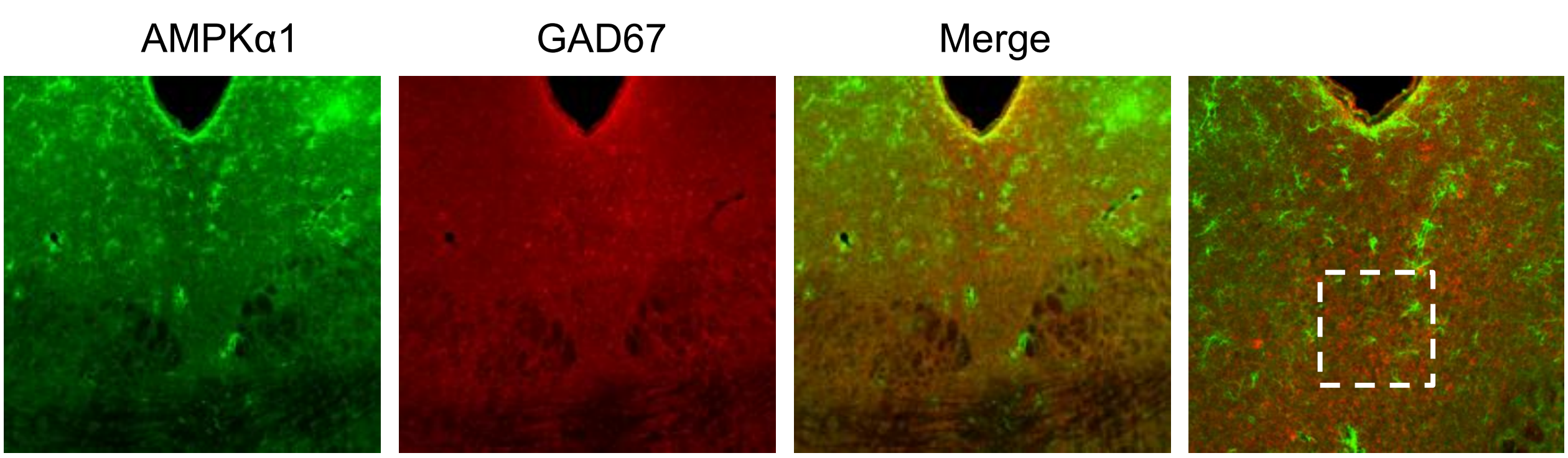

Figure S7D

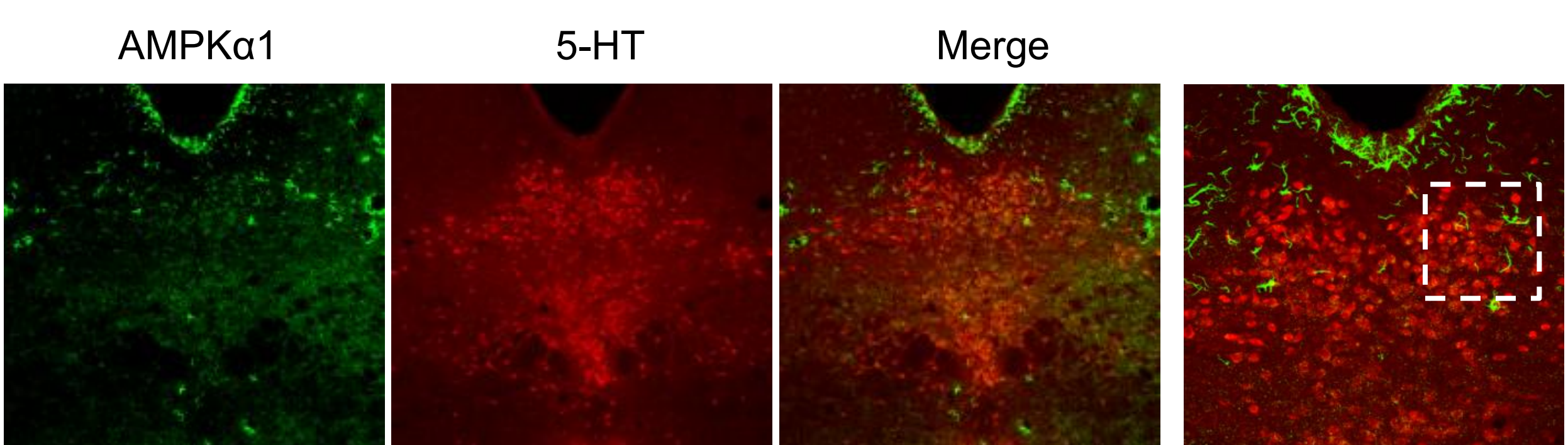

Figure S10D

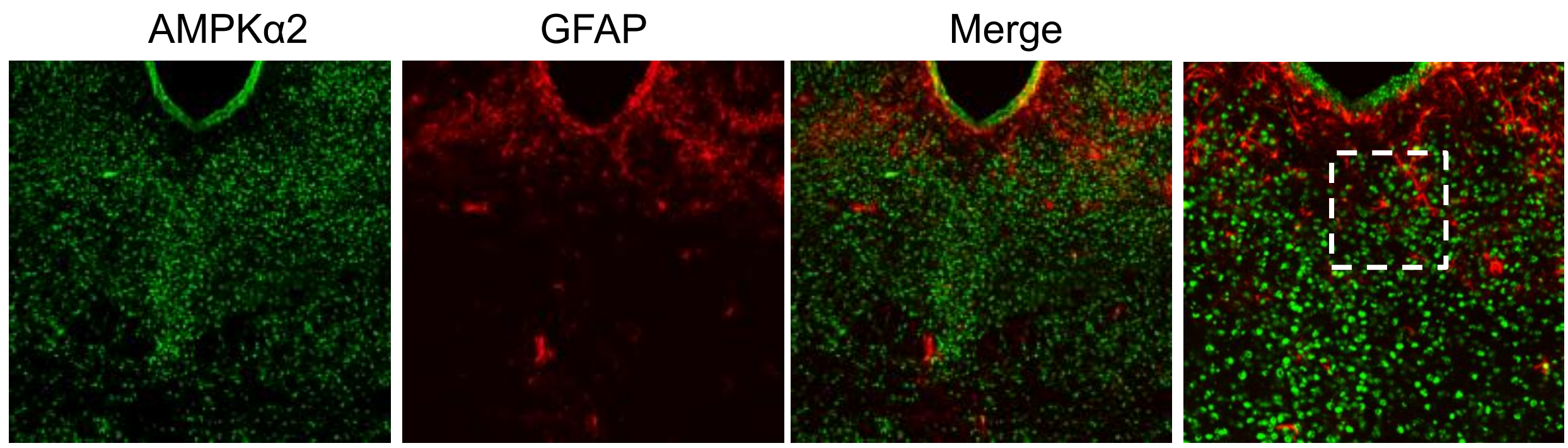

Figure S10E

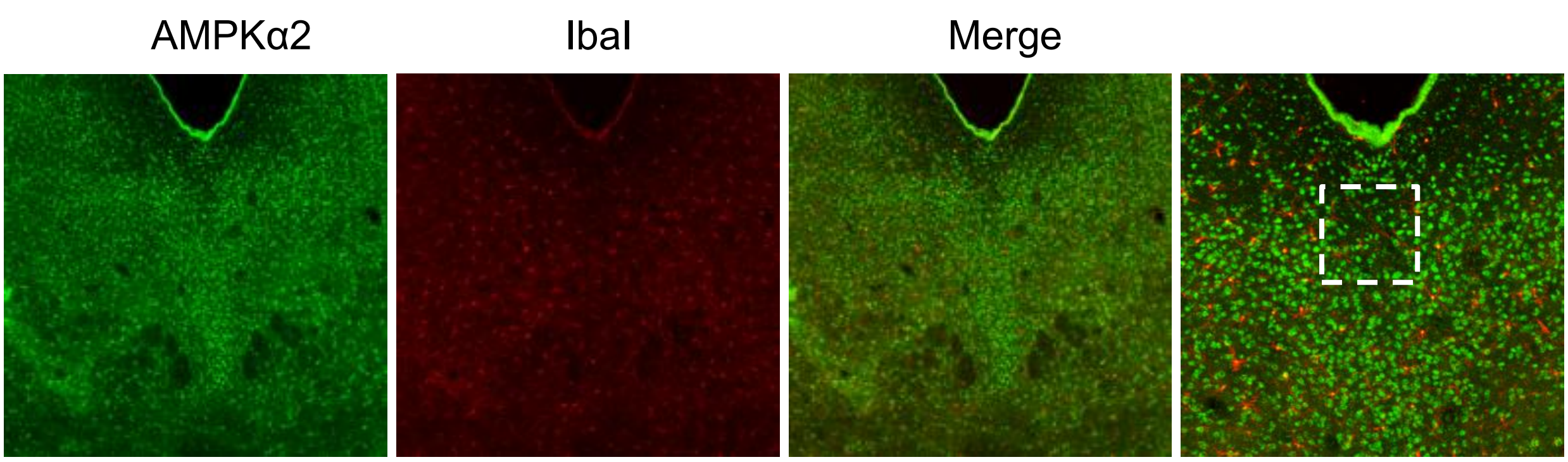

Figure S10F

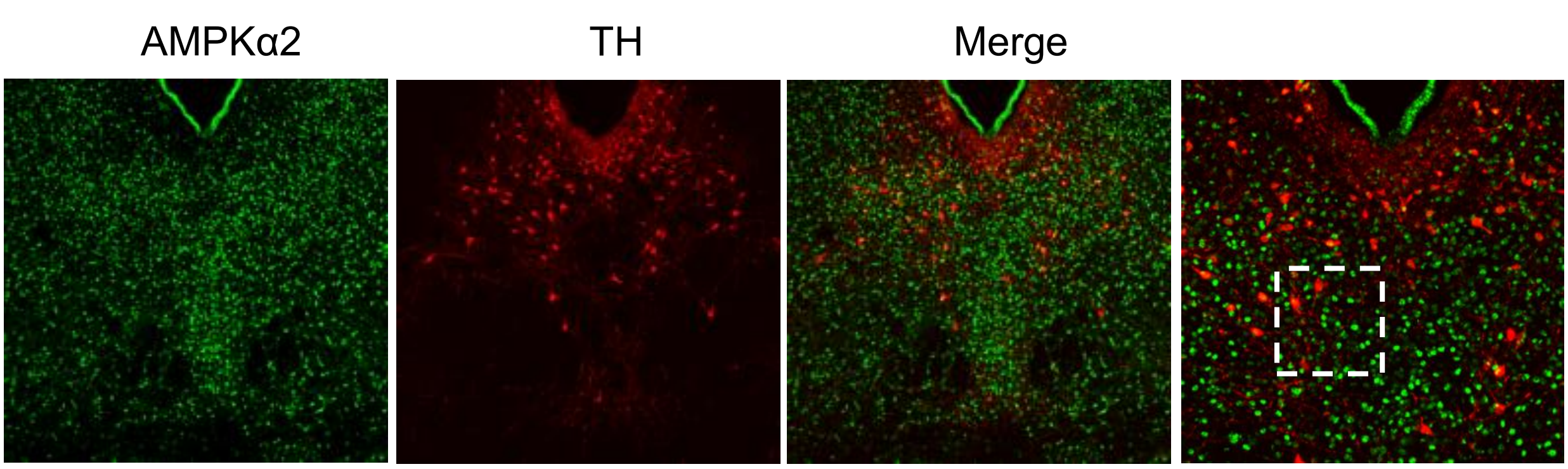

Figure S10G

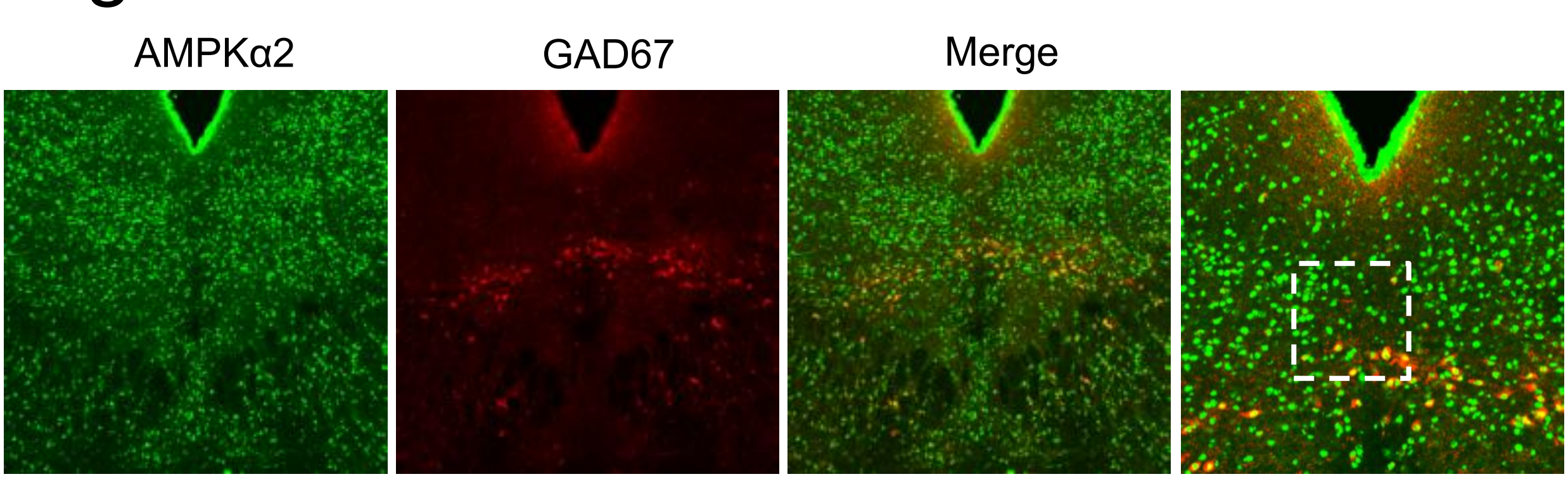

Figure S10H

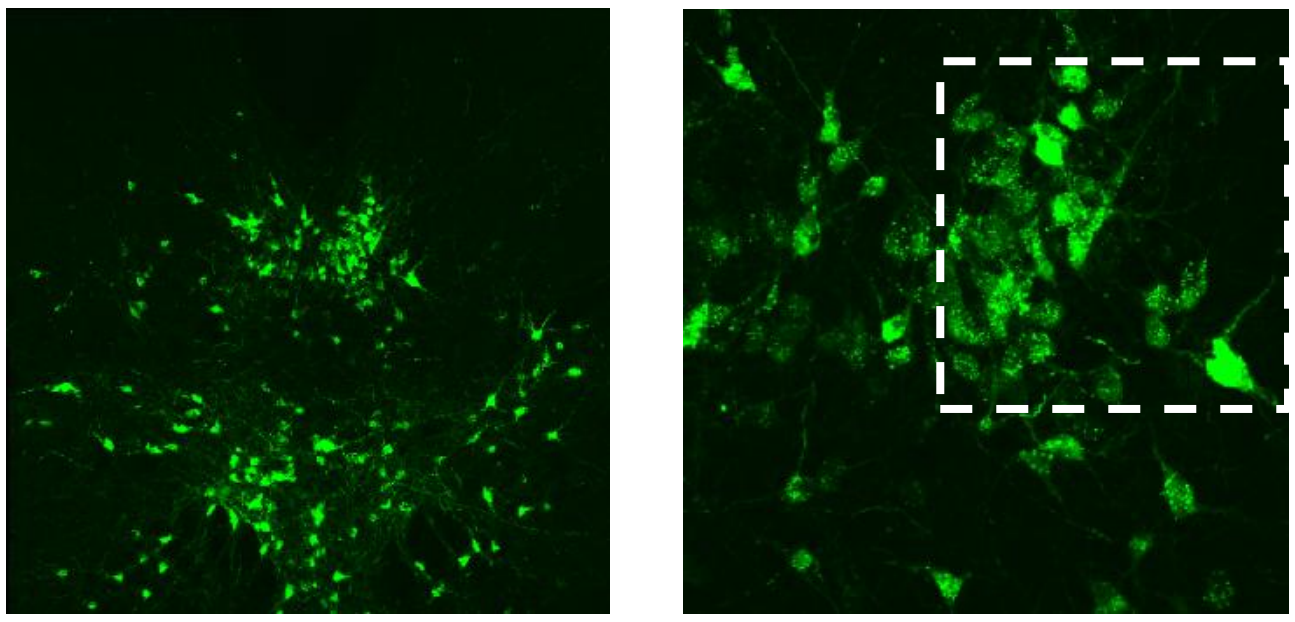

Figure S15B

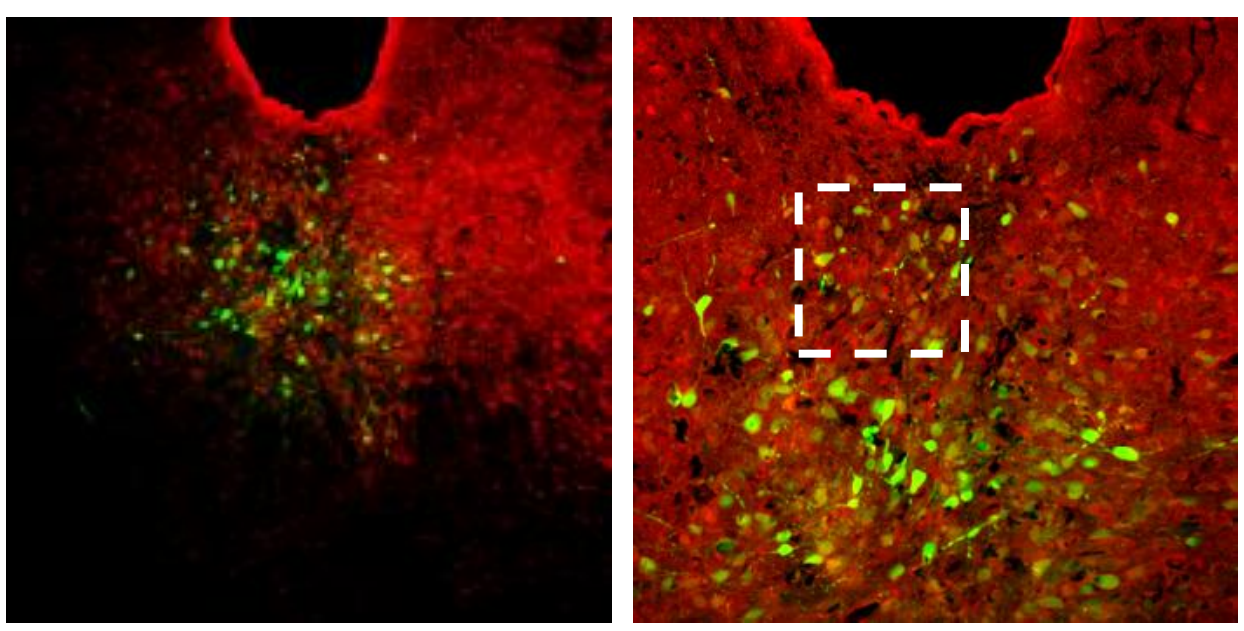

Figure S17B

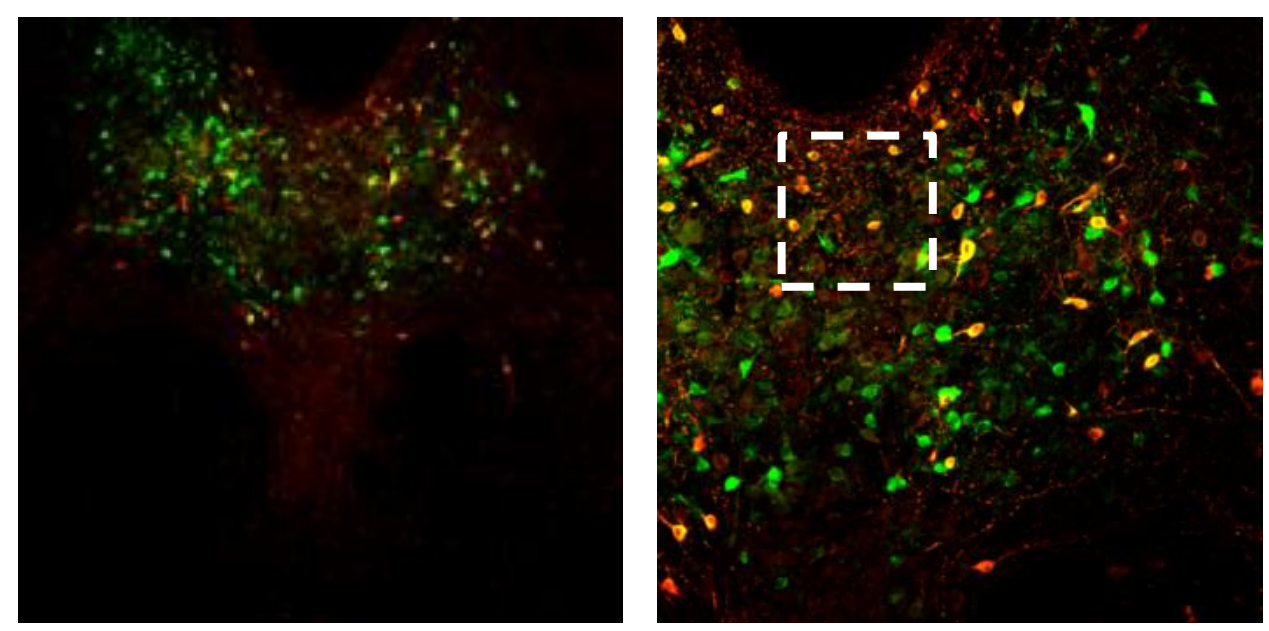

Figure S22B

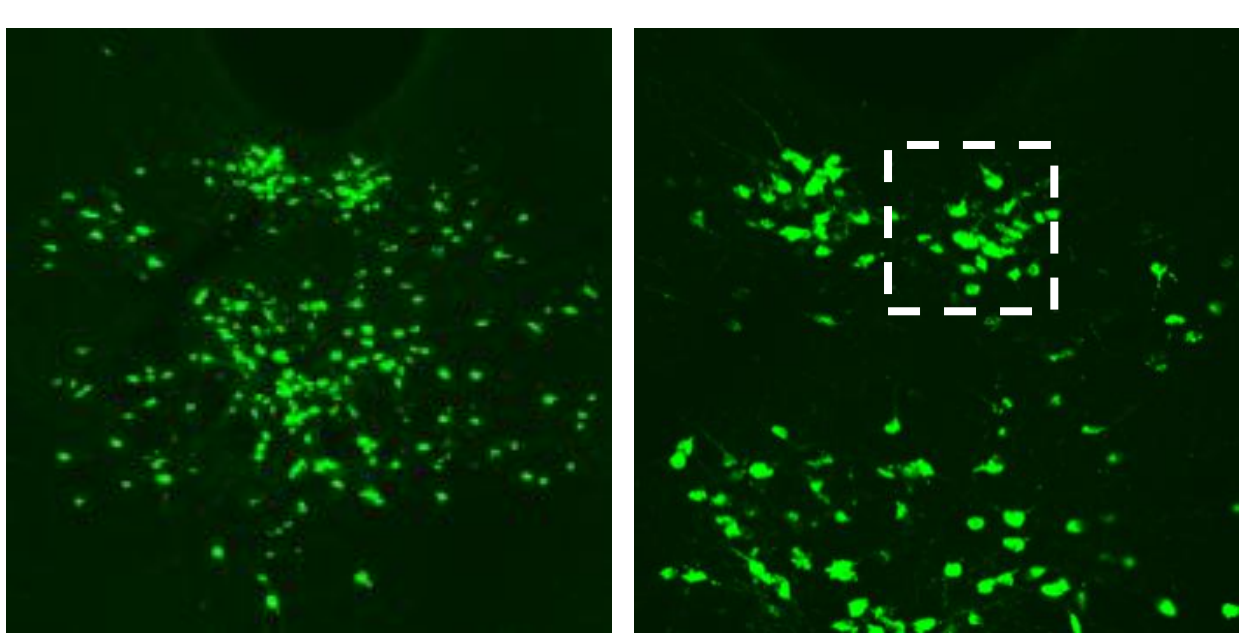

Figure S24B

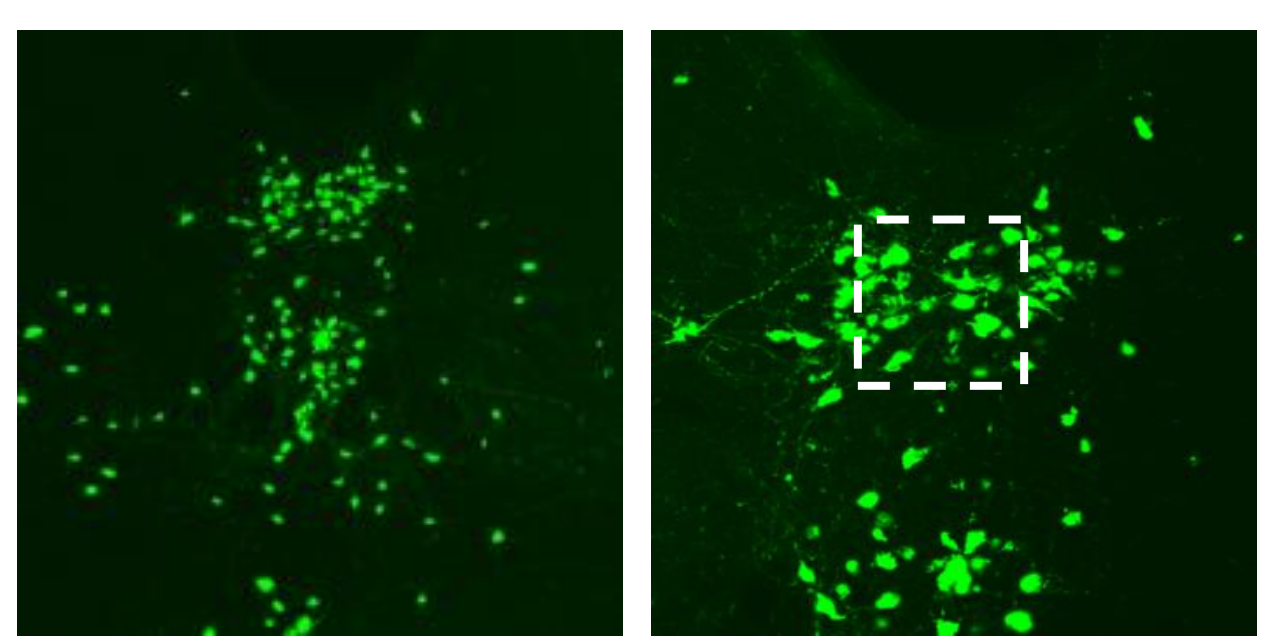

Supplement: Supplementary file 1 [file ADVS-9999-e77985-s006.pdf]
