# Supplementary material for: SCTR–Expressing Astrocyte–Serotonergic Neuron Signaling Drives Sexual Dimorphism in Depression Through KAT7/H4ac–Foxc2 Epigenetic Axis in an AMPK‐Dependent Manner
Source: Adv Sci (Weinh). 2026 Sep 27:e77985. Online ahead of print. doi: 10.1002/advs.77985 (PMC13616394; doi:10.1002/advs.77985)
Supplement: Supplementary file 2 [file ADVS-9999-e77985-s002.docx]

**Figure S1. CUS induced** **depression-like behaviors in female mice**.

(A) Statistical analysis of sucrose preference in the SPT. (B) Statistical analysis of latency to feed and food intake in the NSFT. (C) Statistical analysis of immobility time in the FST. (D) Representative activity tracking and total distance travelled during locomotor activity. female: control group, n=10 mice; CUS group, n=11 mice. (A: two-tailed unpaired t test with Welch’s correction; B left, D: Mann-Whitney U test; B right: two-way ANOVA followed by Holm-sidak’s post-hoc test; C: two-tailed unpaired Student’s t test.) Data are presented as the means ± S.E.M. **P* < 0.05, ***P* < 0.01.

**Figure S2. SCTR knockout female mice exhibited little effect on depression-like behaviors**.

(A)Schematic illustration of the strategy for generating SCTR-knockout mice. (B) Statistical analysis of sucrose preference in the SPT. (C) Statistical analysis of the latency to feed and food intake in the NSFT. (D) Statistical analysis of immobility time in the FST. (E) Representative activity tracking and total distance travelled during locomotor activity. female: n = 14 mice for the WT group, n = 14 mice for the *SCTR*^-/-^ group. (B, C left: Mann-Whitney U test; D, E: two-tailed unpaired Student’s t test; C right: two-way ANOVA followed by Holm-sidak’s post-hoc test.) Data are presented as the means ± S.E.M. **P* < 0.05.

**Figure S3. Anxiety-like behaviors in SCTR knockout mice**.

(A) Statistical analysis of open arm time, entries, and total arm entries during EPMT in male mice. (B) Statistical analysis of latency to the light side and time spent on the light side during the LDT in male mice. (C) Representative heat map of exploratory activity, time in the center, and total distance travelled during the OFT for male mice. male: n = 13 mice for the WT group, n = 14 mice for the *SCTR*^-/-^ group. (A left, B left, C left, C right: Mann-Whitney U test; A middle, A right, B right: two-tailed unpaired Student’s t test.) (D) Statistical analysis of open arm time, entries, and total arm entries during EPMT in female mice. (E) Statistical analysis of latency to the light side and time spent on the light side during the LDT in female mice. (F) Representative heat map of exploratory activity, time in the center, and total distance travelled during the OFT for female mice. female: n=14 mice for the WT group, n = 14 mice for the *SCTR*^-/-^ group. (D left, E left: Mann-Whitney U test; D middle, D right, E right, F left, F right: two-tailed unpaired Student’s t test.) Data are presented as the means ± S.E.M. **P* < 0.05.

**Figure S4. Colocalization of SCTR with microglia and neurons in the DRN**.

(A) Schematic illustration of the strategy for generating *SCTR*-Cre mice. (B and C) Representative images showing the colocalization of tdTomato (red) and Iba1 (green) (B) or NeuN (green) (C) in the DRN. Scale bars = 100μm (left), 50μm (right). (n = 6 replicates from three mice).

**Figure S5. Depression-like behaviors in female mice with astrocyte-specific deletion of SCTR in the DRN**.

(A) Western blotting images and quantification of SCTR in the DRN between male and female wild-type mice. n = 6 per group, two-tailed unpaired Student’s t test. (B) Statistical analysis of sucrose preference in the SPT. (C) Statistical analysis of latency to feed and food intake in the NSFT. (D) Statistical analysis of immobility time in the FST. (E) Representative activity tracking and total distance travelled during locomotor activity. Females: n = 10 mice in the AAV-DIO-GFP group, n = 10 mice for the AAV-DIO-sh*SCTR*-GFP group. (C left, E: two-tailed unpaired Student’s t test; B, D: Mann-Whitney U test; C right: two-way ANOVA followed by Holm-sidak’s post-hoc test.) Data are presented as the means ± S.E.M. ***P* < 0.01.

**Figure S6. Anxiety-like behaviors in mice with astrocyte-specific deletion of SCTR in the DRN**.

(A) Statistical analysis of open arm time, entries, and total arm entries during EPMT in male mice. (B) Statistical analysis of latency to the light side and time spent on the light side during the LDT in male mice. (C) Representative heat map of exploratory activity, time in the center, and total distance travelled during the OFT for male mice.  n = 9 mice for the AAV-DIO-GFP group, n=8 mice for the AAV-DIO-sh*SCTR*-GFP group. (A left, A middle, A right, B left, B right, C right: two-tailed unpaired Student’s t test; C left, two-tailed unpaired t test with Welch’s correction.) (D) Statistical analysis of open arm time, entries, and total arm entries during EPMT in female mice. (E) Statistical analysis of latency to the light side and time spent on the light side during the LDT in female mice. (F) Representative heat map of exploratory activity, time in the center, and total distance travelled during OFT for female mice. Females: n = 10 mice for the AAV-DIO-GFP group, n = 10 mice for the AAV-DIO-sh*SCTR*-GFP group. (D left, D middle, D right, E right, F left: two-tailed unpaired Student’s t test; E left, F right: Mann-Whitney U test.) Data are presented as the means ± S.E.M.

**Figure S7. Distribution of AMPKα1 in the dorsal raphe nucleus**.

(A to D), Representative images showing colocalization of AMPKα1 (green) and Iba1 (red) (A), TH (red) (B), GAD67 (red) (C), or 5-HT (red) (D), in the DRN. Scale bars = 100μm (left), 50μm (right). (n = 6 replicates from three mice).

**Figure S8. Depression-like behaviors in female mice with cell-type-specific knockdown of AMPKα1 in SCTR-expressing cells in the DRN**.

(A) Western blotting images and quantification of AMPKα1 in the DRN between male and female wild-type mice. n = 6 per group, two-tailed unpaired Student’s t test. (B) Statistical analysis of sucrose preference in the SPT. (C) Statistical analysis of latency to feed and food intake in the NSFT. (D) Statistical analysis of immobility time in the FST. (E) Representative activity tracking and total distance travelled during locomotor activity. female: n = 10 mice for the AAV-DIO-GFP group, n = 10 mice for the AAV-DIO-sh*AMPKα1*-GFP group. (B: two-tailed unpaired Student’s t test; C left, D, E: Mann-Whitney U test; C right: two-way ANOVA followed by Holm-sidak’s post-hoc test.) Data are presented as the means ± S.E.M. ***P* < 0.01.

**Figure S9. Anxiety-like behaviors in mice with cell-type-specific knockdown of AMPKα1 in SCTR-expressing cells in the DRN**.

(A) Statistical analysis of open arm time, entries, and total arm entries during EPMT in male mice. (B) Statistical analysis of latency to the light side and time spent on the light side during the LDT in male mice. (C) Representative heat map of exploratory activity, time in the center, and total distance travelled during the OFT for male mice. male: n = 10 mice for the AAV-DIO-GFP group, n = 10 mice for the AAV-DIO-sh*AMPKα1*-GFP group. (A left: Mann-Whitney U test; A middle, A right, B left, B right, C right: two-tailed unpaired Student’s t test; C left: two-tailed unpaired t test with Welch’s correction.) (D)Statistical analysis of open arm time, entries, and total arm entries during EPMT in female mice. (E) Statistical analysis of latency to the light side and time spent on the light side during the LDT in female mice. (F) Representative heat map of exploratory activity, time in the center, and total distance travelled during the OFT for female mice. female: n = 10 mice for the AAV-DIO-GFP group, n = 10 mice for the AAV-DIO-sh*AMPKα1*-GFP group. (D left, D middle, D right, F right: two-tailed unpaired Student’s t test; E left, E right, F left: Mann-Whitney U test.) Data are presented as the means ± S.E.M. *P < 0.05, **P < 0.01.

**Figure S10. Distribution of AMPKα2 in the dorsal raphe nucleus**.

(A) Schematic illustration of the generation of an SCTR-knockdown C8-D1A cell line via lentiviral transduction of SCTR-targeting shRNA. (B) Statistical analysis of Mt1 levels in the culture supernatant of SCTR-knockdown cells. n = 4 per group, two-tailed unpaired Student’s t test. (C) qPCR analysis confirmed the efficient knockdown of SCTR in the established C8-D1A cell line. n = 3 per group, two-tailed unpaired t test with Welch’s correction. (D to G), Representative images showing colocalization of AMPKα2 (green) and GFAP (red) (D), IbaI (red) (E), TH (red) (F), or GAD67 (red) (G) in the DRN. Scale bars =100μm (left), 50μm (right). (n = 6 replicates from three mice). (H) left panel: Representative images of GFP expression in the DRN. Scale bars = 50μm. Right panel: qPCR analysis of megalin mRNA levels in the DRN of *ePet*-cre mice, with virus injection of AAV-DIO-GFP or AAV-DIO-sh*Megalin*-GFP, followed by Mt1 treatment. n = 5 per group, one-way ANOVA followed by Sidak’s multiple comparison test. (I) qPCR analysis of megalin mRNA levels in HT22 cells treated with Mt1 peptides or megalin siRNA. n = 3 per group, one-way ANOVA followed by Sidak’s multiple comparison test. Data are presented as the means ± S.E.M. *P < 0.05, **P < 0.01.

**Figure S11. Depression-like behaviors in female mice with Serotonergic neuron-specific knockout of AMPKα2**.

(A) Western blotting images and quantification of AMPKα2 in the DRN between male and female wild-type mice. n = 6 per group, two-tailed unpaired Student’s t test. (B) Statistical analysis of sucrose preference in the SPT. (C) Statistical analysis of latency to feed and food intake in the NSFT. (D) Statistical analysis of immobility time in the FST. (E) Representative activity tracking and total distance travelled during locomotor activity. female: n = 10 mice for the *AMPKα2*^flox/flox^ group, n = 9 mice for the *AMPKα2*^flox/flox; ePet-Cre^ group. (B, C left, D, E: Mann-Whitney U test; C right: two-way ANOVA followed by Holm-sidak’s post-hoc test.) Data are presented as the means ± S.E.M. **P* < 0.05.

**Figure S12. Anxiety-like behaviors in mice with Serotonergic neuron-specific knockout AMPKα2**.

(A) Statistical analysis of open arm time, entries, and total arm entries during EPMT. (B) Statistical analysis of latency to the light side and time on the light side during the LDT. (C) Representative heat map of exploratory activity, time in the center, and total distance travelled during the OFT. male: n = 10 mice for the *AMPKα2*^flox/flox^ group, n = 10 mice for the *AMPKα2*^flox/flox; ePet-Cre^ group. (A left, A middle, A right, B left, B right: two-tailed unpaired Student’s t test; C left, C right: Mann-Whitney U test.) (D) Statistical analysis of open arm time, entries, and total arm entries during EPMT. (E) Statistical analysis of latency to the light side and time on the light side during the LDT. (F) Representative heat map of exploratory activity, time in the center, and total distance travelled during the OFT. female: n = 10 mice for the AMPKα2^flox/flox^ group, n = 9 mice for the *AMPKα2*^flox/flox; ePet-Cre^ group. (D left, D middle, E left, E right, F left, F right: two-tailed unpaired Student’s t test; D right: Mann-Whitney U test.) Data are presented as the means ± S.E.M. **P* < 0.05.

**Figure S13. Anxiety-like behaviors in male mice with knockout of AMPKα2 in serotonergic neurons of the DRN**.

(A) Statistical analysis of open arm time, entries, and total arm entries during EPMT. (B) Statistical analysis of latency to the light side and time on the light side during the LDT. (C) Representative heat map of exploratory activity, time in the center, and total distance travelled during the OFT. male: n = 9 mice, AAV-TPH2-GFP group; n = 8 mice for the AAV-TPH2-Cre-GFP group. (A middle, A right, B left, C left, C right: two-tailed unpaired Student’s t test; A left: Mann-Whitney U test; B right: two-tailed unpaired t test with Welch’s correction.) Data are presented as the means ± S.E.M. *P < 0.05.

**Figure S14. Depression-like and anxiety-like behaviors in female mice with knockout of AMPKα2 in serotonergic neurons of the DRN**.

(A) Statistical analysis of sucrose preference in the SPT. (B) Statistical analysis of latency to feed and food intake in the NSFT. (C) Statistical analysis of immobility time in the FST. (D) Representative activity tracking and total distance travelled during locomotor activity. (E) Statistical analysis of open arm time, entries, and total arm entries during EPMT. (F) Statistical analysis of latency to the light side and time on the light side during the LDT. (G) Representative heat map of exploratory activity, time in the center, and total distance travelled during the OFT. female: n = 9 mice, AAV-TPH2-GFP group; n = 9 mice for the AAV-TPH2-Cre-GFP group. (A: Mann-Whitney U test; B left, C, D, E left, E middle, E right, F left, F right, G left, G right: two-tailed unpaired Student’s t test; B right: two-way ANOVA followed by Holm-sidak’s post-hoc test.) Data are presented as the means ± S.E.M.

**Figure S15. Depression-like and anxiety-like behaviors in male mice with knockout of AMPKα2 in GABAergic neurons of the DRN**.

(A) Schematic representation of the experimental procedure for *AMPKα2*^flox/flox^ mice, with virus injection of AAV-Vgat-Cre-GFP or AAV-Vgat-GFP. (B) Representative images showing GFP expression and co-staining with GABA (red) in the DRN. Scale bars = 100μm (left), 50μm (right). (C) Western blot images and (D) quantification of AMPKα2 in the DRN of *AMPKα2*^flox/flox^ mice with virus injection of AAV-Vgat-Cre-GFP or AAV-Vgat-GFP. n = 7 for the AAV-Vgat-GFP group; n = 5 for the AAV-Vgat-Cre-GFP group. (D: two-tailed unpaired Student’s t test.) (E) Statistical analysis of sniffing time for female urine in the FUST. (F) Statistical analysis of sucrose preference in the SPT. (G) Statistical analysis of latency to feed and food intake in the NSFT. (H) Statistical analysis of immobility time in the FST. (I) Statistical analysis of open arms time, entries, total arms entries during EPMT. (J) Statistical analysis of latency on the light side and time on the light side during LDT. (K) Representative heat map of exploratory activity, time in the center, and total distance travelled during the OFT. (L) Representative activity tracking and total distance travelled during locomotor activity. Males: n = 10 mice in the AAV-Vgat-GFP group, n = 10 mice in the AAV-Vgat-Cre-GFP group. (E, G right: two-way ANOVA followed by Holm-sidak’s post-hoc test; F, G left, H, I right, J left, J right and L: two-tailed unpaired Student’s t test; I left, I middle, K left and K right: Mann-Whitney U test.) Data are presented as the means ± S.E.M. ***P* < 0.01.

**Figure S16. Depression-like and anxiety-like behaviors in female mice with knockout of AMPKα2 in GABAergic neurons of the DRN**.

(A) Statistical analysis of sucrose preference in the SPT. (B) Statistical analysis of latency to feed and food intake in the NSFT. (C) Statistical analysis of immobility time in the FST. (D) Statistical analysis of open arms time, entries, total arms entries during EPMT. (E) Statistical analysis of latency to the light side and time on the light side during the LDT. (F) Representative heat map of exploratory activity, time in the center, and total distance travelled during the OFT. (G) Representative activity tracking, and the total distance travelled during locomotor activity. Females: AAV-Vgat-GFP group, n = 10 mice; AAV-Vgat-Cre-GFP group, n = 10. Data are presented as the means ± S.E.M. **P* < 0.05. (A: Mann-Whitney U test; B left, C, D left, D middle, D right, E left, E right, F left, F right, G: two-tailed unpaired Student’s t test; B right: two-way ANOVA followed by Holm-sidak’s post-hoc test.)

**Figure S17. Depression-like and anxiety-like behaviors in male mice with knockout of AMPKα2 in dopaminergic neurons of the DRN**.

(A) Schematic representation of the experimental procedure for *AMPKα2*^flox/flox^ mice, with virus injection of AAV-TH-Cre-GFP or AAV-TH-GFP. (B) Representative images showing GFP expression and co-staining with TH(red) in the DRN. Scale bars =100μm (left), 50μm (right). (C) Western blot images and (D) quantification of AMPKα2 in the DRN of *AMPKα2*^flox/flox^ mice with virus injection of AAV-TH-Cre-GFP or AAV-TH-GFP. n = 7 for AAV-TH-GFP group and n = 5 for AAV-TH-Cre-GFP group. D: two-tailed unpaired Student’s t test. (E) Statistical analysis of sniffing time for female urine in the FUST. (F) Statistical analysis of sucrose preference in the SPT. (G) Statistical analysis of latency to feed and food intake in the NSFT. (H) Statistical analysis of immobility time in the FST. (I) Statistical analysis of open arms time, entries, and total arms entries during EPMT. (J) Statistical analysis of latency on the light side and time on the light side during LDT. (K) Representative heat map of exploratory activity, time in the center, and total distance travelled during the OFT. (L) Representative activity tracking and total distance travelled during locomotor activity. Male mice: n = 10 in the AAV-TH-GFP group and n = 10 in the AAV-TH-Cre-GFP group. (E, G lower: two-way ANOVA followed by Holm-sidak’s post-hoc test; F, G upper, I left, I middle: Mann-Whitney U test; H, I right, J upper, K left, K right, L: two-tailed unpaired Student’s t test; J lower: two-tailed unpaired t test with Welch’s correction.) Data are presented as the means ± S.E.M. ****P* < 0.001.

**Figure S18. Depression-like and anxiety-like behaviors in female mice** **with knockout of AMPKα2 in dopaminergic neurons of the DRN**.

(A) Statistical analysis of sucrose preference in the SPT. (B) Statistical analysis of latency to feed and food intake in the NSFT. (C) Statistical analysis of immobility time in the FST. (D) Statistical analysis of open arm time, entries, and total arm entries during EPMT. (E) Statistical analysis of latency to the light side and time on the light side during the LDT. (F) Representative heat map of exploratory activity, time in the center, and total distance travelled during the OFT. (G) Representative activity tracking, and the total distance travelled during locomotor activity. Females: n = 10 mice in the AAV-TH-GFP group, n = 10 mice in the AAV-TH-Cre-GFP group. (A, C, D left, D middle, D right, E right, F left, G: two-tailed unpaired Student’s t test; B left, E left, F right: Mann-Whitney U test; B right: two-way ANOVA followed by Holm-sidak’s post-hoc test.) Data are presented as the means ± S.E.M.

**Figure S19. Locomotor activity of AMPKα2 restoration in serotonergic neurons in the DRN**.

(A) Representative activity tracking and total distance travelled during locomotor activity in the *AMPKα2* CKO mice with injection of AAV-DIO-*AMPKα2*-GFP or AAV-DIO-GFP. male: n = 10 mice for the AAV-DIO-GFP, n = 10 mice for the AAV-DIO-*AMPKα2*-GFP group. (A: two-tailed unpaired Student’s t test.) (B) Representative activity tracking and total distance travelled during locomotor activity in the *ePet*-Cre mice with injection of AAV-DIO-*AMPKα2*-GFP or AAV-DIO-GFP. male: n = 10 mice for the Control + AAV-DIO-GFP, n = 10 mice for the CUS + AAV-DIO-GFP group, n = 10 mice for the CUS + AAV-DIO-*AMPKα2* group. (B: one-way ANOVA followed by Sidak’s multiple comparison test.) Data are presented as the means ± S.E.M.

**Figure S20. Depression-like behaviors following AMPKα2 overexpression in DRN serotonergic neurons in female mice**.

(A) Statistical analysis of sucrose preference in the SPT. (B) Statistical analysis of latency to feed and food intake in the NSFT. (C) Statistical analysis of immobility time in the FST. (D) Representative activity tracking and total distance travelled during locomotor activity. Female: n = 10 mice for the Control + AAV-DIO-GFP, n = 10 mice for the CUS+AAV-DIO-GFP group, n = 10 mice for the CUS + AAV-DIO-*AMPKα2* group. (A, C, D: one-way ANOVA followed by Sidak’s multiple comparison test; B left: Kruskal-Wallis test followed by Dunn’s post hoc test; B right: two-way ANOVA followed by Holm-sidak’s post-hoc test.) Data are presented as the means ± S.E.M.

**Figure S21. Locomotor activity following Foxc2 overexpression in DRN serotonergic neurons of *AMPKα2* CKO mice**.

Representative activity tracking and total distance travelled during locomotor activity in the *AMPKα2* CKO mice with injection of AAV-DIO-*Foxc2*-GFP or AAV-DIO-GFP. Male: n = 10 mice for the AAV-DIO-GFP group; n = 9 mice for the AAV-DIO-*Foxc2*-GFP group. ( Two-tailed unpaired Student’s t test.) Data are presented as the means ± S.E.M.

**Figure S22. Depression-like behaviors following Foxc2 overexpression in DRN serotonergic neurons in male mice**.

(A) Schematic representation of the experimental procedure for *ePet*-Cre mice injected with AAV-DIO-*Foxc2*-GFP or AAV-DIO-GFP. (B) Representative images showing GFP expression in the DRN. Scale bars =100μm (left), 50μm (right). (C) Statistical analysis of the sniffing time for female urine in FUST. (D) Statistical analysis of sucrose preference in the SPT. (E) Statistical analysis of latency to feed and food intake in the NSFT. (F) Statistical analysis of immobility time in the FST. (G) Representative activity tracking, and the total distance travelled during locomotor activity. Male: n = 10 mice in the Control + AAV-DIO-GFP group, n = 10 mice in the CUS + AAV-DIO-GFP group, n = 10 mice in the CUS+AAV-DIO-*Foxc2* group. (C, F: Kruskal-Wallis test followed by Dunn’s post hoc test; D, E upper, G: one-way ANOVA followed by Sidak’s multiple comparison test; E lower: two-way ANOVA followed by Holm-sidak’s post-hoc test.) Data are presented as the means ± S.E.M. *P < 0.05, **P < 0.01, and ***P < 0.001.

**Figure S23. Depression-like behaviors following Foxc2 overexpression in DRN serotonergic neurons in female mice**.

(A) Statistical analysis of sucrose preference in the SPT. (B) Statistical analysis of latency to feed and food intake in the NSFT. (C) Statistical analysis of immobility time in the FST. (D) Representative activity tracking and total distance travelled during locomotor activity. Female: n = 10 mice in the Control + AAV-DIO-GFP group, n = 10 mice in the CUS + AAV-DIO-GFP group, n = 10 mice in the CUS + AAV-DIO-*Foxc2* group. (A, B left, C, D: one-way ANOVA followed by Sidak’s multiple comparison test; B right: two-way ANOVA followed by Holm-sidak’s post-hoc test.) Data are presented as the means ± S.E.M. *P < 0.05.

**Figure S24. Deletion of Foxc2 in serotonergic neurons of the DRN leads to depression-like behaviors in male mice**.

(A) Schematic representation of the experimental procedure for *ePet*-Cre mice injected with AAV-DIO-sh*Foxc2*-GFP or AAV-DIO-GFP. (B) Representative images showing GFP expression in the DRN. Scale bars =100μm (left), 50μm (right). (C) Western blot images and quantification of Foxc2 in the DRN of *ePet*-Cre mice injected with AAV-DIO-sh*Foxc2*-GFP or AAV-DIO-GFP. n = 6 for the AAV-DIO-GFP group and n=7 for the AAV-DIO-sh*Foxc2*-GFP group. (Two-tailed unpaired Student’s t test.) (D) Statistical analysis of sniffing time for female urine in the FUST. (E) Statistical analysis of sucrose preference in the SPT. (F) Statistical analysis of the latency to feed and food intake in the NSFT. (G) Statistical analysis of immobility time in the FST. (H) Statistical analysis of open arms time, entries, and total arms entries during EPMT. (I) Statistical analysis of latency to the light side and time on the light side during the LDT. (J) Representative heat map of exploratory activity, time in the center, and total distance travelled during the OFT. (K) Representative activity tracking and total distance travelled during locomotor activity. Male mice: n = 9 in the AAV-DIO-GFP group and n = 9 in the AAV-DIO-sh*Foxc2*-GFP group. (D, F right: two-way ANOVA followed by Holm-sidak’s post-hoc test; E, G, H middle, H right, I right, J left, J right, K: two-tailed unpaired Student’s t test; F left, H left, I left: Mann-Whitney U test.) Data are presented as the means ± S.E.M. **P* < 0.05, ****P* < 0.001.

**Figure S25. Depression-like and anxiety-like behaviors in female mice with knockout of Foxc2 in serotonergic neurons of the DRN**.

(A) Statistical analysis of sucrose preference in the SPT. (B) Statistical analysis of latency to feed and food intake in the NSFT. (C) Statistical analysis of immobility time in the FST. (D) Statistical analysis of open arms time, entries, total arms entries during EPMT. (E) Statistical analysis of latency to the light side and time on the light side during the LDT. (F) Representative heat map of exploratory activity, time in the center, and total distance travelled during the OFT. (G) Representative activity tracking, and the total distance travelled during locomotor activity. Female: n=10 mice for the AAV-DIO-GFP group, n=10 mice for the AAV-DIO-sh*Foxc2*-GFP group. (A, B left, C, D middle, D right, E right, F left, F right: two-tailed unpaired Student’s t test; B right: two-way ANOVA followed by Holm-sidak’s post-hoc test; D left, E left, G: Mann-Whitney U test.) Data are presented as the means ± S.E.M. **P* < 0.05.

**Figure S26. Transcriptome analysis of DRN tissue from male mice with serotonergic neuron-specific Foxc2 knockdown**.

(A) Volcano plots of differentially expressed genes in RNA sequencing analysis. (B) GO enrichment analysis. (C) KEGG pathway enrichment analysis.

**Figure S27. Prediction of phosphorylation sites in the KAT7 protein**.

(A) GPS_6.0 predicted potential phosphorylation sites in the KAT7 protein mediated by AMPKα2 kinase. (B) Immunoblot analysis of whole-cell lysates (input) and anti-Flag immunoprecipitates derived from HT22 cells transfected with Flag-KAT7. (C to F) Binding levels of KAT7 at H2-Q1(C), Slc6a12(D), Wnt6(E), Slc22a6(F) promoters in HT22 cells following AMPKα2 knockdown. n = 4 per group. (C, D, E, F: two-way ANOVA followed by Holm-sidak’s post-hoc test.) Data are presented as the means ± S.E.M.

**Table S1. Mt1 peptides sequences .**

|  | Peptides sequence |
| --- | --- |
| Mt1-1 | 15-CTSSCACKNCKCTS-28 |
| Mt1-2 | 45-AQGCVCKGAADKCT-58 |

**Table S2. Sequences of siRNA oligos used in this study.**

| siRNA | Sense | Antisense |
| --- | --- | --- |
| Megalin siRNA | GCUAUUGUAUUAGAUCCUUtt | AAGGAUCUAAUACAAUAGCtc |

**Table S3. Target sequences of shRNA.**

| shRNA | Target sequence |
| --- | --- |
| SCTR | CCATCGTGATCAATTTCAT |
| AMPKα1 | GCACGAGTTGACCGGACATAA |
| Foxc2 | CCACACGTTTGCAACCCAA |
| AMPKα2 | GAGCGACTATCAAAGACATACGAGA |

**Table S4. Primer sequences.**

| Gene | Sense primer | Antisense primer |
| --- | --- | --- |
| mMegalin | CCCTTGCAAGCAGGTCTGTA | GCATGGTGATGGGGAGTTCA |
| mFoxc2 | GCCCAACTGTTACTGCCAAAT | CAAAGCCATGCACTTCCTAA |
| β-tubulin | AGCAACATGAATGACCTGGTG | GCTTTCCCTAACCTGCTTGG |
| Foxc2 promoter-1  (-970~-712) | CGCGCTCCTTTGCGTTTCCA | GTCCTCCAAACAATTTCGTG |
| Foxc2 promoter-2  (-711~-456) | TCAGATGGATCACCTAAGT | GCCTAGTTGTAATGGGTT |
| Foxc2 promoter-3  (-455~-168) | ACAACTAGGCCCCGATAA | CCCCCCAGCTCAAGTTAA |
| Foxc2 promoter-4  (-167~-1) | GGGGGAGATCTGGCTAAGA | AAAAGTCTTCTGGCTCAA |
| H2Q1 promoter-1  (-900~-653) | CTTTATCAGTCCCTCCCG | GCAAACAGCACAGCCTCC |
| H2Q1 promoter-2  (-501~-220) | GTGGTCTTGGCTGGTGAT | CAGGGAACAGAGTGAGGC |
| Slc6a12 promoter-1  (-681~-417) | TTTGCATTTGCCTTCCTG | CCAACTACCGGCTCCTTC |
| Slc6a12 promoter-2  (-415~-143) | GTGGAGGTGAGGATAAGGG | GGCTGTAGAAAGTGAAGGGA |
| Wnt6 promoter-1  (-855~-591) | GCTTGGCTTGATGAATGGA | AGGGCCGAGGTTGCTGACT |
| Wnt6 promoter-2  (-411~-143) | AAAGACGCGGGAGTTGGT | GGGATGAATAGTCGAGAAGGGT |
| Slc22a6 promoter-1  (-717~-422) | AGCTCCAGAGTTGTAATG | CTGGGAGTAGTGTATTCTTT |
| Slc22a6 promoter-2  (-375~-112) | CTGTTCAATATCAGGGCTAC | GATGGATGAGACTGGGAG |

| Probe | Sequence |
| --- | --- |
| SCTR | GAGCAGACGGTGAGAATACGAT |
| Mt1 | ACACAGCCCTGGGCACATTTGG |

**Table S5. Probe sequences.**

**Dataset S1. (separate file).**

Lists of differentially expressed protein statistics from the DRN of *Aldh1l1*-Cre mice with virus injection of AAV-DIO-shSCTR-GFP or control AAV-DIO-GFP.

**Dataset S2. (separate file).**

Lists of DEG statistics from the DRN of AMPKα2 CKO mice and WT mice. CKO mice: *AMPKα2*^flox/flox; ePet-Cre^ mice; WT mice: *AMPKα2*^flox/flox^ mice.

**Dataset S3. (separate file).**

Lists of DEG statistics from the DRN of *ePet*-Cre mice with virus injection of AAV-DIO-sh*Foxc2*-GFP or control AAV-DIO-GFP.

**Dataset S4. (separate file).**

Mass spectrometry dataset of the phosphorylated peptides generated by in vitro phosphorylation following AMPKα2 knockdown.
